# Organic Solvent Nanofiltration in Pharmaceutical Applications

**DOI:** 10.1021/acs.oprd.3c00470

**Published:** 2024-03-22

**Authors:** Hui Xiao, Yanyue Feng, William R. F. Goundry, Staffan Karlsson

**Affiliations:** † Early Chemical Development, Pharmaceutical Sciences, Biopharmaceuticals R&D, AstraZeneca, Macclesfield SK10 2NA, United Kingdom; ‡ Early Chemical Development, Pharmaceutical Sciences, Biopharmaceuticals R&D, AstraZeneca Gothenburg, SE-431 83 Mölndal, Sweden

**Keywords:** organic solvent nanofiltration, solvent exchange, solvent recovery, catalyst recovery, concentration, purification, pharmaceuticals

## Abstract

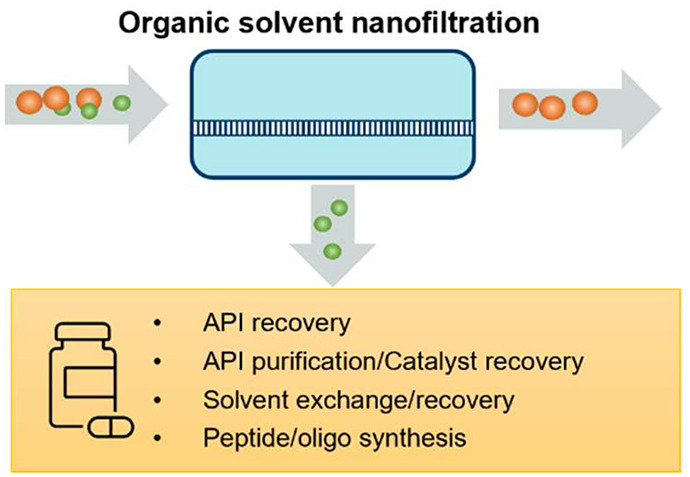

Separation and purification in organic solvents are indispensable procedures in pharmaceutical manufacturing. However, they still heavily rely on the conventional separation technologies of distillation and chromatography, resulting in high energy and massive solvent consumption. As an alternative, organic solvent nanofiltration (OSN) offers the benefits of low energy consumption, low solid waste generation, and easy scale-up and incorporation into continuous processes. Thus, there is a growing interest in employing membrane technology in the pharmaceutical area to improve process sustainability and energy efficiency. This Review comprehensively summarizes the recent progress (especially the last 10 years) of organic solvent nanofiltration and its applications in the pharmaceutical industry, including the concentration and purification of active pharmaceutical ingredients, homogeneous catalyst recovery, solvent exchange and recovery, and OSN-assisted peptide/oligonucleotide synthesis. Furthermore, the challenges and future perspectives of membrane technology in pharmaceutical applications are discussed in detail.

## 1 Introduction

Separation and purification are almost indispensable procedures for high-purity products in the chemical, petrochemical, pharmaceutical, and food industries.^1^ However, the energy consumption for those separations, which heavily rely on energy-intensive distillation, accounts for 10–15% of global energy consumption.^2^ Membrane separation as an alternative to distillation has attracted extensive attention due to its low energy consumption and carbon footprint.^3^ For example, the successful large-scale applications of reverse osmosis membranes have been used to produce fresh water from seawater in water-stressed countries.^4^ Besides the successful membrane applications in aqueous solutions, membrane separations in organic solvents are also in high demand, because extensive quantities of organic solvents, which are used for reactions, extraction and purification processes in the chemical and pharmaceutical industries, need to be recovered or discarded at some point.^5^ Also, some high-value products dissolved in the solvents need to be recovered.

Organic solvent nanofiltration (OSN) is an emerging technology for energy-efficient solvent separations, which can be used for the following: rejecting solutes from 200 to 1000 g mol^–1^, solvent exchange, and solvent recovery.^6−8^ OSN is also known as solvent-resistant nanofiltration or organophilic nanofiltration. It has the potential to become the best available technology for organic solvent purification due to the following advantages: (i) low energy requirement; (ii) low solid waste generation (compared to solid waste of silica gels for chromatography and adsorbents for adsorption); (iii) mild temperature and pressure operating conditions; (iv) straightforward scale-up possibilities; (v) chemical stability in harsh environments allowing flexibility of choice of pH, temperature, and solvent; and (vi) easy solvent exchange from a low to high boiling point solvent and vice versa.^3^

**Figure 1:**
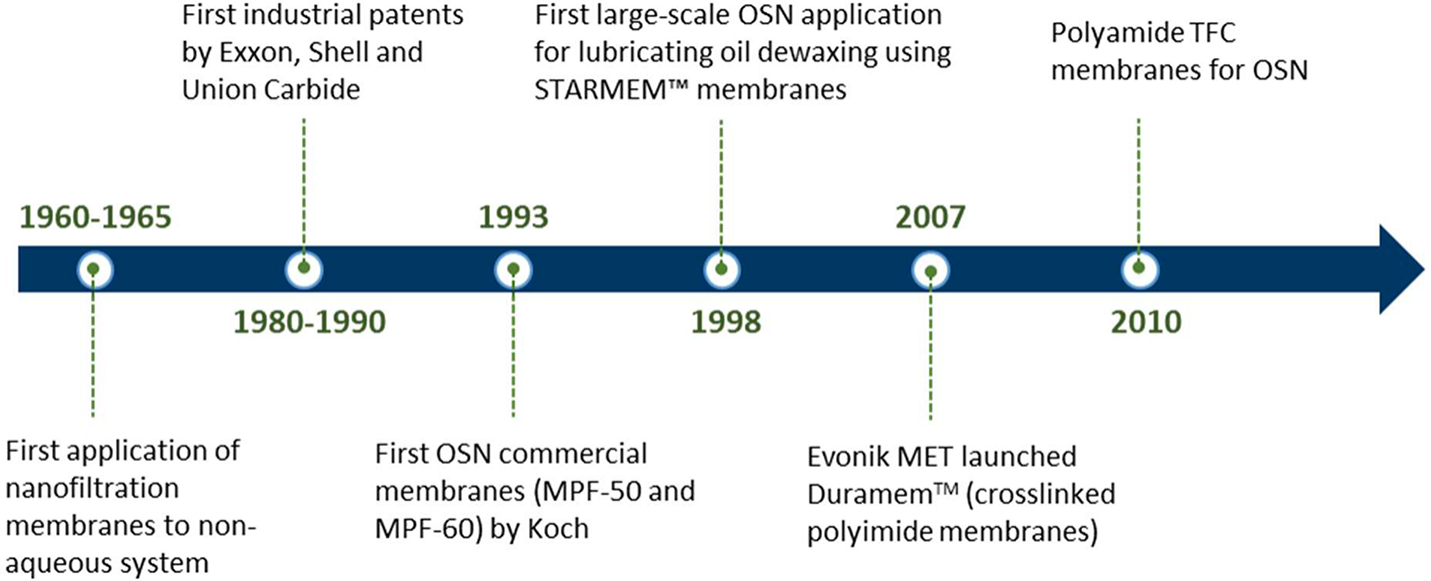
Milestones of the developments of OSN.

The history of OSN (Figure 1) can be traced back to the introduction of asymmetric cellulose acetate membranes to aqueous applications in the 1960s by the pioneering work of Loeb and Sourirajan.^9^ In 1964, these membranes were applied for the separation of hydrocarbon solvent mixtures, which was the first application of OSN membranes in nonaqueous systems.^10^ In the 1990s, the Koch Membrane System was the first company that commercialized OSN membranes (MPF-50 and MPF-60) based on polydimethylsiloxane (PDMS), which were stable in most organic solvents. Then more commercial products appeared on the market by Grace Davison, Koch, Solsep, and GMT companies. The first large-scale OSN application was for solvent recovery in Exxon Mobil’s Max-Dewax process using STARMEN^TM^ series membranes from Grace Davison Membranes.^11^ Unlike the pure polyimide-based STARMEN^TM^ series membranes, DuraMem^TM^ are a series of cross-linked polyimide membranes with better organic solvent resistance in polar aprotic solvents, produced by Evonik Membrane Extraction Technology (MET) Ltd. in 2007. In 2010, a new generation of polyamide-based thin film composite (TFC) membranes for OSN was reported and showed improved performance in polar aprotic solvents,^12^ expanding the OSN membranes’ research scope.

**Figure 2:**
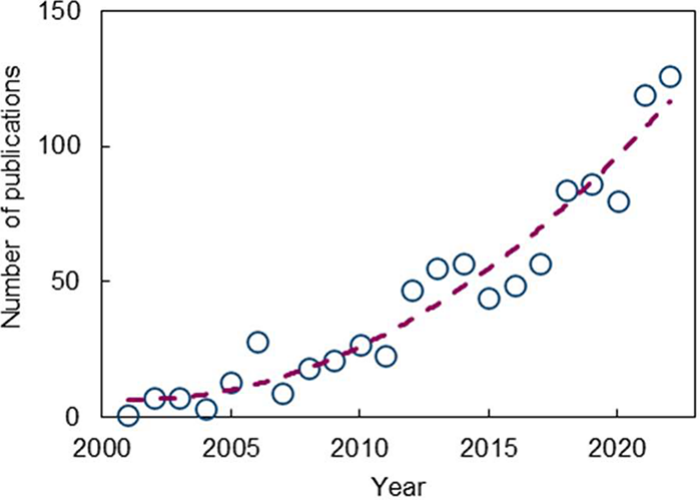
Number of publications by year for the period of 1999–2022. The search was carried out on 29/03/2023 in Scopus using keywords of “organic solvent nanofiltration” OR “solvent resistant nanofiltration” OR “organophilic nanofiltration”.

According to research in Scopus (Figure 2), the number of OSN-related publications in the last two decades shows a dramatic increase, with more than 80% of these papers published over the last ten years, showing a rising interest in this technology in academia and industry. There have been some interesting review articles on OSN technology.^13−17^ However, most of them focused only on the development of membrane materials, and there has been a lack of specific reviews focusing on the application of OSN in the pharmaceutical area. Thus, this Review aims at providing a summary of recent developments of OSN and its specific applications in the pharmaceutical industry, including API concentration and purification, homogeneous catalyst recovery, solvent exchange and recovery, and OSN-assisted peptide/oligonucleotide synthesis. A brief introduction to OSN including typical membrane processes, membrane performance characterization and separation mechanism, as well as an overview of different types of OSN membranes and commercially available OSN membranes, are given. Furthermore, the challenges and future perspectives of employing OSN technology in pharmaceutical manufacturing will be discussed. This comprehensive Review may help scientists and engineers identify possible membrane opportunities and increase the adoption of OSN technology in the pharmaceutical industry.

## 2 Fundamentals of OSN

### 2.1 Typical Membrane Processes

**Figure 3:**
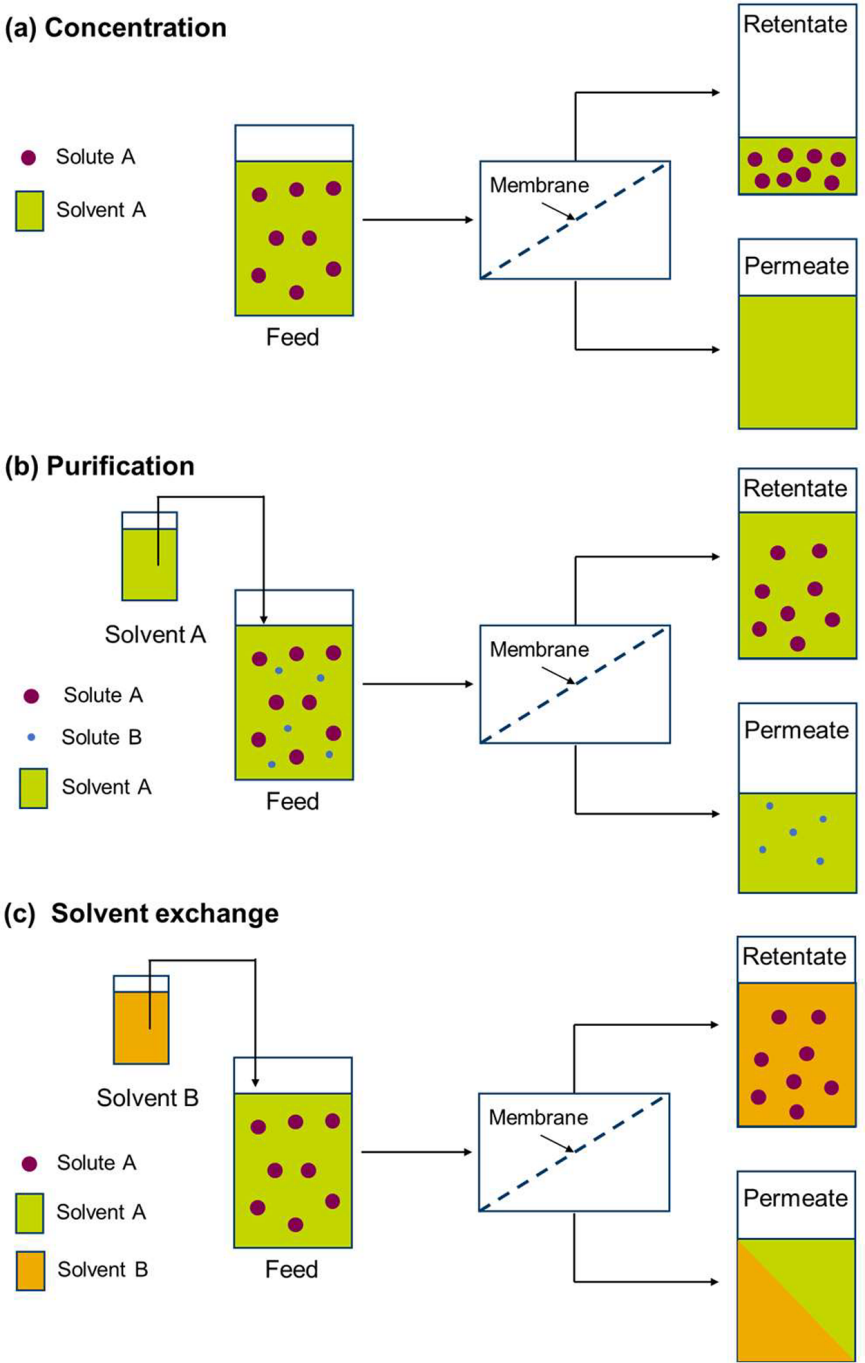
Operating modes of organic solvent nanofiltration.

There are three basic process options for OSN operations: concentration, solvent exchange, and purification (Figure 3).^6^ In the concentration process, the solute is rejected and concentrated by the membrane, while the solvent passes through the membrane freely. Through the concentration process, we can either recover high value products from a dilute solution (such as solute concentration and catalyst recovery) or recover solvent by removing the solute impurities (solvent recovery). A suitable membrane for a concentration process should hold adequately high rejection toward the solute but let the solvent permeate through freely. Solvent exchange is used to replace the original solvent A in solution with a second solvent B by using diafiltration mode, where solvent B is added to the retentate at the same rate as the permeate is generated. Like the concentration process, the solvent exchange also requires a tight membrane to reject all the solutes but allow the solvent to pass through. Furthermore, if the membrane can retain more new solvent B than the old solvent A, the solvent exchange process will be more efficient. Membrane separation is particularly attractive for solvent exchange from a high-boiling to a low-boiling solvent, where the traditional method typically requires several repeated cycles of concentration by distillation and solvent addition steps. In purification, the emphasis of the process is the separation of two (or more) solutes in a solution, for example, the products and byproducts of a reaction. The goal of the purification is to retain one solute by the membrane while permeating another solute, and the solvent here acts as the carrier to wash out the more permeable solute through the membrane continuously. A significant rejection difference between the two solutes is crucial for the process feasibility. That means a large difference in their molecular weights (>200 Da) is normally required to ensure a successful separation.

These membrane operations can be further optimized by using multistage membrane cascades (multiple membrane modules connected in parallels or series), hybrid processes (combining membranes with other separation techniques) and continuous operations.

### 2.2 Membrane Performance Characterization

Flux (or permeance) and rejection are two important parameters to describe the performance of membranes. Flux (*j*) is defined as the volume of the liquid (*V*) passing through the membrane per surface area (*A*) and time (*t*) by equation 1,
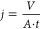
1Flux can be further normalized by the applied pressure, which generates the permeance.

Rejection (*R*) is normally calculated as a function of the solute concentration in the permeate (*C*_p_) and retentate (*C*_r_) by equation 2,
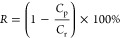
2There are many factors affecting the membrane flux and rejection, including the membrane type, solvent system, solute type and concentration, and process parameters (operating temperature, pressure, cross-flow velocity, pH, etc.).^18^ For example, solution concentration is a critical yet often neglected factor in membrane studies. Many papers only present the membrane performance data in dilute solutions, which may not fully reflect the complexities of real-world applications with higher solute concentrations.^19^ Higher solute concentrations typically lead to lower flux and rejection.^20,21^ Furthermore, higher solute concentrations might exacerbate membrane fouling, ultimately reducing both the membrane performance and longevity. Consequently, it is essential to extend investigations beyond dilute solutions and explore the membrane performance in real conditions before implementing membrane applications in industrial settings.

It is worth mentioning that membranes normally demonstrate a tradeoff between the flux and rejection,^22^ which means that membranes with high rejections usually have low fluxes and vice versa. However, both high rejection and large flux are eagerly pursued for industrial applications, as high rejection means high product yield, and large permeate flux can lower the membrane area required and thereby reduce capital expenditure.^23^ Also, membrane flux decline with time is often observed, mainly due to concentration polarization and membrane fouling.^20,21^ Concentration polarization occurs when the concentration of solutes increases near the membrane surface due to selective transport through the nanofiltration membrane.^20,21^ The accumulation of retained solutes at the membrane surface increases the osmotic pressure, subsequently offsetting the driving pressure and causing a reduction in flux. Although concentration polarization is an inherent phenomenon limiting the membrane performance, it can be mitigated by increasing the turbulence of the feed fluid, such as employing a high cross-flow velocity or stirring rate.^24^ However, concentration polarization may cause membrane fouling, which seriously diminishes the membrane’s performance and longevity.

**Figure 4:**
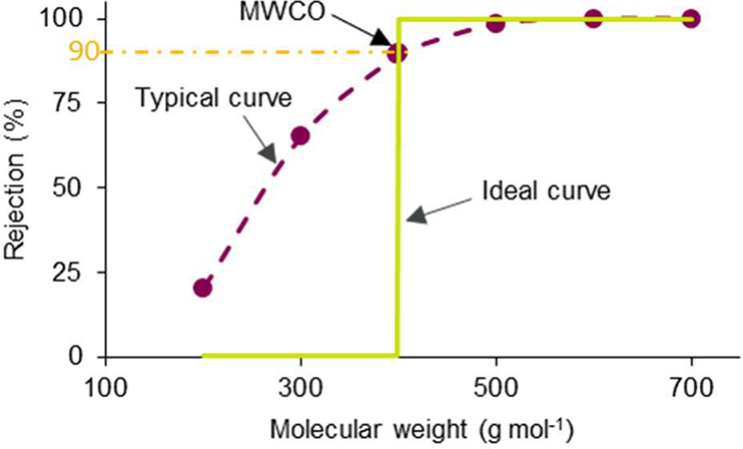
A typical MWCO curve for membranes with a MWCO of 400 g mol^–1^.

Another important parameter for membrane performance characterization is molecular weight cutoff (MWCO), which is the molecular weight (MW) of the reference compound rejected by 90%. Figure 4 presents a plot of the molecular weight of reference compounds vs the membrane rejections (namely MWCO curves), where the MWCO of the membrane is derived. An ideal vertical MWCO curve represents a neat separation between two solutes with a rejection of either zero or 100%. Although an ideal MWCO curve is always pursued by membrane scientists and engineers, the predicted rejection profiles by Marchetti et al. suggested that ideal separation is impossible even for membranes with uniform pore size.^25^ In reality, the OSN membrane normally has a broad MWCO curve, where the rejections go up slowly with the increase of MWs.

One thing that should be noted is that although MWCO is the most common way to describe the performance of nanofiltration membranes in aqueous solution, it is not sufficient to use MWCO alone to compare the membrane performance in organic solvents.^26^ Many factors, such as the shape, charge and solubility of the solute, different solute and solvent mixtures, and even the experimental setup can affect the values of MWCO.^27^ Various commonly used solutes (such as dyes, oligomers, n-alkanes, esters, triglycerides, sugars and inorganic salts) were adopted by researchers to measure the MWCO for OSN membranes.^27^ However, the properties of those solutes (such as configuration, charges or sizes) largely depend on the test environment (such as solvent types and solute concentration),^27^ which will affect the accuracy of the MWCO. Verbeke et al.^27^ proposed some solutes with less environment-dependent properties, including dendrimers, hyperbranched oligomers, homogeneous catalysts, and derivatized sugars, as alternatives to measure MWCO for membranes. Considering there is still no consensus on a standard test method for MWCO, it is suggested that a proper comparison should be conducted in the presence of the same testing systems (solute and solvent mixtures).

### 2.3 Membrane Separation Mechanism

**Figure 5:**
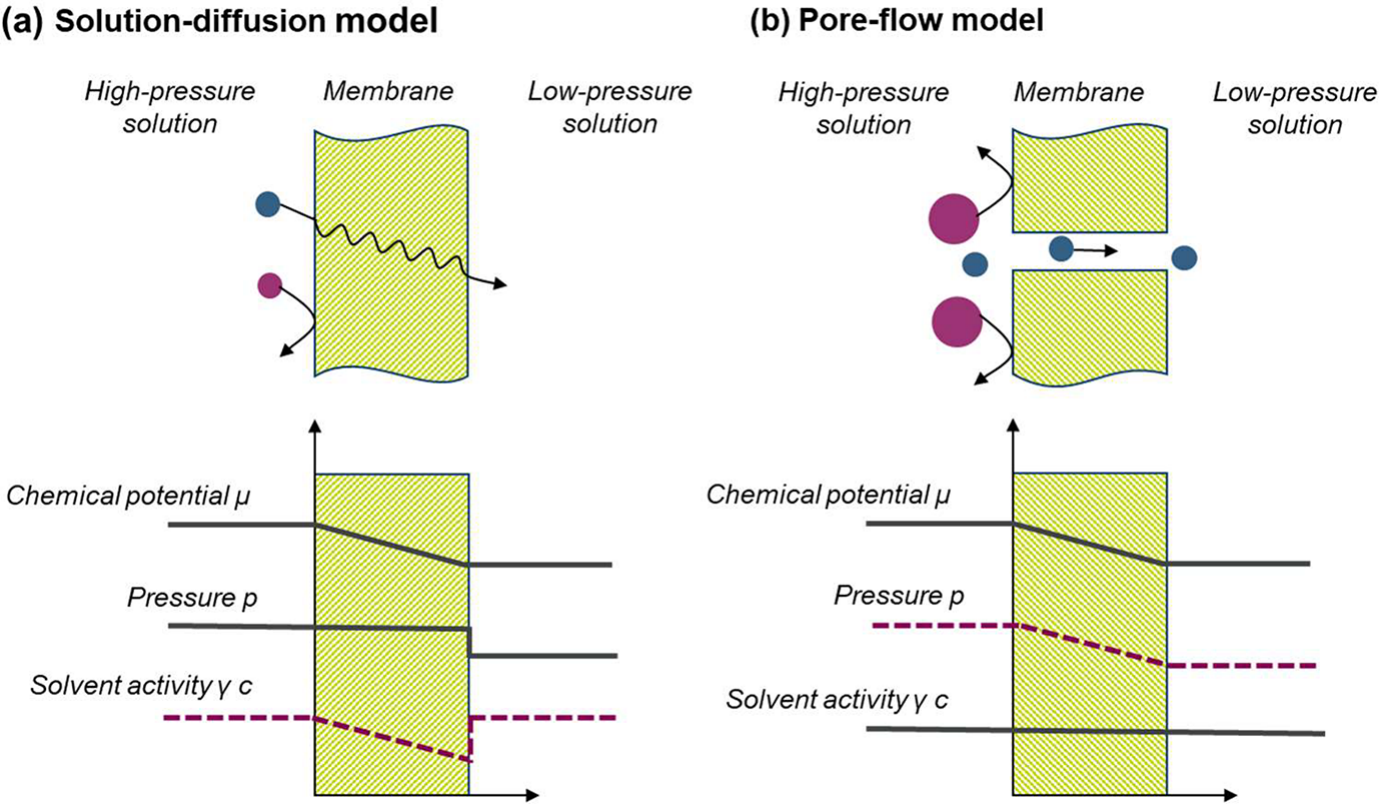
Molecular transport through membranes according to (a) solution-diffusion and (b) pore-flow models. Reproduced with permission from ref (28). Copyright 1995 Elsevier.

Two mathematical transport models were proposed to describe the transport of solutes through membranes: solution-diffusion and pore-flow models.^28^ As shown in Figure 5, the separation of the pore-flow model is based on the pore size in the membrane: solutes smaller than these pores can pass, while those larger than pores will be rejected. The solution-diffusion model suggests the transport can be divided into two steps: the dissolution of solutes into a membrane, and the subsequent diffusion through it. The major difference between these two models is the driving force of solute transport: the solution-diffusion model assumes that the pressure within a membrane is constant and that the transport across the membrane is driven by a concentration gradient, while the pore-flow model postulates that the concentrations of solvent and solute within a membrane are uniform and that the transport is governed by a pressure gradient.

**Figure 6:**
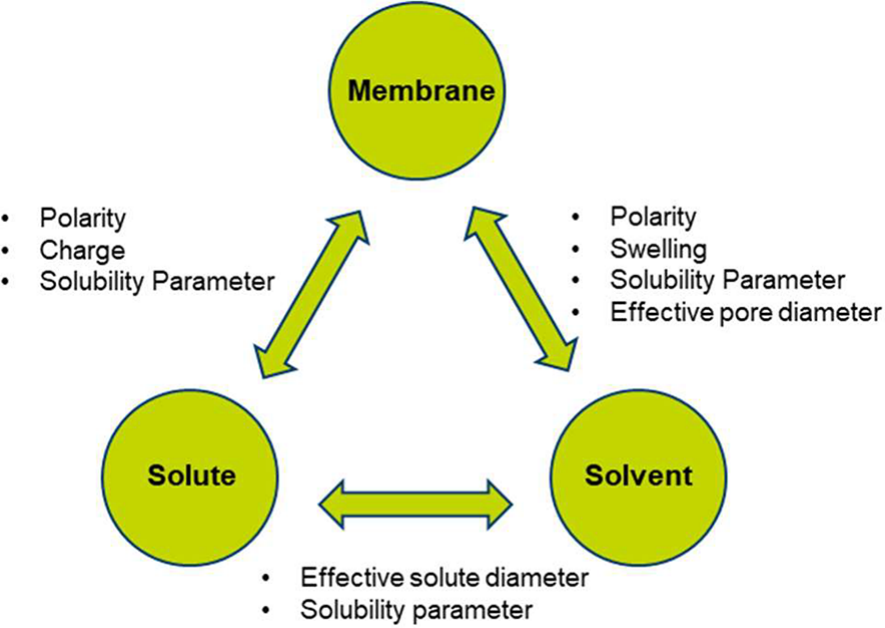
Solute–solvent–membrane interactions affecting the OSN membrane performance. Reproduced from ref (6). Copyright 2014 American Chemical Society.

The interactions between solute, solvent, and membrane can dramatically affect the OSN membrane permeance and rejections.^6^ Due to multiple choices of solvent and mixtures, thereof, the OSN transport mechanism is much more complicated than that of the aqueous nanofiltration. As presented in Figure 6, the solute–solvent–membrane interactions can be classified based on the following effects: (i) effective solute diameter; (ii) pore wettability and effective pore diameter; (iii) solute and solvent polarity; and (iv) charge effects,^6^ which will together determine the overall membrane separation performance.

## 3 OSN Membranes

The OSN membrane is the core part of the OSN membrane units. For practical implementation of OSN processes, e.g., in pharmaceutical manufacturing, excellent chemical, thermal and mechanical stabilities are the key criteria for the selection of proper membranes, as any structural or functional failure during operation would lead to severe malfunction of the OSN system.

Based on their chemical compositions, OSN membranes can be divided into three categories: polymeric membranes, inorganic membranes, and mixed matrix membranes.^6^ Among them, polymer OSN membranes have received great attention in the OSN field because of their good flexibility and tuneable properties, cost-effectiveness, and good accessibility.^29,30^ However, their sometimes unstable properties in organic solvents, resulting in swelling, aging and compaction, must be taken into consideration.^17^ This intrinsic drawback normally arises from the membrane structure and the fabrication route: fabrication of polymeric membranes requires dissolving parent polymers in polar aprotic solvents, and thus dissolution and collapse of as-prepared membranes in those solvents is often inevitable.^31^ To tackle this dilemma, a post-treatment through cross-linking, using chemical agents, thermal or photo energy, is always needed.^32^ Unlike polymeric membranes, inorganic membranes have superior mechanical, thermal and chemical stabilities. They have higher tolerance for high pressure, do not swell in organic solvents, and are easy to clean.^6^ But scaling up the manufacture of inorganic membranes remains more challenging, because they are more brittle, which makes the handling, transport and operation more difficult, and they are less adaptable to different shapes and configurations for different applications. Besides, the hydrophilicity of their main components, i.e. metal hydroxide, makes them less suitable for nonpolar solvents. To harness the excellence of both without compromising the performance and scalability, researchers have developed organic–inorganic (mixed matrix) membranes.^6^ However, the agglomeration of inorganic fillers inside the polymer matrix often deteriorates material properties.^33^ Thus, how to control the dispersion of the nanoparticles in polymeric hosts is critical for the development of mixed matrix membranes.

### 3.1 Polymeric Membrane

**Figure 7:**
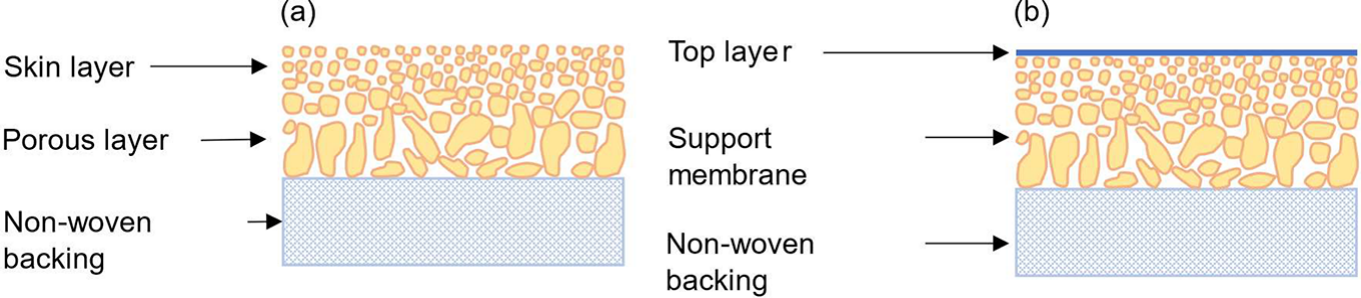
Schematic description of (a) ISA membrane; (b) TFC membrane. Reproduced from ref (6). Copyright 2014 American Chemical Society.

Generally, most polymeric membranes are fabricated on a nonwoven supporting material to achieve mechanical stability. Materials for the nonwoven support should be solvent resistant and have the same properties as the polymeric membrane to avoid crease formation.^6^ There are two main types of polymeric membranes based on structural difference, named integrally skinned asymmetric (ISA) and thin film composite (TFC) membranes. The schematic descriptions of the two types are shown in Figure 7.^6^

#### 3.1.1 Integrally Skinned Asymmetric (ISA) Membrane

ISA membranes have an active skin layer on top of a more porous supporting sublayer with the same composition. Hence, they generally do not suffer from delamination under harsh conditions.^15^ Moreover, they are easy to fabricate and clean/wash, which makes them useful for various industrial applications.^15^ The property of the skin layer is of most importance for the membrane’s selectivity and permeance.^6^

The method for the production of ISA membranes is a phase inversion technique, which was invented by Loeb and Sourirajan in 1962.^9^ This technique includes the precipitation of a casting solution through immersion in a water bath.^9^ A one phase cast solution is precipitated into two phases: polymer-rich solid phase and polymer-poor phase. The polymer-rich phase forms membrane matrix, and the polymer-poor liquid phase forms membrane pores.^6^ The prerequisite for the fabrication of ISA membranes is that the polymer should be soluble in a solvent to form the casting solution, which means that there is a risk that the final membrane will be redissolved in the casting solution once it is formed, leading to poor membrane stability.^6^ To improve stability, some post-treatments can be done such as cross-linking, annealing, and drying.^6^ Typically, materials used for the synthesis of ISA membranes include polyimide,^31^ polybenzimidazole (PBI),^34^ poly(vinylidene fluoride) (PVDF),^35^ poly (ether ether ketone) (PEEK),^36^ epoxy resins,^37^ etc.

#### 3.1.2 Thin Film Composite (TFC) Membrane

TFC membranes contain an ultrathin top layer (50–500 nm) that is cast onto different porous supporting materials. Since the top layer is formed separately from the supporting layer, it is easier to modify and tailor it independently to achieve the desired MWCO and superior solvent permeability and selectivity.^38−40^ The most common supporting materials include asymmetric polyacrylonitrile (PAN), PVDF, polypropylene, polyimide, and PBI. The type of supporting material is of great importance because it affects the mechanical stability and assists in the formation of defect-free top layers.^6^

The main synthesis methods for the top layer of the TFC membrane include (a) depositing a prefabricated ultrathin film onto a support; (b) interfacial polymerization at the surface between the support and the thin layer; (c) dip-coating a reactive monomer solution onto the support, then using heat or irradiation for post-treatment; (d) dip-coating or solvent casting a polymer solution onto support; (e) plasma deposition from a gaseous phase.^41^ Among those methods, interfacial polymerization and dip-coating on a support layer are the most commonly used methods. Polydimethylsiloxane (PDMS) is a typical material for fabricating TFC membranes for OSN applications, which has been commercialized by Evonik, Borsig and SolSep. More information about OSN membrane materials can be found in the review by Shi et al.^15^

### 3.2 Inorganic Membrane

In principle, inorganic membranes are expected to provide more precise results and possess long durability because of their inertness to organic solvents.^42^ Ceramic is a common material for inorganic membranes because they are mechanically, thermally and chemically stable. The most common ceramic membrane materials are Al_2_O_3_, SiO_2_, TiO_2_, and ZrO_2_.^7^ Due to the existence of hydroxyl groups on the membrane surface, the ceramic membranes are hydrophilic, leading to high water flux through the pore. In this case, the nonpolar organic solvents are less applicable because of the low solvent fluxes.^43^ A strategy to increase the low nonpolar solvent fluxes is the surface modification of the top layer with hydrophobic groups. Hosseinabadi and coauthors used Grignard reagents as functional groups to modify the commercially available 1 nm TiO_2_ ceramic membrane surface. The results showed the modified ceramic membranes possess high flux for both polar and nonpolar solvents while maintaining the MWCO. In addition, the retention results of modified ceramic membranes were comparable with the Duramem 300 OSN membrane.^44^ Another study from Hosseinabadi et al. further investigated the retention performance of Grignard functionalized membranes under five different model solvents (polar acetone, nonpolar toluene)/solute (polar polyethylene glycol PEG, nonpolar polystyrene, and catalyst ligand BINAP) systems. All modified ceramic membranes showed enhanced performance than the unmodified ones due to the increased hydrophobicity of the membrane surface. Besides, a four-day experiment showed good stability of modified membrane in acetone/polystyrene.^45^

**Figure 8:**
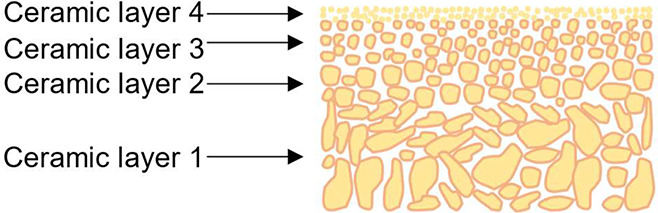
Schematic illustration of ceramic membrane. Reproduced from ref (46). Copyright 2017 VBRI Press.

Ceramic membranes are generally composed of two or more porous layers, forming an asymmetric structure. A thin layer of one or several inner layers is coated onto porous ceramic support through suspension coating. A typical configuration for ceramic membranes is tubular, which is fabricated through the extrusion of ceramic powders together with the addition of plasticizers and binders.^25^ The obtained porous supports are then sintered at high temperatures, to assist with mechanical stability as well as determine the membrane’s external shape.^6^ A schematic illustration of the ceramic membrane is shown in Figure 8.^46^

One of the limitations of producing ceramic membranes is the difficulty of lowering the MWCO to make it suitable for nanofiltration applications. Generally, there are two strategies that could lower the MWCO of ceramic membranes: adding either zeolite or silica particles as the active layers.^47^ So far, the commercial OSN hydrophilic ceramic membrane with the smallest MWCO is Inopornano from Inopor (Germany), of which the MWCO is 200 Da. There are also literature reports of fabricated ceramic membranes that are noncommercial available and have low MWCO. For example, Zeidler et al. prepared multilayer tubular ceramic membranes with active layers of titanium dioxide/zirconium oxide, integrating with carbon on top. The tested MWCO of polystyrene mixture in tetrahydrofuran (THF) was 350 Da.^48^ Zeolites have a highly defined and rigid network of pores. The 0.3–1.3 nm small pore size and their inherent stability make them effective materials for the preparation of OSN membranes.^49^ Four types of zeolite (named Linde type A, faujisite, mordenite, and mobile five) have been extensively investigated in both academic and industrial applications.^50^ Those structures are deposited onto the surface of supporting materials by dipping or vacuum process, forming active layers.^50^ Another method is using a silica active layer. The pore size of silica could be decreased to the nanometer range when cetyltrimethylammonium bromide or sodium dodecyl sulfate is used as a surfactant.^51^

### 3.3 Mixed Matrix Membranes

**Figure 9:**
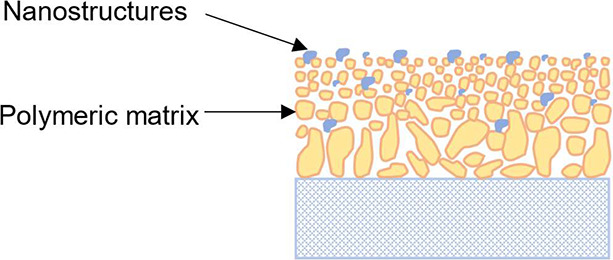
Schematic description of mixed matrix membrane.

Mixed matrix membranes can be regarded as the modification of individual polymeric membranes or inorganic membranes by the addition of some nanostructures, such as nanoparticles, nanotubes, or zeolite, into the polymeric matrix. The obtained mixed matrix membranes are also called nanocomposite membranes and they have the properties of both polymeric membranes and inorganic membranes. For example, they possess good solvent stability, high rejection, and flux, as well as less flux decline and fouling. Moreover, they have enhanced mechanical stability.^6^ They can either be ISA membranes or TFC membranes. The schematic description of the mixed matrix membrane is shown in Figure 9.

The nanoparticles can be added through three different methods: (a) directly adding nanoparticles in the cast solution before the phase inversion process; (b) preformed nanoparticles are deposited onto the membrane surface; (c) the pores of the polymeric matrix are filled with nanoparticles.^6^

### 3.4 Commercially Available OSN Membranes

Despite the rapid development of OSN, the number of OSN membrane suppliers is still limited.^14^ Currently to name a few, companies that provide OSN membranes include Evonik, Borsig, Solsep BV, AMS, and Inopor. The summary of products is listed in Table 1. Those membranes exist in both flat sheet and spiral-wound formats. Flat sheet membranes are usually used in lab tests. The spiral-wound format is the most attractive module at the industrial scale. In this module, a number of flat sheet membranes are wound around a central pipe. The membrane is glued along three sides and the open side is attached to the permeate channel. A permeate spacer is used to provide mechanical resistance, and a feed channel spacer is used to separate the top layers of those membranes.^6^

**Table 1:** Summary of Commercially Available OSN Membranes

Supplier	Series name	MWCO(Da)	Materials and type	Solvent compatibility^a^
Evonik MET	PuraMem Selective	400–500	Cross-linked PDMS on polyimide, TFC	Alcohols, aliphatic hydrocarbons, aromatic hydrocarbons, butyl acetate, ethyl acetate, methyl-ethyl-ketone, methyl-tert-Butyl-Ether
	PuraMem Performance			
	PuraMem Flux			
BORSIG Membrane Technology GmbH	oNF-1	600	PDMS layer on PAN, TFC	Alkanes, aromatics, alcohols, ethers, ketones, esters
	oNF-2	350		
	oNF-3	900		
AMS(Unisol)	NanoPro S-3011	100	N/A	Methanol, ethanol, propanol, hexane, THF, acetone, acetonitrile, ethyl acetate, DMF
	NanoPro S-3012	180		
	NanoPro S-3014	400		
Solsep	010306	500–1000	PDMS	Alcohols, esters, ketones, aromatics, chlorinated, THF
	030306	500–1000		
Inopor GmbH	Inopor nano 1.0 nm	750	TiO_2_	
	Inopor nano 0.9 nm	450		
	Inopor nano LC	200		

a According to the manufacturer’s information

## 4 Pharmaceutical Applications

In the pharmaceutical industry, APIs can be manufactured via chemical synthesis, fermentation, extraction from natural and biological products or a combination of these approaches.^52^ There are many separation and purification steps where OSN can be applied. In terms of the operation modes (Section 2.1), the concentration process of OSN can be used for API/intermediate concentration and solvent recovery/recycling. Purification processes can be used for impurity removal, catalyst recovery/recycling, and OSN-assisted peptide/oligonucleotide synthesis. Also, membranes for solvent exchange can be used. This section will focus on these OSN pharmaceutical applications, and both lab-scale studies and the industrial applications of OSN membranes are included.

### 4.1 API Concentration

The enrichment of pharmaceutical compounds, such as antibiotics, pharmaceutical intermediates and peptides, is one of the classical applications of membrane technology.^6^ The reason is the ability to concentrate APIs at room temperature instead of using distillations where a higher temperature is often required, which might cause the degradation of APIs. In a typical API concentration process using OSN, the higher MW API is retained by the membrane and the lower MW solvent passes through the membrane freely. The final product mixture of the API concentration process will be the retentate; thus, a high rejection toward the API molecules is preferred.

The concentration process can be applied for the concentration of dilute extracts from natural resources, the product recovery from fermentation processes, the recovery of high-value APIs from mother liquors, the recycling of resolving agents in chiral resolution processes etc. These applications via OSN offer benefits in both environmental and economic aspects. For example, a comparison between OSN and distillation shows that the energy consumption of OSN is 200 times lower.^53^Table 2 summarizes selected examples of API concentration by OSN membranes. Martinez et al.^54^ investigated the recovery of the API 1-(5-bromo-fur-2-il)-2-bromo-2-nitroethane (G-1, 296 g mol^–1^) from a waste ethanol stream using a commercial NF270 membrane (Dow) and two lab-made polyethersulfone (PES) membranes. A high G-1 recovery rate of 99% was achieved by using a two-stage nanofiltration system in series. DuraMem^TM^ 200 was also selected to recover active compounds hypericin from the dilute ethanolic extract since it has high rejections of above 95% toward hypericin.^55^ Shi et al.^56^ prepared a polyimide membrane for concentrating spiramycin extract after extraction from bacterial broths with butyl acetate, to replace the traditional thin-film evaporating method. The membrane showed a high spiramycin rejection of 99% and maintained long-term stability for 35 days. A novel TFC membrane with the immobilization of host–guest adamantane structure in the polyamide selective layer was designed.^57^ It demonstrated a high rejection of 99% in the long term operation of API concentration of clarithromycin/acetone. Compared to a flat sheet membrane, a hollow fiber membrane has higher packing density and self-supporting capability. Goh et al.^38^ synthesized a 100-piece hollow fiber thin-film membrane module, with P84 polyimide as the support and m-phenylenediamine (MPD)-based polyamide as the selective layer. The TFC membrane, after solvent activation by dimethylformamide (DMF), showed an enhanced acetone permeability of 24.2 L·m^–2^·h^–1^·bar^–1^ and a MWCO of 269 Da. Furthermore, its API concentration application was also demonstrated by concentrating levofloxacin (361 g mol^–1^) from 500 ppm to 20,000 ppm in acetone.

**Table 2:**
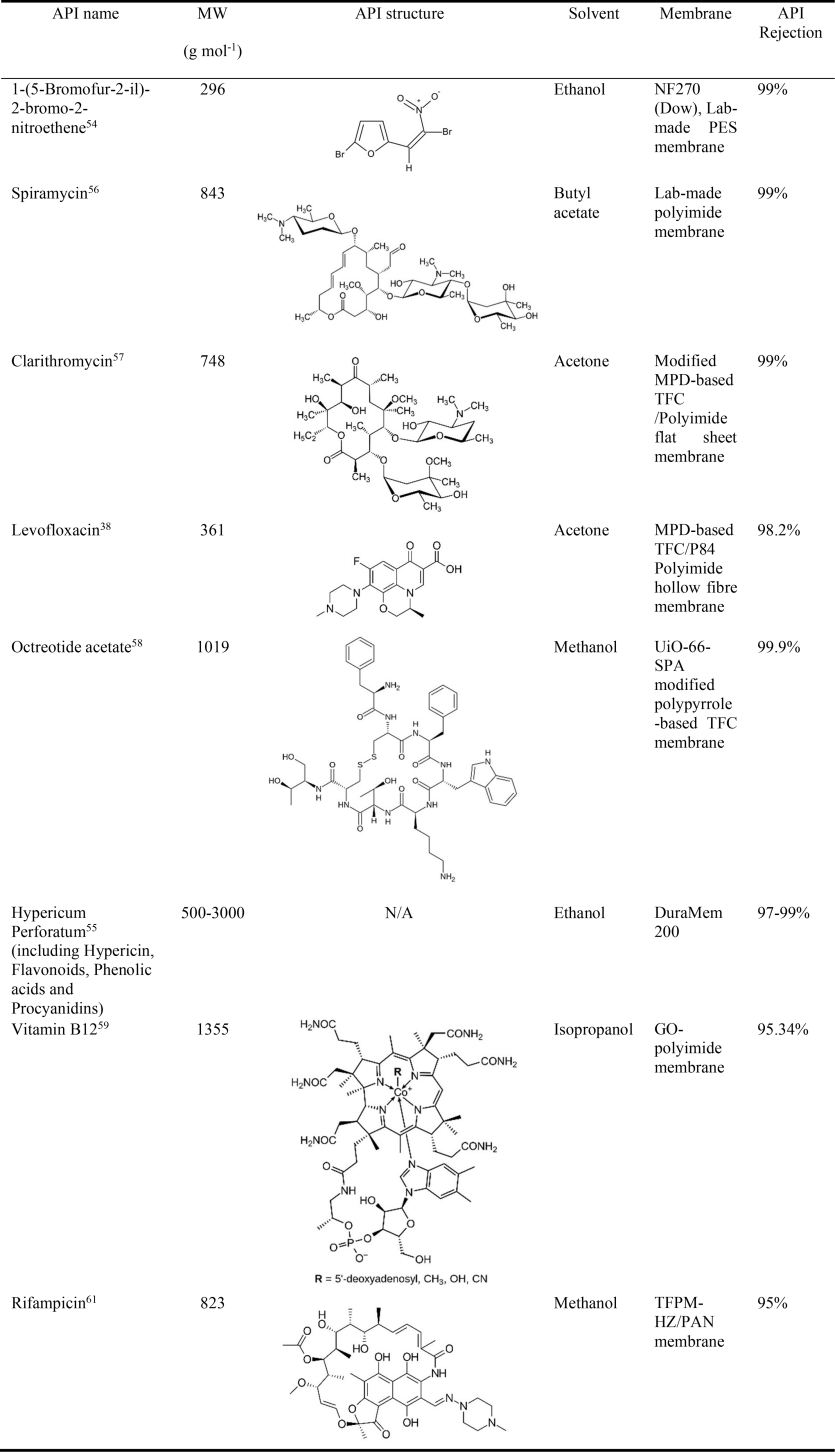
Summary of the API Concentration by OSN Membranes

Some efforts have been made to break the tradeoff between permeance and selectivity, such as the reduction of the selective layer thickness and the construction of additional solvent channels by mixing nanoparticles in the selective layer. Huang et al.^58^ added nanoparticles of poly(sodium methacrylate)-grafted UiO-66 into the polypyrrole selective layer to tailor the pore structure of the membrane. The optimized membrane showed a high rejection of 99.9% toward octreotide acetate (1079.3 g mol^–1^) and an excellent methanol permeability of 88.8 L·m^–2^·h^–1^·bar^–1^, which is about ten times higher than that of commercial polymeric OSN membranes.

**Figure 10:**
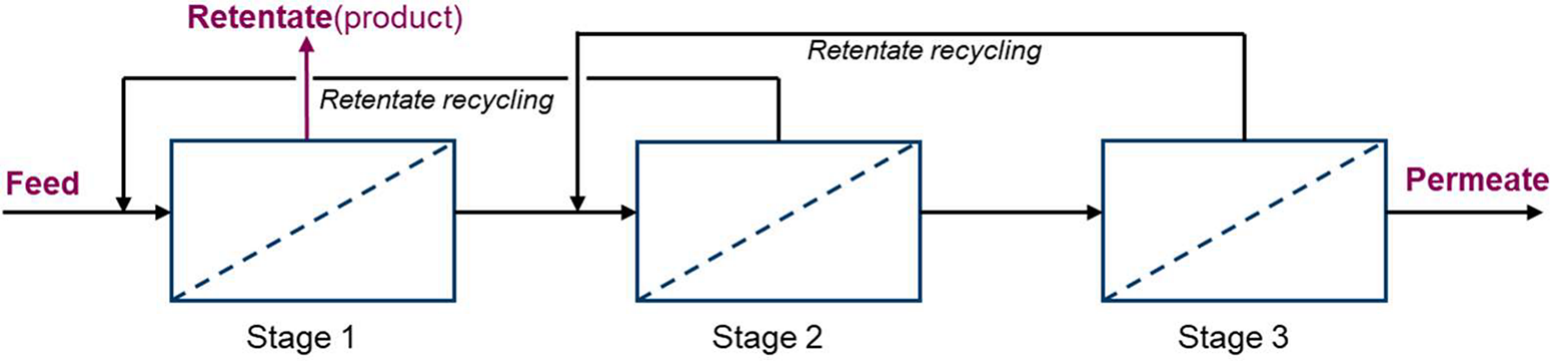
Schematic of a three-stage membrane cascade. The permeate of the first stage enters the second stage as the feed solution for further concentration. The retentate is the final product of the concentration process.

Membranes prepared with new materials, such as graphene oxide (GO)^59^ and covalent organic framework (COF),^60,61^ have also shown promising applications in API concentration. For example, a GO composite membrane was prepared on the polyimide support.^59^ It demonstrated rejection above 95% and long-term stability in the enrichment of Vitamin B from isopropanol. Due to the merits of rigid crystalline frameworks, spatially continuous channels, and hydrophobic pore chemistry, a three-dimensional COF membrane on the porous polyacrylonitrile support was specially developed for OSN applications.^61^ The membrane showed a high and stable methanol permeability of 44 L·m^–2^·h^–1^ bar^–1^ and a sharp MWCO of around 300 Da. The thin membrane also demonstrated a high rejection toward APIs such as curcumin (91%), tetracycline (100%), rifampicin (95%), and vitamin B12 (96%). Furthermore, benefiting from its uniform crystalline nature, the membrane exhibited record stability against solvent swelling and physical aging in the long term operation for 1000 h. Additionally, a multistage membrane cascade has been proposed to tackle the problem of insufficient rejection.^23,62^ Compared to a single-stage rejection of 55%, a three-stage membrane cascade (Figure 10) can achieve an overall rejection of 80%.^23^

Besides academic progress, VITO demonstrated a successful API recovery from a methanol-based distillation residue at Sitetech-DSM in Venlo.^63,64^ A GMP-compliant mobile OSN pilot plant,^65^ which can be equipped both with ceramic (∼0.7 m^2^) and polymeric membranes (∼5 m^2^), has recovered > 10 tons of API over a period of 6 months.

### 4.2 API Purification

Purification is a crucial step in API manufacturing, aiding in eliminating impurities that affect safety and drug efficacy. Impurities can be classified into organic impurities, inorganic impurities and residual solvents.^66^ These impurities can arise due to side reactions in synthetic/manufacturing processes, degradation, storage conditions, leaching/extracting from containers, excipients and contamination.^67^ Pharmaceutical manufacturers must eliminate impurities to the greatest extent to protect patients and meet the strict requirements from regulatory authorities. Traditional API purification methods to remove impurities include crystallization, distillation, and chromatography. However, distillation often needs elevated temperatures and phase change, which brings high energy costs and may induce the degradation of products.^6^ The industry predominant batchwise crystallization has scale-up problems and batch to batch variability.^68^ Chromatography significantly increases the process mass intensity (PMI), defined as the total mass of materials used to produce a given mass of product, mainly due to the use of large quantities of solvent.^69^ Compared to those conventional separation processes, OSN membrane separation has significant advantages of low energy consumption, carbon and space intensity, continuous operation mode and straightforward scale-up.^6^

**Figure 11:**
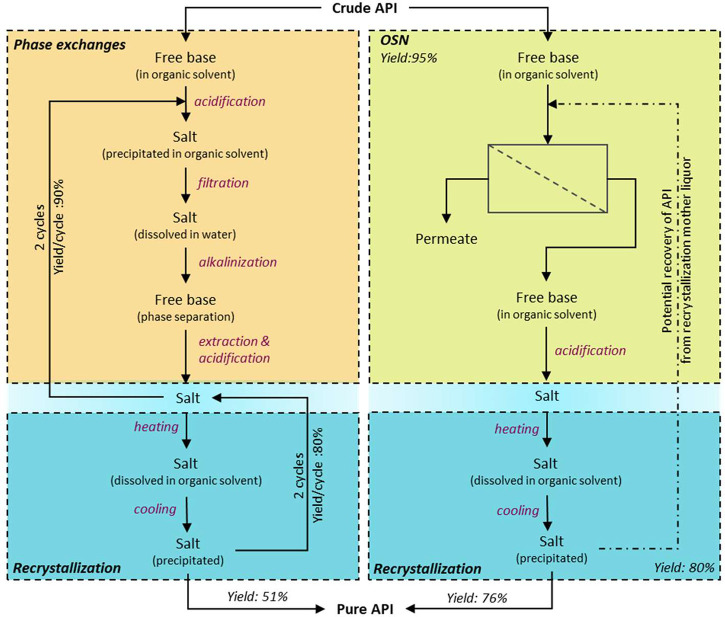
A schematic of API purification by a conventional and OSN-based process: the conventional process includes a sequence of stages of solvent exchanges followed by recrystallization, while the OSN-based process simplifies the process by replacing the solvent exchanges with a membrane unit. Reproduced with permission from ref (72). Copyright 2011 Elsevier.

**Figure 12:**
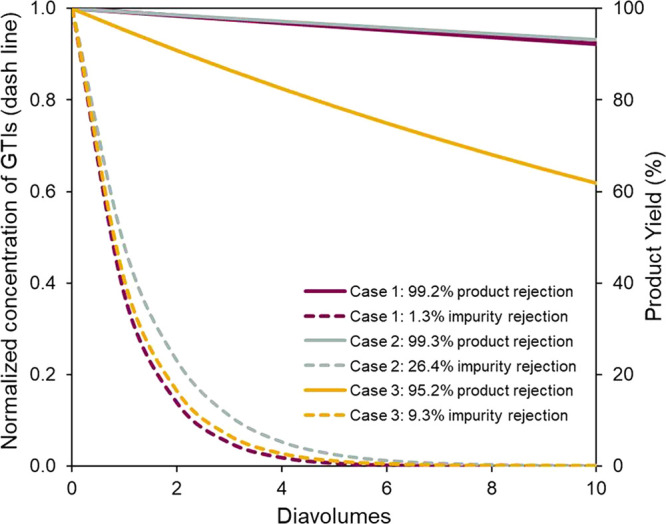
Three case studies to remove the GTIs from API via constant volume diafiltration. Case 1 presents an easy case with 99.2% rejection toward API and 1.3% rejection toward GTIs. Case 2 shows a high rejection of 99.3% toward API but a slightly high rejection of 26.4% toward GTIs, requiring more solvents (diafiltration cycles) to wash out the impurities. Case 3 demonstrates a slightly low rejection toward API (95.2%), leading to a high API loss during the diafiltration process to wash out the impurities. Reproduced with permission from ref (72). Copyright 2011 Elsevier.

Genotoxic impurities (GTIs), which can damage DNA, leading to genetic mutations and potentially cause cancer, are one of the representative API impurities and have received increasing regulatory and industry attention.^70,71^ The MWs of GTIs are normally in a range of 55–225 Da^72^ and much smaller than that of many APIs, which is beneficial for a good separation by OSN. Also, compared to the conventional API purification process, the OSN-based process is relatively simple (Figure 11).^72^ The OSN-based process runs in a diafiltration mode: fresh solvent is added to compensate for the solution leaving the system, while the smaller GTIs are washed through the membrane and the bigger API molecules are retained. Thus, an ideal membrane should have both a low rejection of GTIs and a high rejection of API. Three case studies were presented by Székely et al. to give guidance for API/GTI separation (Figure 12).^72^ The first case shows an ideal case of OSN for API/GTI separation, where the membrane has a near 100% rejection to API and zero rejection to GTIs due to the large MW difference between API and GTIs. The second case with a higher rejection toward GTIs was illustrated. OSN is still feasible; however, more volumes of fresh solvent are needed to purify the impurities to an acceptable low level. The third case showed a slightly lower rejection toward API (95%), which will result in a huge API loss of 40% at 10 diavolumes, where diavolume represents the total volume of the added solvent relative to the initial system volume).

**Figure 13:**
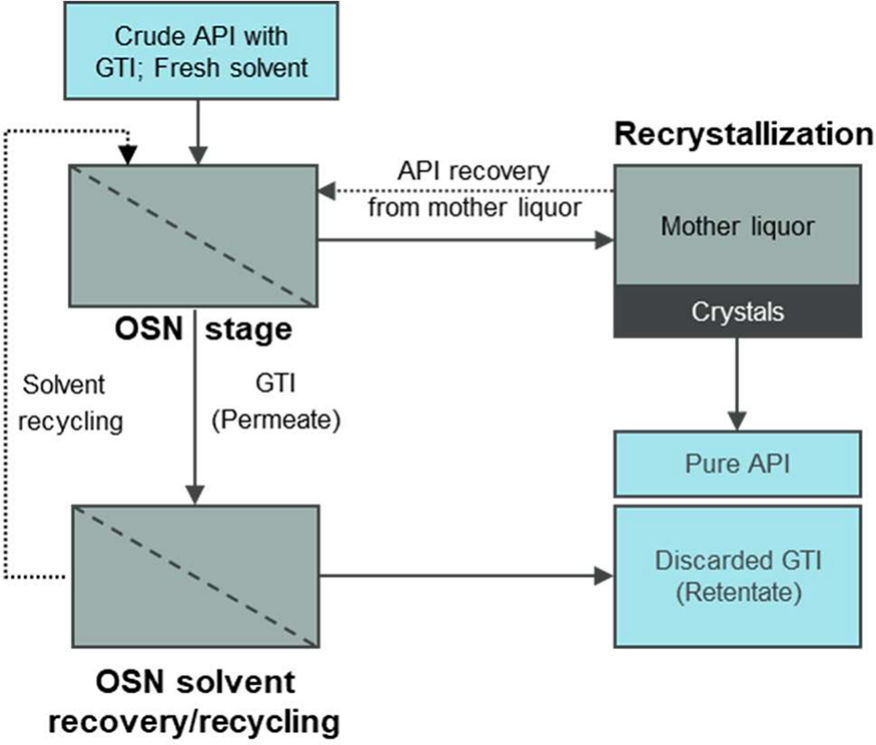
A schematic flowchart of the OSN-based API purification process for GTIs removal, with the incorporation of solvent recovery/recycling. Reproduced from ref (71). Copyright 2015 American Chemical Society.

The performance efficiency and sustainable impact of the removal of GTIs by OSN have been compared with conventional recrystallization and flash chromatography.^73^ The conventional methods achieved the limits of GTIs imposed by regulatory agencies at the expense of high API losses. In contrast, the OSN process had the least API loss; however, its high performance was achieved at the expense of high solvent usage. Therefore, the implementation of a solvent recovery unit is crucial to the sustainability of OSN diafiltration.^69,74^Figure 13 presents a schematic of GTI removal by OSN with the potential use of OSN membranes for solvent recovery/recycling.^71^ Without the addition of fresh solvent to the system, the solvent recovered from the solvent recovery stage is recycled to the API purification stage. The feasibility of combining OSN-based solvent recovery with the purification stage has been demonstrated by Sereewatthanawut et al. using DuraMem 300 membrane, where the solvent usage has been reduced by more than 90% in the separation of oligomer impurities.^69^ Membranes with lower MWCO (close to 100 g mol^–1^), which can fully reject small molecules but allow the pure solvent to pass through, are critical for the wide use of solvent recovery.^75,76^ An improved in situ solvent recovery unit using tight OSN membranes (DuraMem 150) has shown the possibility of adopting a solvent recovery unit down to 100 g mol^–1^ and reducing the solvent consumption to nearly zero. Also, compared with the adsorptive and distillation-based solvent recovery, OSN-based solvent recovery has advantages in low solid waste generation and low carbon footprint.^76^ More solvent recovery applications of OSN are presented in Section 4.6.

**Figure 14:**
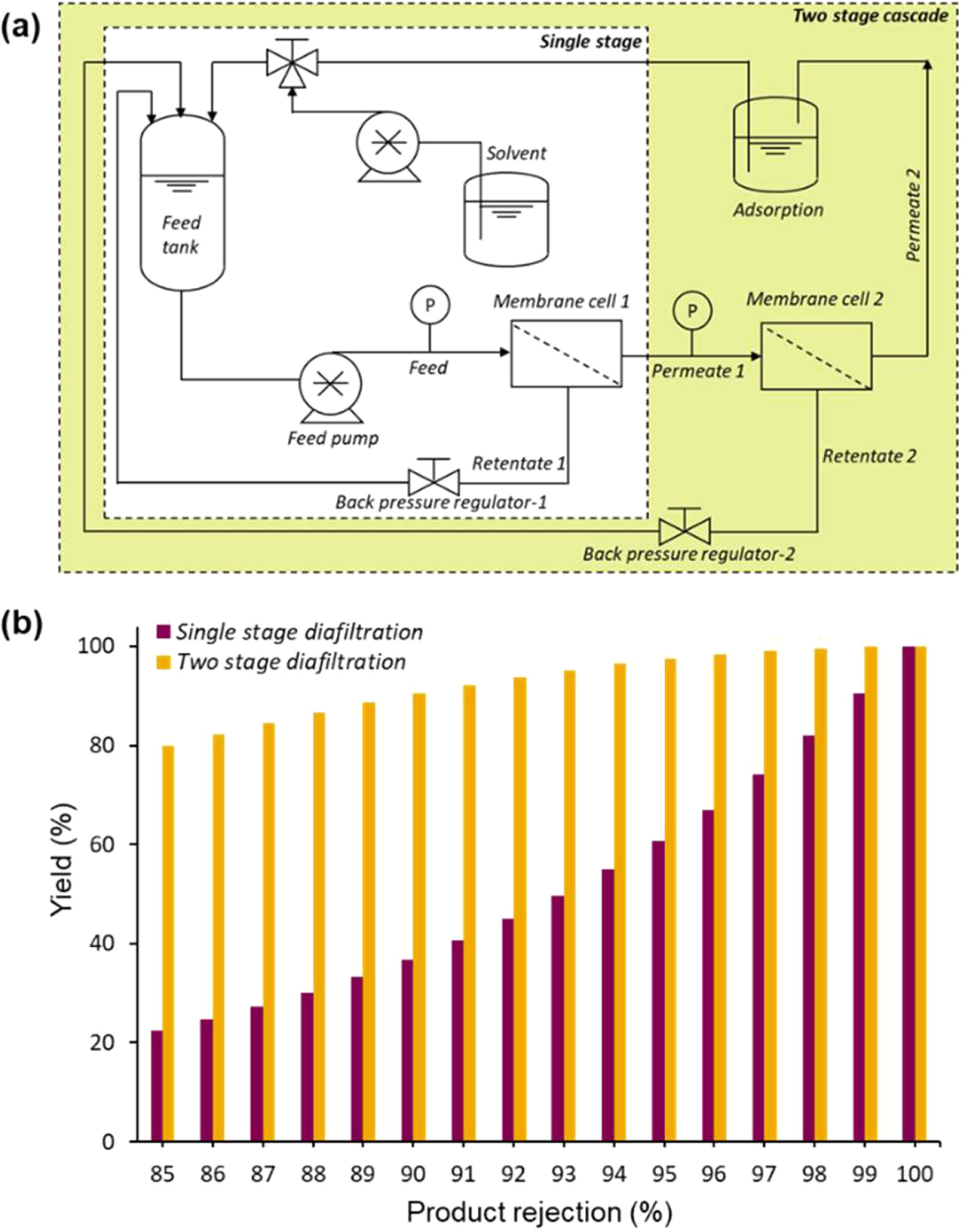
(a) Schematic of two-stage membrane cascade: the permeate of the first stage is directly connected to the feed of the second stage and both retentate of the first and second stages are recycled back to the feed tank. (b) Predicted product yield after 10 diavolumes for different product rejections. The two-stage diafiltration significantly improves the yield. Reproduced with permission from ref (78). Copyright 2014 The Royal Society of Chemistry.

Besides the high solvent consumption, another crucial limitation of OSN in API purification is the low product yield due to insufficient rejection of the API.^6^ The product yield (or the overall rejection) can be improved by employing a membrane cascade with two or more stages (Figure 14b).^62,77−79^ Kim et al.^78^ investigated the removal of two GTIs (4-dimethylaminopyridine and ethyl tosylate) from API (roxythromycin). However, the 94% rejection of the API is insufficient to achieve a high yield after purification by diafiltration. By applying a two-stage cascade configuration (Figure 14a), the API yield increased from 58% (for single-stage diafiltration) to 95% (for two-stage diafiltration) without compromising the final purity of less than 5 ppm GTI. The calculation (Figure 14b) further confirmed the two-stage membrane cascade can significantly improve the yield of API relative to a single diafiltration stage. By using a two-stage membrane cascade, a product rejection of 90% is enough to obtain a high product yield (>90%). Vanneste et al.^80^ also demonstrated a challenging impurity removal of ethylene bromide (MW 188 g mol^–1^) from an API intermediate 1-(2-Bromoethyl)-4-ethyl-1,4-dihydro-5H-tetrazol-5-one (MW 221 g mol^–1^). Since the MW difference is only 33 g mol^–1^, a three-stage membrane cascade was proposed to improve the yield of the product while maintaining a purity requirement of 90%. The experimental results showed that the three-stage membrane cascade significantly increased the purity of the API intermediate from 26% to the required 90%. Furthermore, the cascade modeling improved the yield of the API intermediate from 35.5% to 84.3%.

Hybrid processes, which combine the advantages of OSN and adsorption, were also proposed to increase the API yield during GTI removal. Székely et al.^81^ developed a hybrid process which combined the OSN with the molecular imprinting scavenger to remove GTIs. OSN was first applied to remove high concentration GTIs (1000 ppm), which run at a low diavolume of 3 to avoid excessive loss of API. The purified retentate with a low concentration of GTIs (100 ppm) was further adsorbed by the scavenger, which is more efficient at a low concentration range. Consequently, the system achieved a low API loss of 3% and an ultralow concentration of GTIs (2 ppm). A similar hybrid process combining OSN and PBI adsorbers was demonstrated by Ferreira et al.,^82^ where the permeate of the OSN unit, enriched by a distillation unit, was further connected to an adsorption unit. The ratio of GTI/API was decreased by removing GTIs via adsorption and the stream was further recirculated back to the feed side of OSN to minimize the API loss. The experimental results confirmed that the hybrid process can significantly reduce the API loss from 24.76% in OSN to 9.76% in a hybrid process.

**Figure 15:**
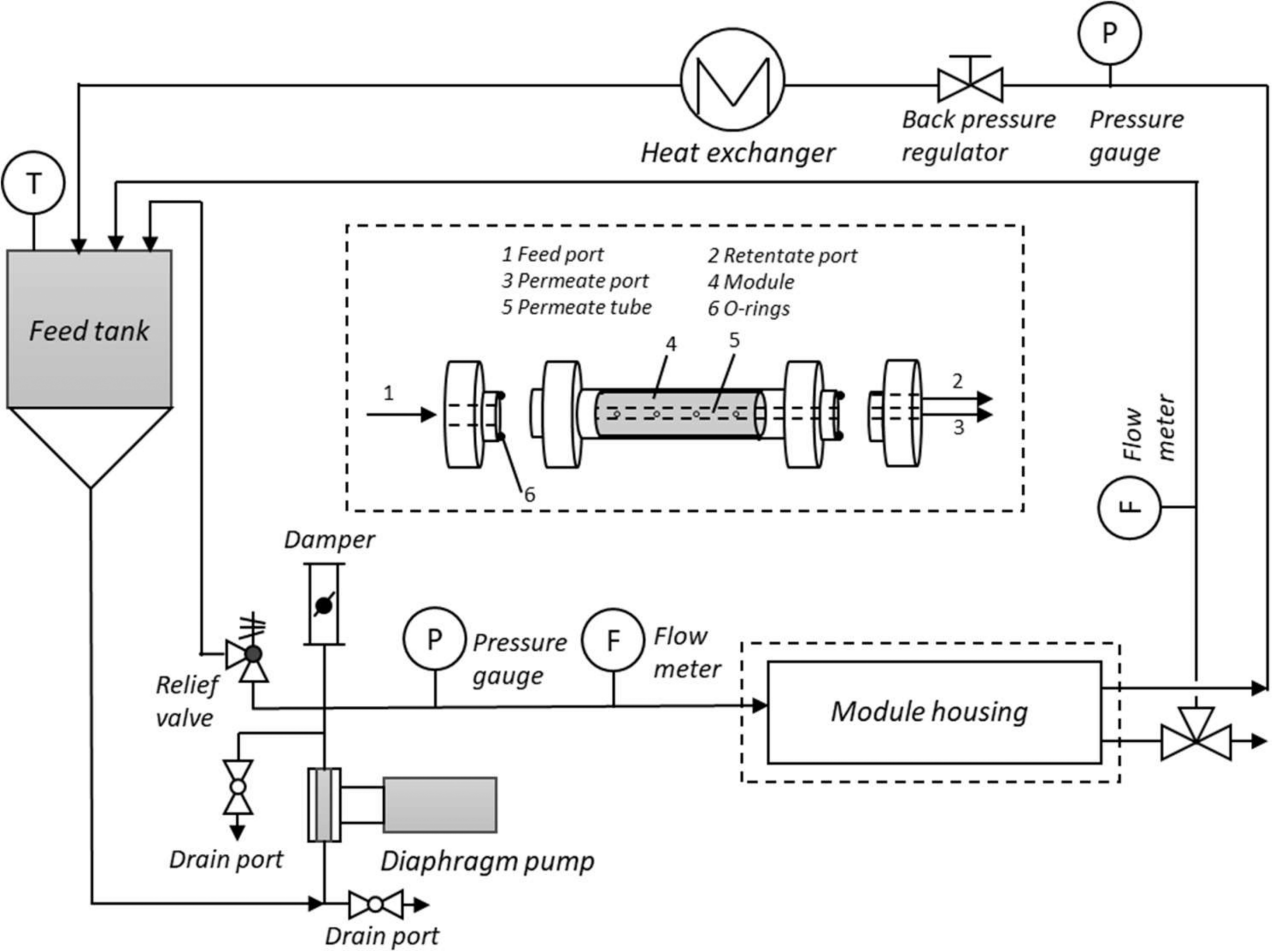
A pilot plant filtration unit for API purification with a 5 L capacity feed vessel and 1.8 in. × 12 in. DuraMem spiral-wound modules. Reproduced from ref (69). Copyright 2010 American Chemical Society.

**Scheme 1:**
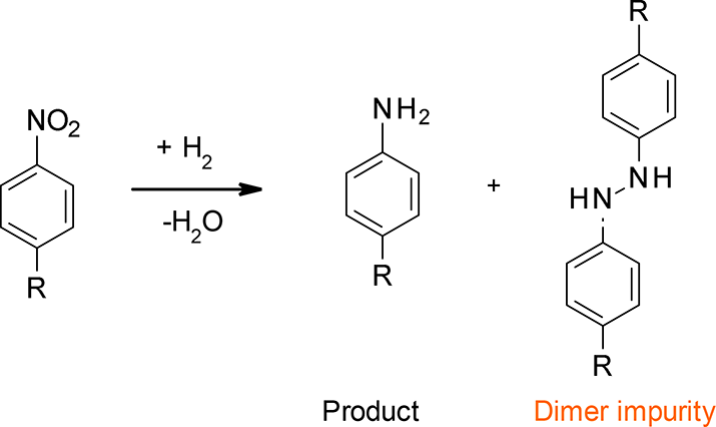
Primary Product and Dimer Impurities of the Reduction of an Aromatic Nitro Group to an Amine

Oligomeric impurities (such as dimers and trimers) are also common in API manufacturing. Those impurities with properties similar to the API are normally rather difficult to separate by standard separation methods, including chromatography and crystallization.^69^ As an alternative method, OSN shows great potential to separate them from API by allowing the API to permeate through and retaining its dimers or trimers since the MW of oligomeric impurities is two times or more than that of the API. An actual case study at Janssen Pharmaceuticals NV has demonstrated the separation of an API intermediate (MW, 675 g mol^–1^) and its oligomeric impurities (MW > 1000 g mol^–1^) by OSN.^69^ Compared to crystallization and charcoal treatment, OSN showed better efficiency to remove the oligomeric impurities with less than 1% API loss.^69^ In the pilot plant, the Evonik DuraMem^TM^ spiral-wound modules achieved 99.7% final purity and 90% API recovery and maintained a consistent separation performance for up to 120 days in THF (Figure 15). Ormerod et al.^83^ found the addition of a strong acid can change the rejection profile of the API intermediate in mixed THF/water solvent but does not affect the high rejection of its dimeric impurities (Scheme 1). It is believed the complete protonation of the amine groups increases the hydrophilicity of the API intermediate, contributing to its fast water-assisted transport through the membrane and low rejection. However, the protonation of the larger dimer does not affect its hydrophilicity to the same extent. As a result, the rejection difference between the API intermediate and its dimer increases with the pH adjustment by mineral acids, leading to a good separation.

### 4.3 Homogenous Catalyst Recovery/Recycling

In the synthesis of API via a series of intermediate steps and chemical reactions, homogeneous organometallic catalysts are often used due to their high selectivity and rate enhancement.^84^ For example, homogeneous palladium-catalyzed couplings occupy 22% of all the reactions in the pharmaceutical industry.^85^ However, compared to heterogeneous catalysts, homogeneous catalysts have a major disadvantage of problematic separation from the reaction mixture.^84^ For catalyst recovery/recycling, distillation can be used via the collection of the API or intermediate as a distillate and leaving the nonvolatile catalysts in the distillation residue.^86^ However, this normally requires elevated temperatures that may decompose the API (or intermediate) and the expensive homogeneous catalysts, since many of them are thermally sensitive.^84^ Chromatography is straightforward but limited to the laboratory scale and its high solvent consumption does not meet the criterion of sustainable manufacturing.^87^ Extraction requires the catalyst to have a significantly different solubility from the product, leading to one predominantly present in the aqueous phase. However, since most catalysts are not water soluble, they cannot be removed by extraction if the product is not water soluble. Extra steps, such as the addition of a chelating reagent, are required to facilitate the transfer of the catalyst into aqueous phase.^88^ Adsorption is a widely used technique; however, the adsorbent may unselectively adsorb the product, resulting in huge API product loss. Also, it might leak new impurities which contaminate the final product, requiring further purification steps.^88^ Compared with other catalyst recovery techniques, OSN can selectively separate the catalyst from the product without phase transition and biphasic operation, which makes the recovery and reuse of homogeneous catalysts easier and greener.^87,89^ A technological evaluation showed that significant energy and cost savings of up to 85% and 75%, respectively, can be achieved by OSN, compared to that of distillation.^90^

Catalyst recovery/recycling via OSN is also a typical purification process (Figure 3c) to separate the catalyst from the product. Similarly, the greater the difference in their MWs, the easier the separation.^91^ Furthermore, the overall rejection of the catalyst should be as high as possible (99.9%) to prevent catalyst leaching, thereby avoiding negative effects by the catalyst in the subsequent steps.^87,92^ From a material research perspective, those requirements can be met by either improving the selectivity of the membrane itself or modifying the catalyst to be more highly rejected. Many modification methods have been explored to enlarge the size of the catalyst by anchoring catalysts to soluble supports, such as dendrimers and soluble polymers.^84^ The first enlarged catalyst to be recovered by OSN was demonstrated by Giffels et al.^93^ and Felder et al.^94^ They applied a Koch MPF-50 membrane to recover the polymer-enlarged chiral oxazaborolidine catalysts in methanol and a catalyst rejection of 98% was finally achieved.

The separation of homogeneous catalyst from the reaction mixture by OSN can be run at off-line or online mode.^95,96^ In the off-line mode, OSN serves as a post-treatment step. The reaction is conducted separately in a batch reactor. Once the reaction completes, the reaction mixture is transferred to the OSN unit for further purification. The catalyst retained by the membrane returns to the reactor to start another cycle of reactions. The online operation runs in semicontinuous or continuous mode, where the reaction and OSN separation occur simultaneously. During the reaction, the OSN membrane separates the catalyst from the reaction medium and the catalyst is pumped back to the reactor. Such an application requires that the membrane should be compatible with the reaction conditions (such as high temperature, pressure, and aggressive solvents). The higher requirement will obviously limit the selection of membrane materials, leading to limited online applications.

Table 3 is a summary of the application of OSN in catalyst recovery/recycling (from 2001 onward) to illustrate the research trends. Among them, the recovery/recycling of Palladium (Pd), Ruthenium (Ru) and Rhodium (Rh)-based catalysts, accounting for two-thirds of the total publications, has been extensively investigated. At least two reasons may contribute to this. On the one hand, the high price of those noble metals calls for the recovery/recycling of those catalysts (Pd: $1,426 /oz, Ru: $465/oz, Rh: $6,470/oz in June 2023^97^). On the other hand, the possible toxicity of noble metals requires the removal of residual metals from products to meet the pharmaceutical requirements set by regulatory authorities. For example, the permitted concentrations of the elemental impurities of Rd, Ru or Rh are less than 10 μg/g in oral formulations and less than 1 μg/g in parenterally administered formulations.^92^ Here, we would like to focus mainly on the recovery of noble metal catalysts using OSN.

**Table 3:** Summary of the Applications of OSN in Catalyst Recovery/Recycling (from 2001 to 2023)

Catalyst	MW (g mol^–1^)	Membrane	Solvent	Rejection	Reference
**Palladium-based catalysts**					
Pd(OAc)_2_(PPh_3_)_2_+P(o-tolyl)_3_	749	Polyimide	Ethyl acetate/ acetone, Methyl tert-butyl ether, THF	90%, 96%, 96%	Nair et al.^98^
Pd-phosphine, Pd-imidazolylidene, Pd-quat, Pd(II) acetate+[PPh_4_]Br	643–856	Starmem 122, MPF-60	THF/water, Acetonitrile	92–96%	Nair et al.^99^
Pd(OAc)_2_ + (PPh_3_)_2_ organocatalyst	749	Starmem 122	Ethyl acetate/acetone	96%	Nair et al.^100^
Polymer supported Pd(PhCN)_2_Cl_2_ and Pd(OAc)_2_	-	PDMS/PAN	Toluene, NMP	99.95%	Datta et al.^101^
Multi(NCN-Pd and/or -Pt) pincer complexes	>700	Koch MPF-50, MPF-60	CH_2_Cl_2_	-	Dijkstra et al.^102^
Pd_2_(dba)_3_-CH_3_ + PPh_3_	1035	Starmem 122	Ethyl acetate /CyPhos101	>95%	Wong et al.^103^
Pd(OAc)_2_ + PPh_3_, Pd_2_(dba)_3_-CH_3_ + PPh_3_	224.5/1035.1	Starmem 122	Toluene/Ethyl acetate	-	Pink et al.^104^
“Click” dendritic phosphines and (PdOAc)_2_	>1600	Inopor TiO2 0.9 nm	THF/water	-	Janssen et al.^105^
Nolan-type (NHC)Pd(allyl)Cl complexes	391–1081	PDMS/PAN	Isopropanol	97–99%	Schoeps et al.^106^
PCP pincer ligand with [(allyl)PdCl]_2_ type catalyst	1223–1910	Koch MPF-50	THF, CH_2_Cl_2_	70–99.4%	Ronde et al.^107^
[Pd^0^(PPh_3_)OAc]	690	DuraMem	Acetone	100%	Tsoukala et al.^108^
Pd(OAc)_2_ + bis(diphenylphosphino)propane	225 + 412	PEEK, APTS cross-linked polyimide, DuraMem 300	DMF	93%	Peeva et al.^109^
Pd-NHC complexes CX-31/Peppsi-Ipr	647.63/ 679.46	1 nm C5 TiO_2_/0.9 nm C_8_H_4_F_13_-TiO_2_	Ethanol	99%	Ormerod et al.^96^
Tailed Pd-NHC complexes	1379.9	1.0 nm C8 TiO_2_	Ethanol	99%	Ormerod et al.^110^
Pd(OAc)_2_ + dppBz complex	-	PuraMem S600	Toluene	99.5%	Shen et al.^111^
**Rhodium-based catalysts**					
Rh-DUPHOS	723	MPF-60	Methanol	97%	De Smet et al.^112^
POSS enlarged Rh/TPP catalyst	-	Inpor 0.9nm TiO_2_	1-Octene	99.9%	Janssen et al.^113^
Rh-based hydroformylation catalyst	850	Starmem 122, 240	dodecene, octene	99%	Priske et al.^114^
HRh(CO)(PPh_3_)_3_	918.78	DuraMem 500	Ethyl acetate	95%	Shaharun et al.^115^
HRh(CO)(PPh_3_)_3_	>400	Starmem 240	Toluene	93%	Razak et al.^116^
Rh-PPh3 type catalyst	365	PuraMem 280, GMT-oNF-2	Toluene	96.7%, 90.5%	Schmidt et al.^117^
Rh(acac)(CO)_2_-TPP, Rh-Xantphos, Rh-Biphepos catalyst	258+ 262/579/787	MET-oNF2	Toluene	95%	Dreimann et al.^118^
Rh(acac)(CO)_2_-TPP, Rh-Xantphos, Rh-Biphepos	258 + 262	PolyAn POL-oNf-M1_1	Toluene	94%	Dreimann et al.^91^
	258 + 579			97%	
	258 + 787			97%	
Rh/Biphephos catalyst	1044.7	POL-oNF-M1_1	DMF	96%	Dreimann et al.^119^
HRh(CO)(PPh_3_)_3_, Co(C_5_H_7_O_2_)_3_	918.78	STARMEM 240	1-octene, 1-decene	98%	Peddie et al.^90^
Rh(acac)(CO)_2_+Biphephos	258 + 787	Sulzer’s PERVAP 4060	Toluene	88%	Lejeune et al.^95^
Rh(acac)(cod) + PPh_3_	310 + 262	DuraMem 150	n-decane, methanol	99%	Scharzec et al.^120^
Rh(acac)(cod) + sulfoxantphos	310 + 783	NanoPro S-3012/ DuraMem 150	Methanol	98.4%/96.6%	Schlüter et al.^121^
**Ruthenium-based catalysts**					
Ru-BINAP	929	MPF-60	Methanol	98%	De Smet et al.^112^
Ru cymene, P1-t-Oct	-	Starmem 122	Toluene	92%, 99.6%	Roengpithya et al.^122^
Ru-BINAP	795	Starmem 122	Methanol /CyPhos101	99.9%	Wong et al.^123^
Hoveyda II complex catalysts	627–2195	Starmem 228	Toluene, dimethyl carbonate	70–90%	Keraani et al.^124^
Ru-BINAP	795	Starmem 122	Methanol	98.8%	Nair et al.^125^
Mass-tagged Grubbs II and Grubbs–Hoveyda type complexes	1100	PDMS/PAN	Toluene	99.8%	Schoeps et al.^126^
Grubbs catalyst	794	Starmem 228	1-octene	99.4%	Van der Gryp et al.^127^
POSS enlarged Ru	-	Starmem 228, PuraMem 280	Toluene	99.8%	Peeva et al.^128^
POSS-tagged Grubbs–Hoveyda catalysts	-	Starmem 228, PuraMem 280	Toluene	100%, 98%	Kajetanowicz et al.^129^
Hoveyda–Grubbs, Umicore M	600, 949	DuraMem 200, Inopor 0.9 nm TiO_2_	CH_2_Cl_2_, acetone, toluene	99.5%	Ormerod et al.^130^
Grubbs-Hoveyda II catalyst	627–927	Starmem 122	Toluene	99.5%	Rabiller-Baudry et al.^131^ and Nasser et al.^132^
G2-PAMAM(Ru)_16_Cl_32_	-	EXP-133-LP, Solsep	DMF	99%	Guerra et al.^133^
Enlarged ruthenium-based olefin metathesis precatalysts	682–2195	Starmem 122	Toluene	98.5%	Keraani et al.^134^
BINAP-Ru(II)	794.65	P84 hollow fiber membrane-3MA, NH_2_-MWCNT/P84-hollow fiber-2MA	Methanol	95.5% /98.2%	Farahani et al.^135^
Grubbs–Hoveyda II catalyst	626.6	Starmem 122	Toluene	99.2%	Lejeune et al.^136^
**Other catalysts**					
Gold (Au) nanosols	-	PDMS	2-propanol	100%	Mertens et al.^137^
[Au(OTf)(IPr)]	-	Borsig oNF-1	THF	98.5%	Bayrakdar et al.^138^
[Au_2_(L)Cl_2_]	-	Borsig oNF-1	THF/2-MeTHF	99.2%/99.5%	Bayrakdar et al.^139^
[Pt(IPr*)(dms) Cl_2_]	1241.33	Borsig oNF-2	2-MeTHF/solvent-free	99.5%/98%	Bayrakdar et al.^140^
Polyoxometalate catalyst Q_12_[WZn_3_(ZnW_9_O_34_)_2_] (Q = [MeN(n-C_8_H_17_)_3_]^+^)	-	α-Al2O3/γ-Al2O3	Toluene	99.9%	Witte et al.^141^
Q_12_[WZn_3_(ZnW_9_O_34_)_2_] (Q = [MeN(*n*-C_8_H_17_)_3_]^+^)	9325	Ceramic γ-alumina membranes	Toluene	99.9%	Chowdhury et al.^142^
Phosphotungstic acid	2880	AMS Nanopro S-3012	Acetonitrile/water	94.60%	Vondran et al.^143^
Co-Jacobsen catalyst	700	COK M2, N30F	Diethyl ether, isopropanol	98%, 90%	Aerts et al.^144^
CuBr/PMDETA	317	Polyimide	DMF	45–52%	Cano-Odena et al.^145^
Magnesium triflate	322.44	DuraMem 300	Ethanol, ethyl acetate, and cyclohexane	98.02%	Schnoor et al.^146^
Magnesium triflate	-	DuraMem 300	Ethanol, ethyl acetate and water	98%	Schnoor et al.^147^
Porphyrin-functionalized dendrimer-based photocatalysts	600–8700	PDMS, PDMS-USY-PAN	CHCl_3_, Isopropanol	57–99%	Chavan et al.^148^
G1(DippImI)_4_	-	Ultracel (Millipore, MWCO 1 kDa)	Toluene	-	Krupková et al.^149^
Camphorsulfonamides	-	22 PBI	Isopropanol or THF	97%	Kisszékelyi et al.^150^
Tri-n-butyl-(2-hydroxyethyl) phosphonium Iodide	374	DuraMem 300	Ethanol	99%	Großeheilmann et al.^151^
Tetraoctylammonium bromide	546	Starmem 122	Toluene	99%	Luthra et al.^152^
TOABr phase transfer catalyst	546	Starmem 122	Toluene	99%	Nair et al.^100^
Quinine-based organocatalysts	414	DuraMem 150,200,300	THF	96.7–99.9%	Fahrenwaldt et al.^153^
Quinidine-based organocatalyst	1044–1332	DuraMem 300, DuraMem 500	THF	100%	Siew et al.^154^
Quinine-based organocatalysts	506.4	DuraMem 200	Ethanol	99%	Großeheilmann et al.^155^

#### 4.3.1 Pd-Based Homogeneous Catalysts

**Scheme 2:**
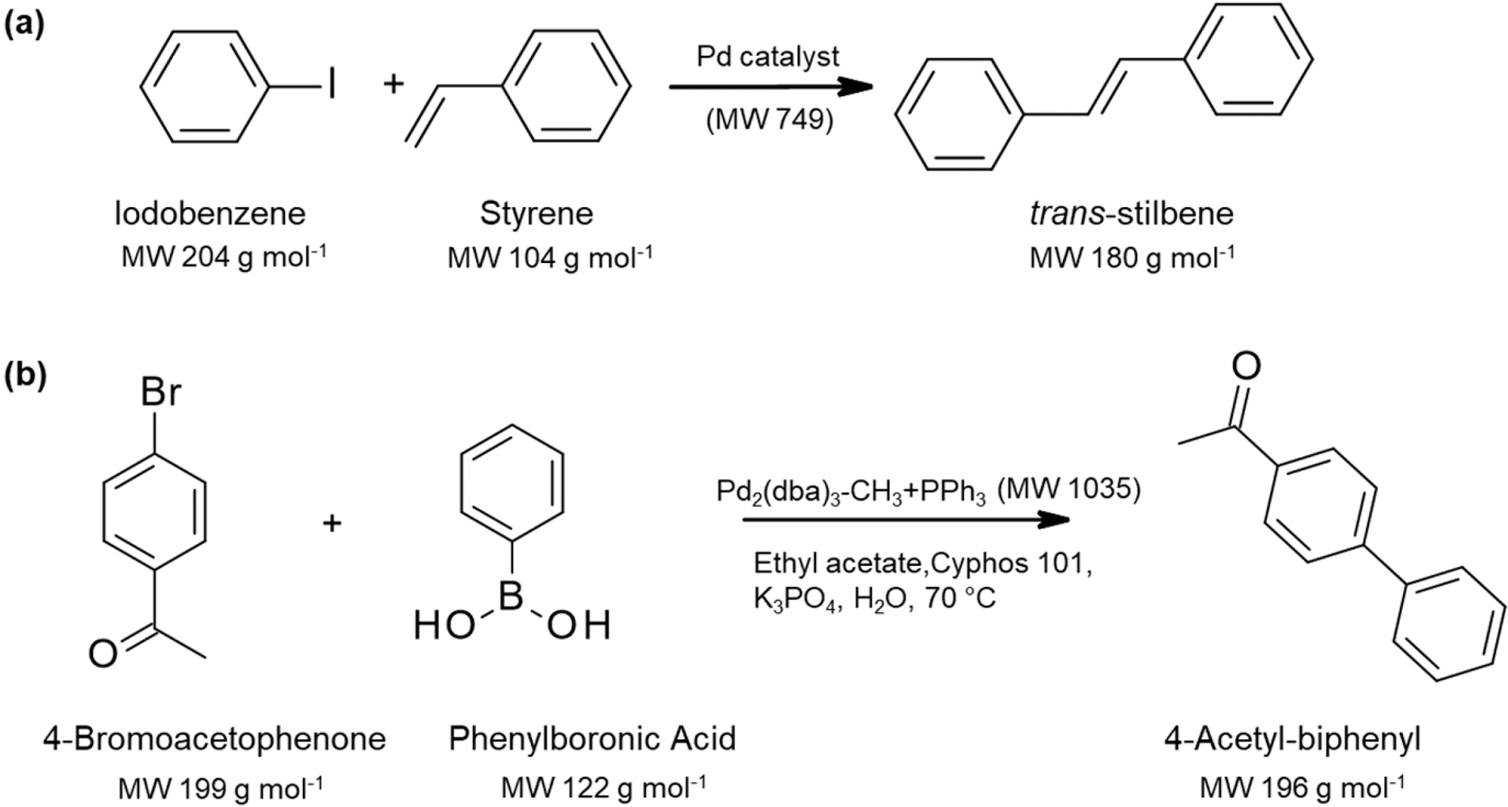
(a) Model Heck Coupling Reaction to Form Trans-Stilbene;^98−100^ (b) Model Suzuki Reaction Forming 4-Acetyl-biphenyl^103,104^

Pd-catalyzed cross-coupling reactions are versatile and efficient methods for carbon-carbon and carbon-heteroatom bond formations.^156^ Among them, Heck, Suzuki and Sonogashira couplings of aryl halides to an olefin, arylboronic acid or an alkyne, respectively, play important roles in the pharmaceutical industry.^46^ Nair et al.^98−100^ first demonstrated the recovery and reuse of Heck coupling catalysts by using the Starmem 122 and Koch MPF-60 in the solvent systems of THF/water and acetonitrile respectively (Scheme 2a). The catalyst was recycled six times at the expense of a 20% decrease in reaction rate as compared to the first run.^99^ However, the catalyst rejection is only 96% and needs further improvement. Further study by Tsoukala et al.^108^ using the second-generation Evonik DuraMem membrane showed an improved rejection of up to 100% toward Heck reaction catalysts. The membrane of Starmem 122 has also been used by Wong et al.^103^ and Pink et al.^104^ for the recovery of Pd catalysts in Suzuki reactions (Scheme 2b). The membrane can successfully retain the ionic liquid and Pd catalysts for further reuse.^103^ However, the Pd residue per unit mass of product in the permeate is unacceptable for pharmaceutical applications since the membrane only has a rejection of 95%. Ceramic membranes, which are chemically more resistant than polymeric membranes, were used to achieve high catalyst rejection. The 1 nm C5 TiO_2_ ceramic membrane showed > 99% rejection toward Suzuki catalysts (Pd-NHC complexes)^96^ in ethanol.

The ligands, used to stabilize the catalyst complex, can not only affect the yield of the reaction, but also influence the rejection of the catalysts. Typically, the Pd rejection was found to correlate well with the MW of ligands.^111^ Thus, catalysts with enlarged sizes of ligands are designed to improve the rejection of the catalyst. Datta et al.^101^ prepared a polymer-enlarged Pd catalyst (MW 5,000 Da) for Heck, Suzuki and Sonogashira couplings and a high rejection of 99.95% was achieved by using a PDMS/PAN membrane. Schoeps et al.^106^ synthesized an enlarged N-heterocyclic carbene(NHC) ligand with an MW of around 1000 Da to form complexes with a Pd catalyst. The mass-tagged Pd-NHC showed a rejection of 97% by a PDMS/PAN membrane, and the rejection can be further improved to 99.9% by a second OSN. Pd catalysts with the enlarged Princer ligands synthesized by Ronde et al.^107^ also showed improved rejection of 70–99.4%, with MWs ranging from 1223 to 1910 Da. Besides the modification of ligands to increase their size, Ormerod et al.^96^ demonstrated that the same type of ligands with differences in the ancillary ligands on the metal will also affect the overall rejection. Among the four Pd complexes with the same ligand NHC, two Umicore cross-coupling catalysts (CX31 and Peppsi-IPr) showed > 99% rejection by modified ceramic membranes in the off-line mode. However, the membrane cannot get both high catalyst rejection and high reaction yield at the same time for the online mode. They further designed a series of enlarged catalysts by modifying NHC ligands with different sizes of tails in the aryl rings of the imidazolidene structure ligands.^110^ The rejection toward the tailed Pd-NHC complexes increased with the increase of tail sizes. The highest rejection (>99%) was achieved using 1.0 nm C_8_ TiO_2_ membranes. Compared with the untailed catalysts, the tailed one also showed better resistance to the cluster formation, which contributes to high catalyst rejection and high reaction yield in the online mode.

Moving from batch to continuous processing is an important goal for the pharmaceutical industry since continuous flow chemistry can perform reactions faster and safer, with a smaller footprint, better scalability and high quality.^157^ A continuous Heck coupling reaction at elevated temperature (∼80 °C) in polar aprotic solvent (DMF) and base (concentrations > 0.9 mol L^–1^) was demonstrated by Peeva et al.^109^ The reaction and separation were performed in a single reactor/membrane separator cell assembly with an optimized PEEK membrane inside. The unit ran continuously for more than 1000 h at conversions above 85%. An overall Pd rejection of 93% was estimated. Although the Pd residue in the final product in the continuous process was 20 times lower than that of the batch process using the same catalyst loading and without membrane purification, the Pd concentration (317 mg Pd per kg of product) was too high for pharmaceutical applications. They also combined a plug flow reactor with the single reactor/membrane separator cell assembly, and high conversions of 98% and significantly lower Pd residue in the product (27 mg Pd per kg of product) were achieved.^158^

#### 4.3.2 Ru-Based Homogeneous Catalysts

**Scheme 3:**
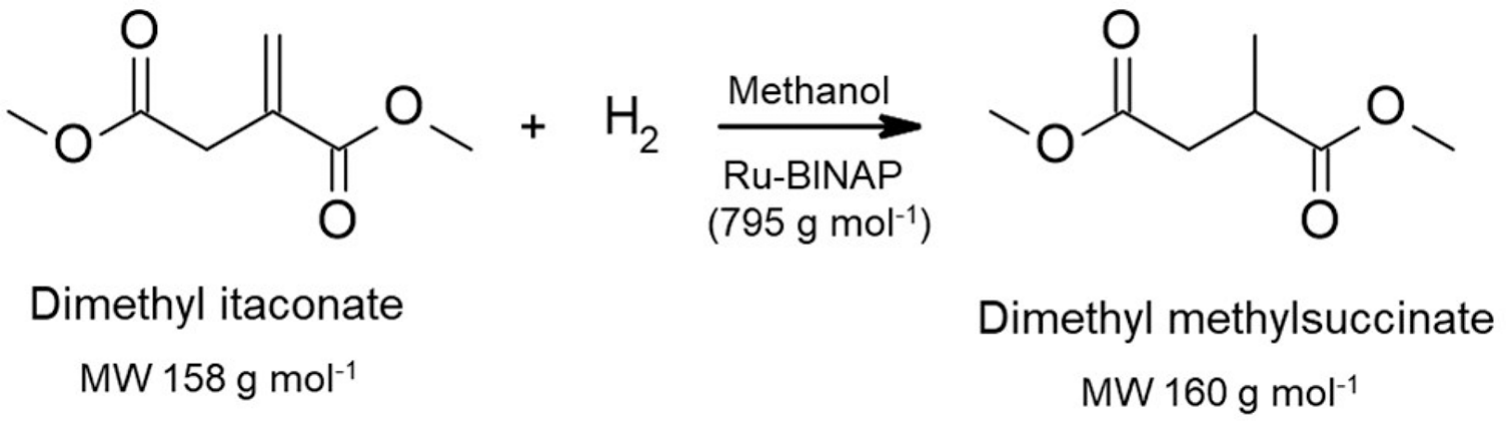
Hydrogeneration of Dimethyl Itaconate by Ru-BINAP^112,123,125,135^

The recovery/recycling of Ru-based catalysts has been developed in asymmetric hydrogenation^112,123,125,135^ and olefin metathesis.^124,126−131,134,136^ In 2001, De Smet et al.^112^ first demonstrated the recovery of Ru-BINAP catalysts in continuous enantioselective hydrogenation of the dimethyl itaconate (DMI) process (Scheme 3). Using the Koch MPF-60 membrane, the continuous process in methanol achieved a catalyst rejection of 98%. The hydrogenation catalyst also maintained a constant activity after 10 cycles. Wong et al. further reported that the enantioselectivity and the stability of the hydrogenation catalyst (Ru-BINAP) could be enhanced by ionic liquid trihexyl(tetradecyl)phosphonium chloride (CyPhos101).^123^ Moreover, a Starmem 122 membrane was applied to recover the Ru-BINAP catalyst and CyPhos101, with a high rejection of 99.9% and 98.1%, respectively. The effect of catalyst loading on the reaction conversion and enantiomer excess was investigated by Nair et al.^125^ A dilute substrate (0.8 wt.% DMI in methanol) was selected in the batch-operated OSN cell. When a catalyst loading of 0.2 mol% was used, the reaction showed no decrease in the reaction conversion and enantiomeric excess in 14 successive reactions. When the catalyst loading further decreased to 0.014 mol%, a rapid reaction rate decline during the second cycle was observed. Thus, 20% of the initial mass of the catalyst was added in each cycle to compensate for catalyst degradation and filtration loss and maintain the reaction conversion and enantiomer excess. Moreover, the process was successfully scaled up to an industrial substrate concentration of 20 wt.% DMI in methanol for 20 reaction cycles, and a similar performance was observed. For the same hydrogenation reaction, an amine-functionalized carbon nanotubes/P84 hollow fiber membrane was developed^135^ and showed a high rejection of 98.2% toward the Ru-BINAP catalyst with potential applications in pharmaceutical, food, and petrochemical industries.

**Scheme 4:**
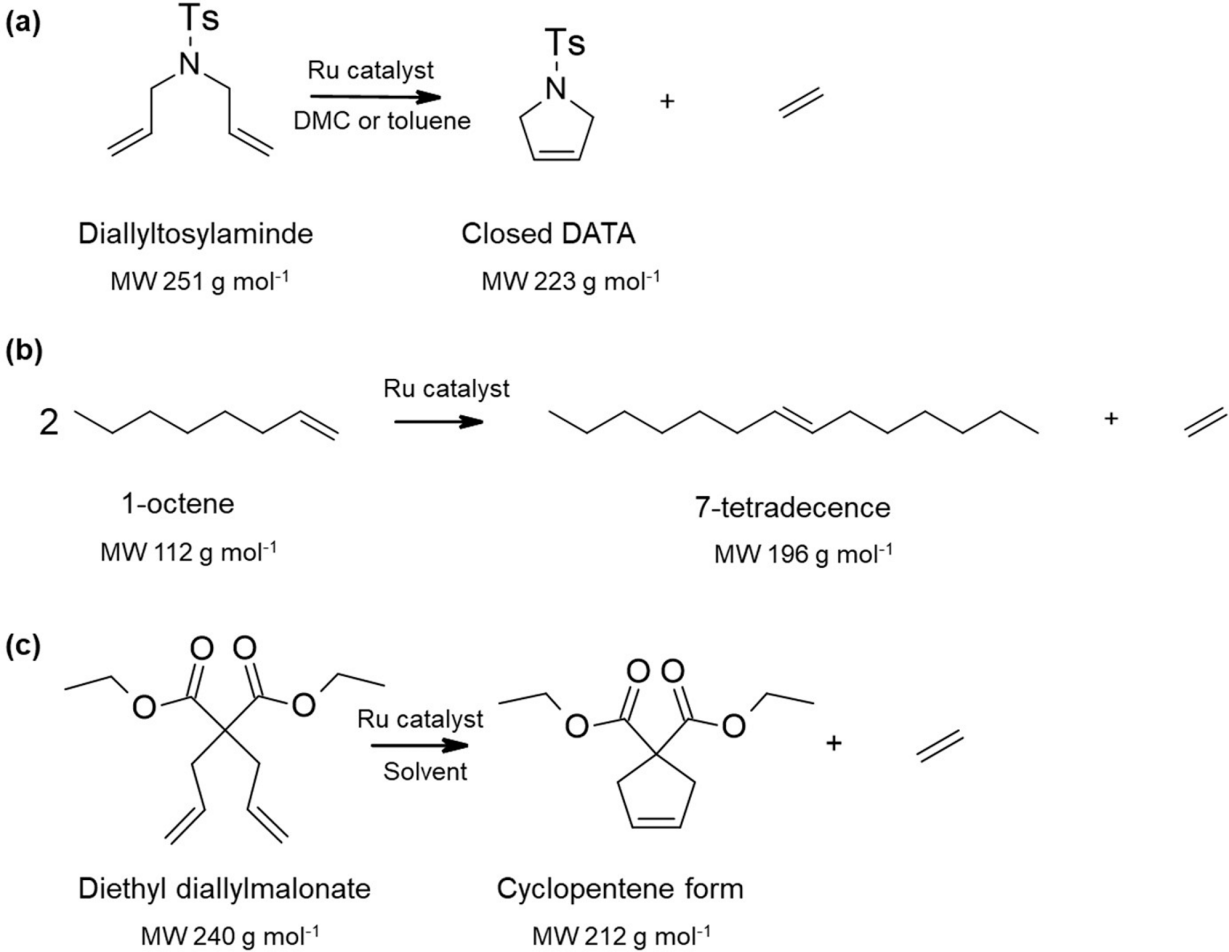
(a) Model Ring Closing Metathesis Reaction of Diallytosylamide (DATA),^124,126^ Ts = 4-Toluenesulfonyl; (b) Self-Metathesis Reaction of 1-Octene;^127^ (c) Model Ring Closing Metathesis Reaction of Diethyl Diallylmalonate^126,128−131,134^

Metathesis reactions are one of the most important transformations in organic synthesis.^159^ The MW enlarged Ru catalyst for metathesis reaction (Scheme 4a-c) was explored to facilitate the separation by OSN.^124,126−129,134^ Keraani et al.^124^ modified the commercial Hoveyda catalysts for ring-closing metathesis of diallyltosylamide (Scheme 4a) to increase their MWs from 627 to 2195 g mol^–1^, since Starmem 228 membranes do not provide sufficient rejection. After modification, the catalyst rejection by Starmem 228 was found to increase from around 70% to 90% both in toluene and dimethyl carbonate. However, the catalyst showed decreased performance after the third cycle, which could be ascribed to the deactivation of the catalyst itself and/or the catalyst loss due to insufficient catalyst rejection by OSN membranes (90%). Similar activity decline in Ru-catalyzed metathesis was also observed by Schoeps et al.^126^ and Gryp et al.^127^ Further research by Gryp et al.^127^ confirmed that the main reason for the low conversion was due to the deactivation of the Grubbs-type catalyst, rather than catalyst loss through the membrane. They designed a chelated Grubbs-type catalyst (Gr2Ph) with an excellent rejection of 99.4% by Starmem 228 membrane for the self-metathesis reaction of 1-octene (Scheme 4b). Although the catalyst loss through membrane separation is negligible, the reaction conversion using Gr2Ph dropped dramatically from 73% to 7% in the fifth reaction cycle in the coupled reaction separation and recovery process.

For the model ring closing metathesis reaction of diethyldiallyl malonate (Scheme 4c), a polyhedral oligomeric silsesquioxane (POSS) tagged Grubbs–Hoveyda catalyst was synthesized by Kajetanowicz et al.^128,129^ to improve the insufficient rejection of the original catalyst. A high rejection of 98% was achieved in toluene by using the membrane Starmem 228 and PuraMem 280. However, the catalyst stability is still a challenge for the application in continuous flow. Keraani et al.^134^ prepared five enlarged second-generation Hoveyda precatalysts by introducing structural modifications in the benzylidene ligand. The structural modification in three catalysts showed negligible effects on the catalyst efficiency with similar reaction conversion (85–86%) as the original catalyst measured after 30 min at 25 °C. By using Starmem 122 with a small MWCO of 220 Da, both the enlarged and original Hoveyda precatalysts exhibited high rejection (>98.5%) in toluene. Considering the lack of commercially available enlarged catalysts, Ormerod et al.^130^ used the commercially available Hoveyda–Grubbs and Umicore M series catalysts for the same model reaction in a continuous flow reactor, where the long-term catalyst stability is critical for catalyst recycling. Although the ceramic membrane (Inopor 0.9 nm TiO_2_) demonstrated excellent rejections toward the catalysts (>99.5%), the accumulation of the byproduct ethylene in the flow reactor was detrimental to the metathesis Ru catalyst, resulting in a much lower conversion over time. They found that the change of solvent from dichloromethane to acetone has a positive effect on the stability of the catalyst, as evident from the increased conversion of diethyldiallyl malonate from 30% to 60%. Rabiller-Baudry et al.^131^ reported the recovery of a commercially available precatalyst Grubbs–Hoveyda II by Starmem 122, which showed a high rejection both toward the catalyst (99.5%) and the product (75%) in toluene at the operating pressure of 40 bar. In the semicontinuous process, the product recovery was only 41% after two diafiltration cycles due to the high rejection of the product. It was estimated that 18 consecutive diafiltration steps were required to recover all the products. The low recovery of the product here emphasizes that the selected OSN membrane should not only have a high rejection toward the catalyst, but also a high permeation of the product. The catalyst stability was still an issue since the reaction conversion decreased from 97% to 0% after four cycles in the semicontinuous process. Similar performance was observed in the continuous mode, where less solvent but more residence time was required.

#### 4.3.3 Rh-Based Homogeneous Catalysts

The model reaction for the recovery of Rh-based catalyst mainly focuses on the hydroformylation where olefins react with synthesis gas (hydrogen and carbon monoxide) to give aldehydes. Priske et al.^114^ investigated the recovery of Rh-based catalysts in two hydroformylation processes of octene and dodecane by using two different membranes of Starmem 122 and Starmem 240. A high rejection of catalyst of >99% was finally achieved. Furthermore, they found the presence of CO was beneficial for catalyst stability by preventing the formation of inactive catalyst clusters. Schmidt et al.^117^ found that modification of solvent could enhance the membrane performance for the hydroformylation catalyst recovery. Toluene was added to the original solvent n-hexanal, which is also the product of the hydroformylation of 1-pentene. In the solvent mixture with 50 wt.% toluene, the rejection of PuraMem^TM^ 280 toward triphenylphosphine (catalyst ligands) increased significantly from 87% in n-hexanal to around 98%. However, the addition of toluene should be as little as possible as further separation of toluene from the reaction mixture would also increase the overall cost. MW enlarged catalysts were also explored to increase the catalyst rejection.^113,160^ For example, Janssen et al. designed a POSS enlarged triphenylphosphine ligand to combine with an Rh catalyst, which showed a rejection of 99.9% by a ceramic nanofiltration membrane.^113^

**Scheme 5:**
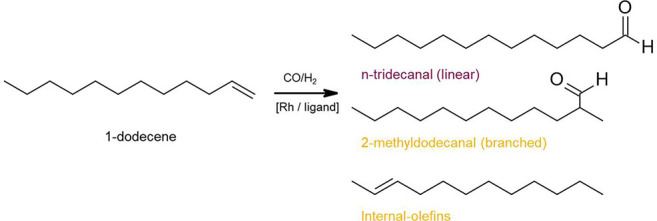
Hydroformylation of 1-Dodecene^91,118,119,161^

**Figure 16:**
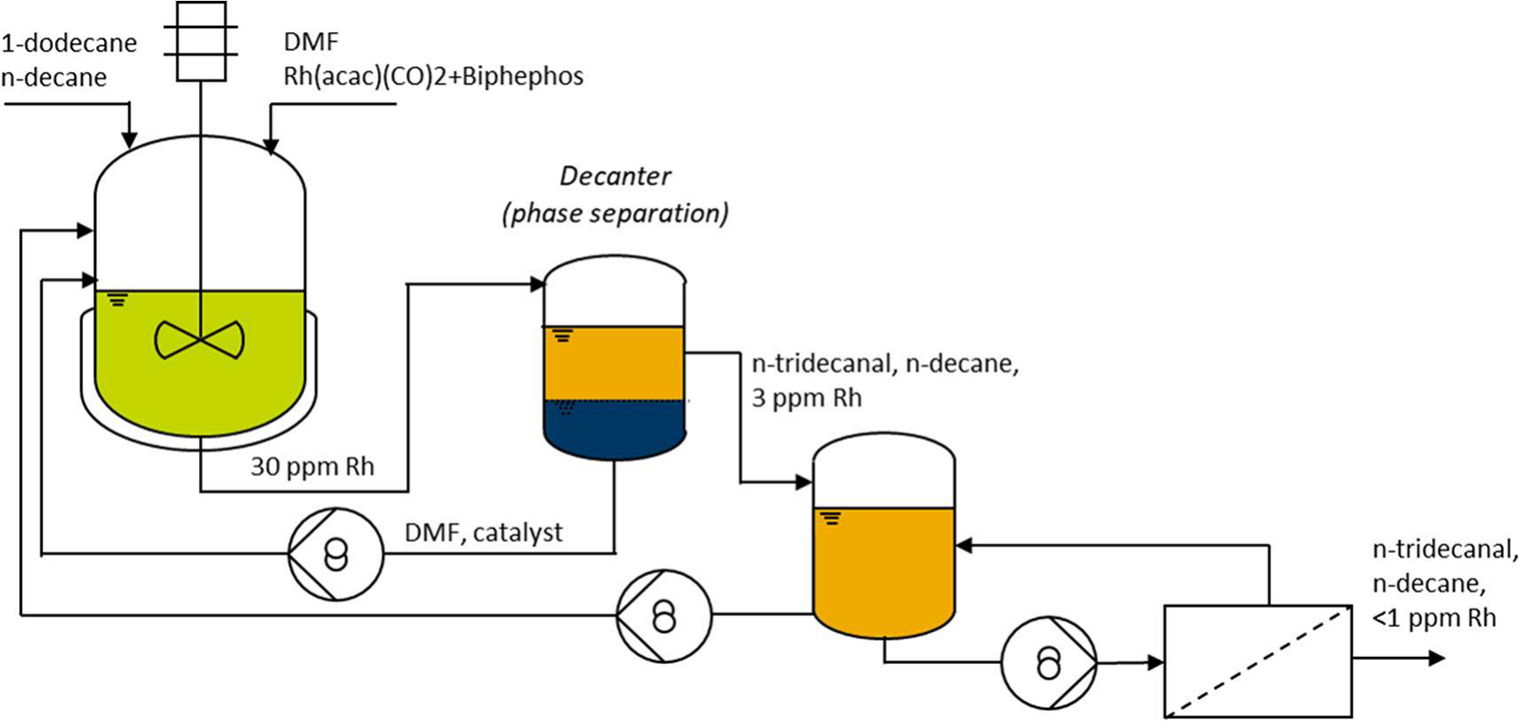
Process flowsheet for the hydroformylation of 1-dodecene using a combination of the TMS system and OSN. Reproduced from ref (119). Copyright 2017 American Chemical Society.

Dreimann et al.^91,161^ investigated the hydroformylation of 1-dodecene (Scheme 5) using an Rh catalyst with three different ligands (triphenylphosphine, Biphephos and Xantphos). A PDMS membrane was used to separate the catalyst from the product and high catalyst rejection at around 95% was achieved. To tackle the issue of insufficient catalyst recovery, they further proposed an intensified process of thermomorphic multicomponent solvent (TMS) system and OSN.^118,119^ The TMS system is based on the temperature-dependent miscibility gap of two different components.^162^ The reaction is conducted in the reactor in a single phase at elevated temperature. While the reactor is cooled below the critical solution temperature, a biphasic system consisting of a product-rich nonpolar and a catalyst-rich polar phase will be generated. However, like the conventional biphasic system, the catalyst leaking to the product phase is inevitable, necessitating further purification. As shown in Figure 16, a well-known n-decane/DMF TMS system was applied.^119^ After a preliminary separation by TMS, further recovery of the Rh catalyst from the product-rich phase was conducted by a subsequent OSN unit using a PDMS membrane. The whole continuous system, which ran for 50 h, achieved both a good product yield of 70% and a high overall catalyst recovery of 97.5%. The accumulation of byproduct can be detrimental to the TMS system if the component affects phase separation and phase distribution of the catalyst; the same group suggested OSN as a suitable method to separate a polar byproduct from the catalyst-rich polar phase.^120,121^ Scharzec et al.^120^ chose the reductive amination of n-undecanal with diethylamine and byproduct water as a case study. The byproduct water entering the catalyst rich polar phase (methanol, DMF or acetonitrile as the polar solvent) served as the feed solution for the OSN unit. In the membrane screening experiments, DuraMem^TM^ 150 showed the best performance in all polar solvents (methanol, DMF or acetonitrile) with a high rejection of more than 99% toward the catalyst ligand and a negative rejection toward the byproduct water. The continuous removal of byproduct water by membrane separation was also demonstrated by Schlüter et al.^121^ in a more complex hydroaminomethylation reaction, which combines the hydroformylation and the reductive amination in a one-pot synthesis. A continuous process using the membrane of NanoPro S-3012 was successfully operated for 75 h in a mini plant, which maintained high catalyst rejection (97–99.3%) and successfully reduced the water content from 13.8 wt.% to 4.7 wt.%.

#### 4.3.4 Other Homogeneous Catalysts

**Scheme 6:**
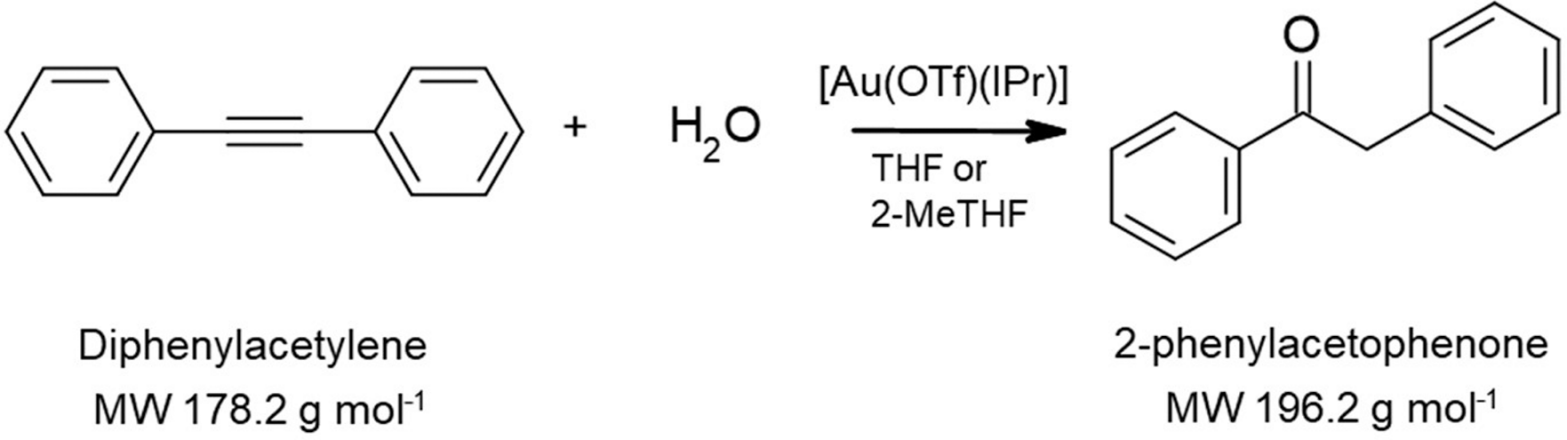
Gold-Catalyzed Hydration of Diphenylacetelyne

Besides the Pd, Rh and Ru catalysts, some other homogeneous catalysts such as gold-based,^137−139^ platinum-based,^140^ tungsten-based,^141−143^ magnesium triflate,^149,150^ Co-Jacobsen catalyst,^144^ and quinidine-based organocatalyst^153−155^ have been explored for recovery/recycling. For example, gold N-heterocyclic carbene complexes in the hydration of diphenylacetylene (Scheme 6) were first recovered by Bayrakdar et al.^138^ The Borsig oNF-1 membrane was selected due to its high rejection toward the catalyst (98.5%) and moderate rejection toward the product (53%) in the mixture of THF/water. The catalyst was successfully recovered and reused for four cycles; however, catalyst degradation was observed with the conversion decreasing from the initial 92% to 60% in the fourth cycle. The catalyst [Au(OTf)(IPr)] was finally recovered as the catalyst precursor [Au(Cl)(IPr)] in 44% yield. The latter precursor was further studied in the carboxylative cyclization of propargylamine.^139^ Although it showed better stability compared to the dinuclear catalyst [Au_2_Cl_2_(L)], only a rejection of 90% was achieved in ethanol with the Borsig oNF-1 membrane, resulting in insufficient purity of the product in the permeate.

**Scheme 7:**
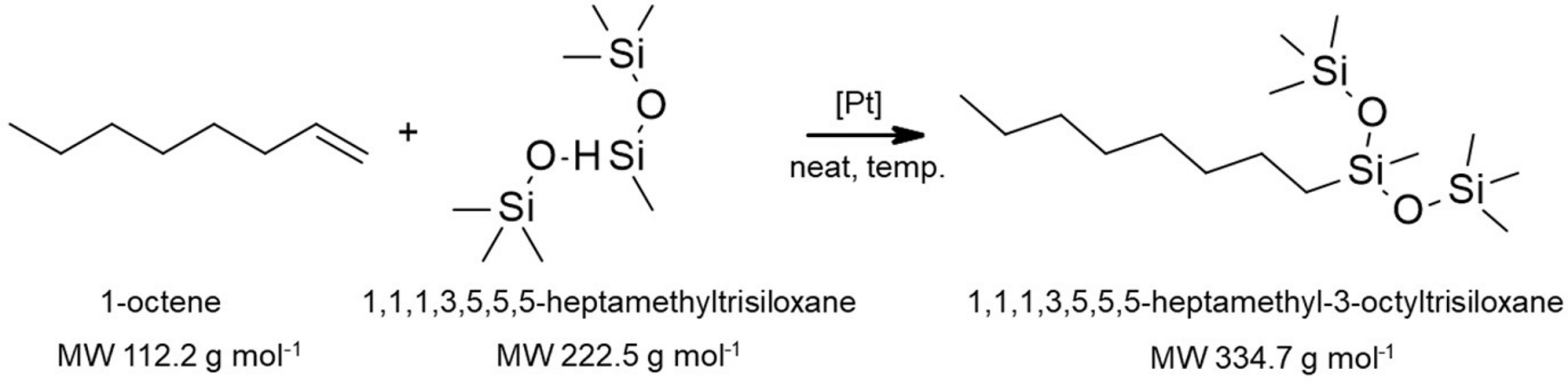
Pt Catalyzed Hydrolysation of 1-Octene

Recently, the same group^140^ first investigated the recovery of platinum catalyst [Pt(IPr*)(dms) Cl_2_] (MW 1241 g mol^–1^) in a solvent-free environment in the hydrosilylation of 1-octene (Scheme 7). In a solvent-free continuous process, the reaction starting materials were continuously pumped into the feed tank to keep the volume constant, while the product permeated through the Borsig oNF-2 membrane. After two diafiltration volumes, the results showed both a high product yield of > 97% and a high catalyst rejection of 98%-99% were achieved. Furthermore, the catalyst was recovered intact with a yield of 80% and reused for three cycles without any significant degradation.

It is worth mentioning that the aforementioned catalyst recovery was based on a separation of a larger size of the homogeneous catalyst from a smaller size product. Cano-Odena et al.^145^ reported a different case study of the copper(I)-catalyzed azide/alkyne cycloaddition reaction where the copper(I) catalyst (317 g mol^–1^) has a lower MW than the product (∼2000 Da). Consequently, an unusual strategy of OSN separation where a high rejection of the bigger product and a low rejection of the smaller catalyst is desired. An in-house polyimide membrane was selected with a product rejection of 93% and a catalyst rejection of 53%. Further 5 cycles of diafiltration experiment showed 98.8% of the initial copper removal and only 8% loss of the polymer product were observed.

### 4.4 OSN-Assisted Peptide and Oligonucleotide Synthesis

Recently, OSN-assisted organic synthesis which combines organic synthesis with OSN has been reported in the synthesis of oligonucleotides,^163,164^ peptides^165−168^ and polyethers.^169^ During the process, OSN membranes are employed to concentrate and purify the reaction products by removing undesirable byproducts/intermediates from the reaction mixture.

Therapeutic peptides, consisting of a series of well-ordered amino acids, are a unique class of pharmaceutical drugs with MWs of 500–5,000 Da.^170^ Since the introduction of the first peptide drug, insulin, in 1922, over 80 peptide drugs have been approved for clinical use and even more are in active clinical development or preclinical studies.^171^ The positive outlook of therapeutic peptides further calls for continuous innovation of synthesis and manufacturing strategies. Even though the conventional solid phase peptide synthesis (SPPS) is widely considered as the gold standard for peptide synthesis,^171^ it has drawbacks of incomplete conversions of coupling and deprotection steps and the use of excess reagents due to diffusional limitations in the solid supports.^172^ In contrast, liquid-phase peptide synthesis, using soluble support, has the potential to accomplish higher crude purity, lower reagent consumption, and greater ease of scaling.^168^ However, it is hindered by inefficient intermediate isolation methods such as precipitation and extraction.^165^ OSN, without phase change or material transfer, could be an alternative to the conventional separation method of precipitation or extraction, facilitating the automation of the process. The idea of peptide synthesis using an ultrafiltration membrane was first proposed by Bayer and Mutter in 1972.^173^ However, the incompatibility of dialysis membranes in organic solvents requires a prior step of solvent exchange with water, making this strategy complicated and impractical. No significant progress had been made in membrane-assisted peptide synthesis until the organic solvent resistant membranes reached the market.

**Figure 17:**
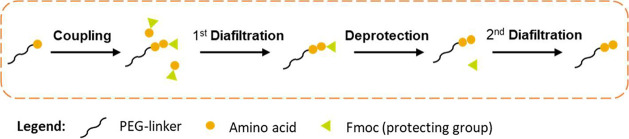
Schematic of the OSN-assisted peptide synthesis involving four steps in each cycle. Activated Fmoc-amino acid building blocks coupled to the PEG-linker; first diafiltration washes out the excess coupling reagents; piperidine is added to remove Fmoc; second diafiltration removes all the deprotection byproducts and reagents and the purified product is ready to repeat the cycle.

The concept of OSN membrane assisted peptide synthesis was first validated by So et al.^166,167^ They employed a linear 5,000 Da methoxy-amino-polyethylene glycol as the soluble anchor and an Inopor ZrO_2_-coated ceramic membrane with 3-nm pore size and hydrophobic surface modification to purify the intermediate products. Peptides were built on the soluble support via the following steps (Figure 17): (1) the coupling step to add a Fmoc-amino acid; (2) a purification step for the removal of excess reagents via constant volume diafiltration; (3) the deprotection step; (4) a second purification step for the removal of deprotection byproducts and excess reagents. The cycle was repeated for every new amino acid until the desired peptide sequence was achieved. Two pentapeptide sequences were successfully assembled through the process and a higher purity was achieved compared to that of peptides produced by SPPS. The result demonstrates that the OSN membrane-assisted peptide synthesis inherits the benefits of the liquid phase synthesis while avoiding the problematic purification step using precipitation or extraction.

The choice of membrane is vital for peptide synthesis, and the membrane must meet two criteria:^168^ one is that the membrane must show excellent chemical stability in aggressive reaction conditions for long durations. The other one is that the membrane should efficiently separate the peptide products from the residual byproducts and excess reagents. It was shown that the rejection of branched polyethylene glycol (PEG) by PBI membranes was higher than that of linear PEG with the same MW, in the range of 2,000–8,000 g mol^–1^.^163^ Instead of using a linear PEG as the support, Castro et al.^165^ designed three large globular PEG-based anchors (∼6,000–8,000 Da) to further improve the separation. PyPEG (∼6,200 Da) with a hydrophobic pyromellitic acid core and four aminopropyl-PEG branches showed 100% rejection by ceramic membranes and were successfully applied in the synthesis of a model peptide (Fmoc-RADA-NH_2_). Owing to its multiple conjugation sites at the ends of four or five polymer arms, PyPEG has a higher anchor loading capacity (∼0.6 mmol g^–1^) than that of linear PEG (∼0.2 mmol g^–1^). However, the loading capacity is still low considering its large MW. Furthermore, this bulky globular anchor faces issues of difficult chemical analysis and characterization due to its broad MW distribution.

**Figure 18:**
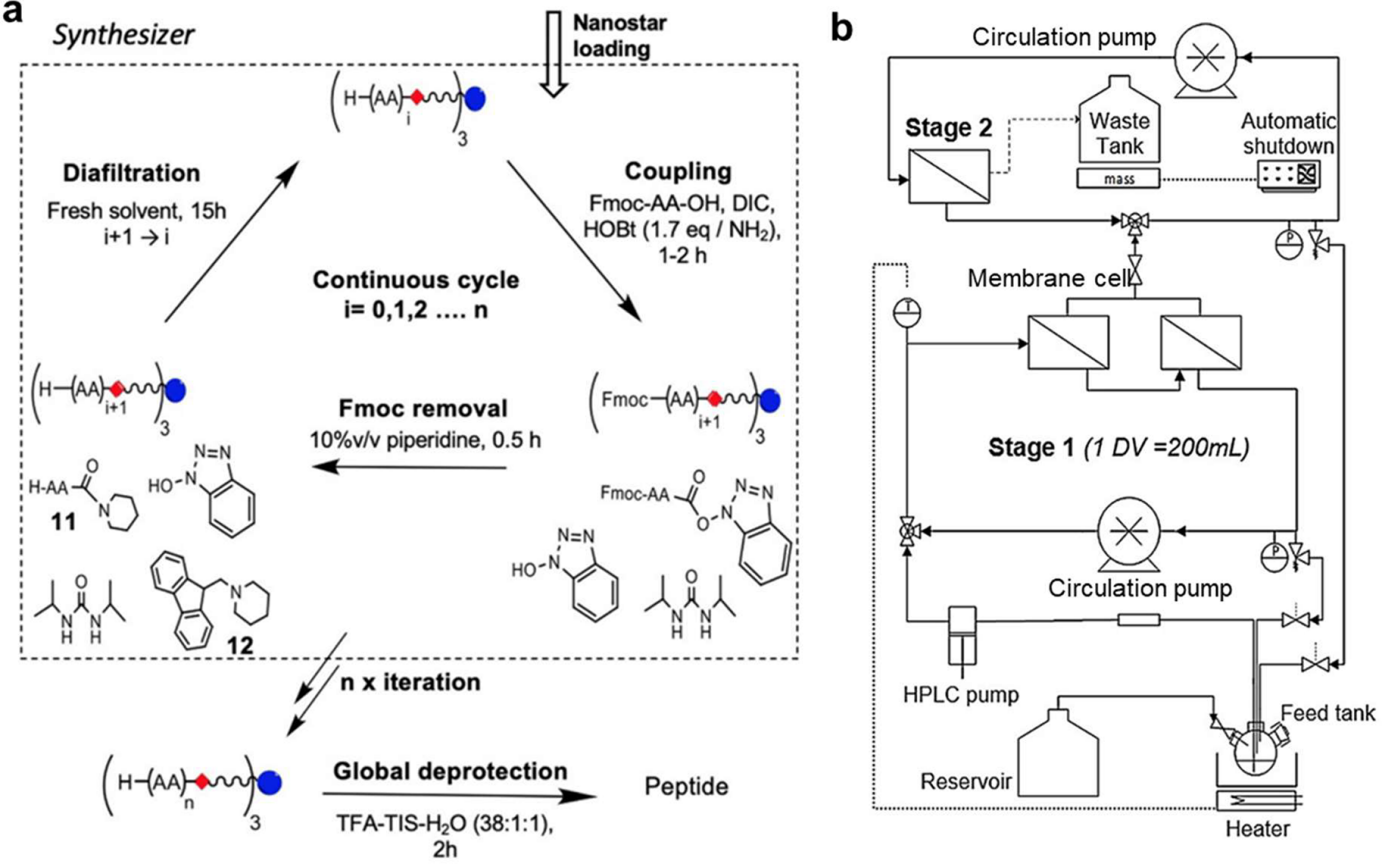
(a) Chain extension cycle of the liquid phase peptide synthesis via one-pot nanostar-sieving (PEPSTAR). Peptide-nanostars are grown via a three-step cycle of coupling, Fmoc removal, and diafiltration until the desired length is reached. (b) Schematic of the synthesizer layout for PEPSTAR. The whole process is conducted with the same equipment and a two-stage diafiltration setup is adopted. Reproduced from ref (168). Copyright 2021 Wiley-VCH GmbH.

To further increase the anchor loading capacity, Székely et al.^174^ designed a monodisperse PEG-armed and star-shaped support (homostar), which was prepared by iterative addition of monodisperse building blocks (ethylene glycol) onto an aromatic hub. The unimolecular and fully defined composition also enables easy characterization by liquid chromatography and mass spectrometry. The monodisperse PEG homostar has been successfully used to synthesize oligonucleotides,^163,164^ polyethers^169^ and peptides.^168^ Recently, the LPPS via one-pot nanostar-sieving (PEPSTAR) was demonstrated by Yeo et al.^168^ They designed a series of compact nanostar supports with a benzene core and three octaethylene glycol arms on the basis of the former homostar, which improved their loading capacities. For example, H-Rink-nanostar (MW 2120 Da) and HO-Wang-nanostar 4 (MW 1544 Da) have a loading capacity of 1.42 mmol g^–1^ and 1.94 mmol g^–1^, respectively. The PEPSTAR setup has several improvements compared with the previous setup. First, using the Fmoc strategy, the peptide is grown on the nanostar via a three-step synthesis cycle of coupling, Fmoc removal and diafiltration (Figure 18a). Compared to the conventional four-step synthesis cycle, the synthesis cycle only requires one diafiltration. The diafiltration step after coupling is eliminated as the piperidine in the deprotection step (Fmoc removal) can also quench excess amino acids in the coupling step. Second, the chemical reactions and diafiltration are conducted continuously inside the same equipment (Figure 18b). Third, a two-stage membrane cascade is adopted for the diafiltration process to improve the product yield loss from 40% to 10% without compromising the product purity (90%) (Figure 18b). Last but not least, three chemical resistant polymeric membranes (one polyethyleneimine and two PBI asymmetric membranes) have been developed for PEPSTAR. PBI_2005(1) was selected due to its high rejection to nanostar (93.3%) and low rejection to the largest MW byproducts (37.8%). The PEPSTAR setup was validated by the synthesis of Enkephalin-like model penta- and decapeptides, octreotate amide, and octreotate. The PMI for PEPSTAR (2983) is 3-fold lower than the conventional four-step method’s (9783) and slightly higher than SPPS’s (1726). However, the estimated cost of materials for PEPSTAR is only half of SPPS’s, since SPPS requires large excess usage of amid acids (3 equiv.) to achieve the specific purity.

**Figure 19:**
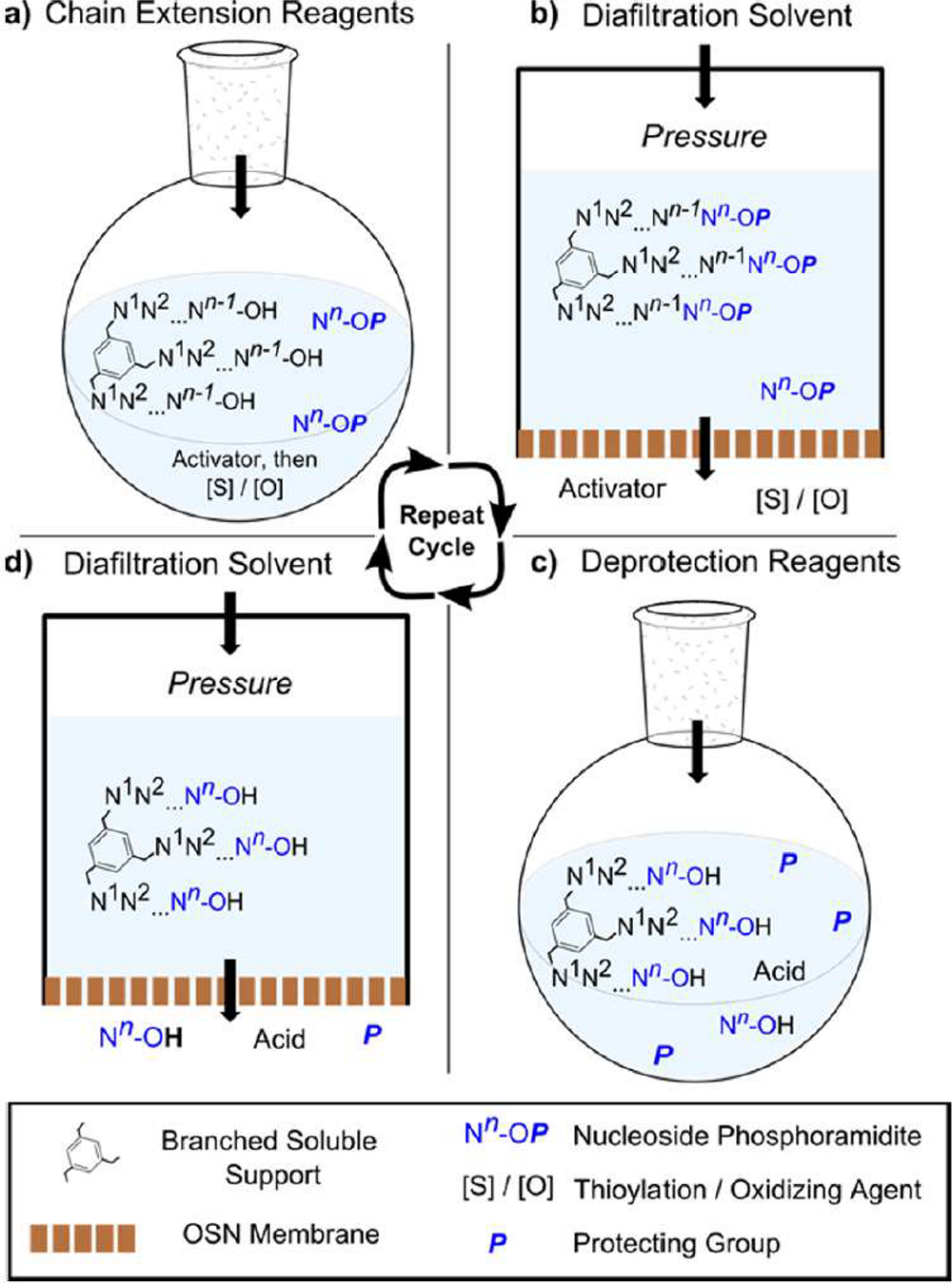
Schematic of the OSN-assisted oligonucleotide synthesis process. Each chain extension cycle includes (a) chain extension reaction (coupling and oxidation), (b) first diafiltration to wash out excess reagents, (c) deprotection for the next chain extension cycle, (d) second diafiltration to remove reaction debris. Reproduced with permission from ref (164). Copyright 2015 Wiley-VCH Verlag GmbH & Co.

Oligonucleotides are another novel class of drugs composed of nucleic acids with defined sequences with the potential to treat or manage a wide variety of diseases by modulating gene expression.^175^ The current state-of-the-art manufacture of oligonucleotides is the solid-phase phosphoramidite method, which has been used for almost 40 years.^176^ However, the process requires a large volume of hazardous reagents and solvents due to mass transfer limitations between the solid support and bulk solution, resulting in a high mass-intensity and difficulties in large-scale manufacture.^176^ As an alternative, the liquid phase oligonucleotide synthesis (LPOS) method does not have mass-transfer issues; however, it suffers from the cumbersome downstream purification required to remove excess reagents and byproducts after each iterative cycle. OSN, which facilitates the separation between the growing oligomers from excess reagents or impurities, has been proposed by Gaffney et al.^163,164^ to solve the separation issues of LPOS. The OSN-assisted oligonucleotide synthesis (Figure 19) consists of a four-step iterative growth cycle: (1) chain extension reaction; (2) first diafiltration to wash out excess reagents; (3) deprotection for the next chain extension cycle; (4) second diafiltration to remove reaction debris. A monodisperse tris(octagol) homostar was selected as the soluble support, which contributes to a convenient analysis by mass spectrometry, nuclear magnetic resonance, and high-performance liquid chromatography. An in-house PBI membrane was chosen for OSN diafiltration processes as it provided high rejections (>99%) toward homostar-oligo products and robust performance for over a year. The successful synthesis of a 2’-methyl RNA phosphorothioate 9-mer was demonstrated. Although the overall yield (39%) and purity (49%) of 9-mer was still low, OSN-assisted oligonucleotide synthesis still has a great potential to become an alternative for the large-scale synthesis of oligonucleotides after some further optimization. Several modifications were suggested by the author to improve the synthesis efficiency. For the optimization of membrane configuration, a two-stage diafiltration setup and a solvent recovery setup can be added to increase the overall product yield and reduce solvent consumption. Also, reducing the number of diafiltration in each iterative cycle from two to one can significantly save time and reduce the use of solvents.

As an emerging technology, OSN-assisted oligonucleotide and peptide synthesis is an attractive alternative to both the solid phase method and the traditional liquid phase method. However, there are still some challenging problems to be solved. From the membrane side, the insufficient separation of the intermediate (anchor-peptides/oligos) from byproducts and excess reagents in the diafiltration stage is still an issue. The rejection may be improved by increasing the size of the anchor with longer PEG chains; however, it is at expense of the overall loading capacity, where a high loading capacity is advantageous to reduce the total cost.^177^ Also, in the PEPSTAR system, a two-stage diafiltration setup was necessary to compensate for the low rejection of the PBI membrane. Although a high yield was achieved with the two-stage diafiltration, a single-stage diafiltration with a high rejection membrane is obviously more attractive since it provides the advantages of short operation time, less solvent consumption, and process simplicity. Thus, a membrane with higher rejection and selectivity toward the anchor-peptide/oligos should be explored.

### 4.5 Solvent Exchange

In the pharmaceutical industry, solvent exchange is regarded as one of the major solvent consuming processes in API manufacturing due to the need for different organic solvents depending on the type of chemistry in each synthetic step.^178^ Furthermore, the purification and isolation of intermediates also require a large amount of solvent.^6^ Traditionally, solvent exchange is achieved by distillation, which removes the first solvent followed by the addition of a second solvent. One limitation of distillation is that it can only be used efficiently to replace the lower boiling point solvent with a higher boiling point solvent. In addition to the high energy consumption of traditional distillation, some molecules are also heat-sensitive resulting in degradation.^178^ Under this circumstance, OSN, which is more energy efficient, operates at ambient temperature, and with the potential to save solvent (combined with solvent recovery), has gained great attention. Therefore, OSN is regarded as a good method for solvent swaps as it is easy to operate and scale up.^179^

Similar to API purification, a common system that is used for solvent exchange is constant volume diafiltration. During the solvent exchange process, the old solvent permeates through the membrane and is replaced by the new solvent, in the meantime the target compound is retained by the membrane (Figure 3b). To achieve the maximum exchange while maintaining as much target compound as possible, a proper membrane as well as a well-designed system are needed.

A guideline to assist in successfully implementing OSN for solvent exchange using constant volume diafiltration is as follows:^180^

(a) Performing membrane screening. The suitable OSN membranes used for solvent exchange should provide proper stability in interested solvents, a reasonable flux during the operations (≥10 L·m^–2^·h^–1^), and a sufficient rejection for the solutes to be retained.^25^
(b) Estimating the required amount of diafiltration solution. The miscibility of two solvents is vital, because the immiscibility will cause heterogeneous liquid phase transport, leading to unreliable permeate concentration.
(c) Investigating different factors that have effects on the permeate flux, such as temperature and solvent concentration. During the diafiltration process, permeate flux is the most important factor, because it informs the filtration time and/or the required membrane surface area for industrial scale operations.
(d) Deciding the optimal operational mode by testing the flux, diafiltration solution consumption, and operability. The mode can be either discontinuous, semicontinuous or continuous.

Since the diafiltration theory and operation are straightforward, it has been applied to a lot of studies in the pharmaceutical industry. Researchers have focused on screening commercially available OSN membranes in simple solvent exchange tests. For example, Sheth et al. used MPF-50 and MPF-60 (Koch Separation Solutions) to investigate the solvent exchange from Ethyl acetate to methanol with erythromycin as the solute in the system. Ethyl acetate was reduced down to 4% after two diafiltration cycles.^181^ Lin et al. proposed a continuous process for solvent exchange from toluene to methanol using a membrane cascade containing StarMem^TM^ 122. The results showed 47.8%, 59.2% and 75.3% solvent exchange for single-stage, two-stage, and three-stage cascades.^178^ Anjum and coauthors also tested solvent exchange processes during API crystal suspension purification. They used OSN to replace the residual organic solvent with water after the antisolvent crystallization. Naproxen was used as the target compound, with ethanol and water as the solvent and antisolvent respectively. They performed the membrane screening of DuraMem 300, AMS NanoPro, SolSep 090101 and 070706, and GMT-oNF. The results showed that DuraMem 300 had the best performance. Hence, it was selected to further investigate the solvent exchange from ethanol to water after antisolvent crystallization. The experiment was carried out through both discontinuous and semicontinuous diafiltration modes and showed that the exchange of Naproxen suspension in 5% ethanol to water can be achieved in a four-stage diafiltration process, using 1.5 g of water per g of feed.^180^

The feasibility of using OSN as assistance for traditional separation techniques during pharmaceutical processes was also investigated by Rundquist et al. They applied OSN to counter-current chromatography (CCC) during pharmaceutical separations.^182^ Applications of CCC usually start with solvent exchange to transfer the solute from the process solvent to the desired solvent mixture for the mobile phase. The purpose of this study was to transfer an initial crystallization mother liquor (82% methanol, 15.9% methyl isobutyl ketone, 2.1% toluene containing 4.5 g L^–1^ API and some impurities) to a selected CCC mobile phase (67.32% heptane, 30.29% ethyl acetate, 2.16% methanol and 0.24% water) using a METCell dead-end filtration system equipped with Starmem^TM^ 122. Fresh ethyl acetate was used as the diafiltration solvent. The whole exchange process contained several put and take diafiltration processes, and each diafiltration cycle started with a concentration of feed solution by removing 70% of the original solvent, followed by adding pure ethyl acetate to a volume of 200 mL. The diafiltration cycle was repeated until the desired solvent composition was reached. The results showed that for a starting 400 mL feed solution containing 50% mother liquor, the desired solvent composition was reached after 8 cycles, requiring 5.9 diavolumes. The OSN coupled CCC process improved the mass intensity, and the solvent consumption was reduced by 56%.

In the last 10 years, some companies have discontinued some of their OSN series (for example, MPF-50 and MPF-60 from Koch and DuraMem from Evonik), and the selection of currently available OSN membranes is limited (shown in Table 1). Fortunately, the currently commercially available OSN membranes are known to be resistant to harsh chemical conditions while rejecting small solutes; hence, it is possible to utilize them in innovative configurations.^78^ Among them, a membrane cascade is of great interest, because it could overcome the insufficient separation limitations and minimize organic solvent use.^79^ Furthermore, it could also assist the continuous downstream processing. The integration of OSN membrane modules and flow chemistry synthesis have made great progress in the past decade.^179,183^

The principle of a membrane cascade is that the feed solution passes through several membranes consecutively. The membranes could possess similar or different materials and MWCO.^184^ An example schematic description of membrane cascade is shown in Figure 10. Based on the purpose of individual applications, several single membrane cells could be arranged in parallel or series in a cascade.^178^

However, several challenges still need to be hurdled, such as difficulties in controlling operational variables (pressure, flow, concentration, etc.),^185^ lack of performance analysis as a function of operational variable and cascade design,^186^ and limited availability of experimental data to support membrane performance prediction model development. As suggested by Lightfoot et al.^185^ and Siew et al.,^62^ the biggest challenge in implementing the membrane cascade is the delicate control of interacting flows. Inadequate control will lead to both poor selectivity and worse overall performance compared with a single-stage process.^187^ Also, the large number of cascades resulting in better outcomes should be balanced against the resulting complexity of the process requiring additional storage tanks, pumps, and analytical tools. A simplified configuration is appreciated because it makes the reconfiguration easier regarding different campaigns, and a versatile system is essential for lowering the capital cost.^77,188^ A better implementation of membrane cascade should involve the control of permeate flux from individual stages directly by a flow controller. This controller will regulate the retentate flow from the single stage and the solute rejection, which is correlated to the flux in each stage.^62^

**Figure 20:**
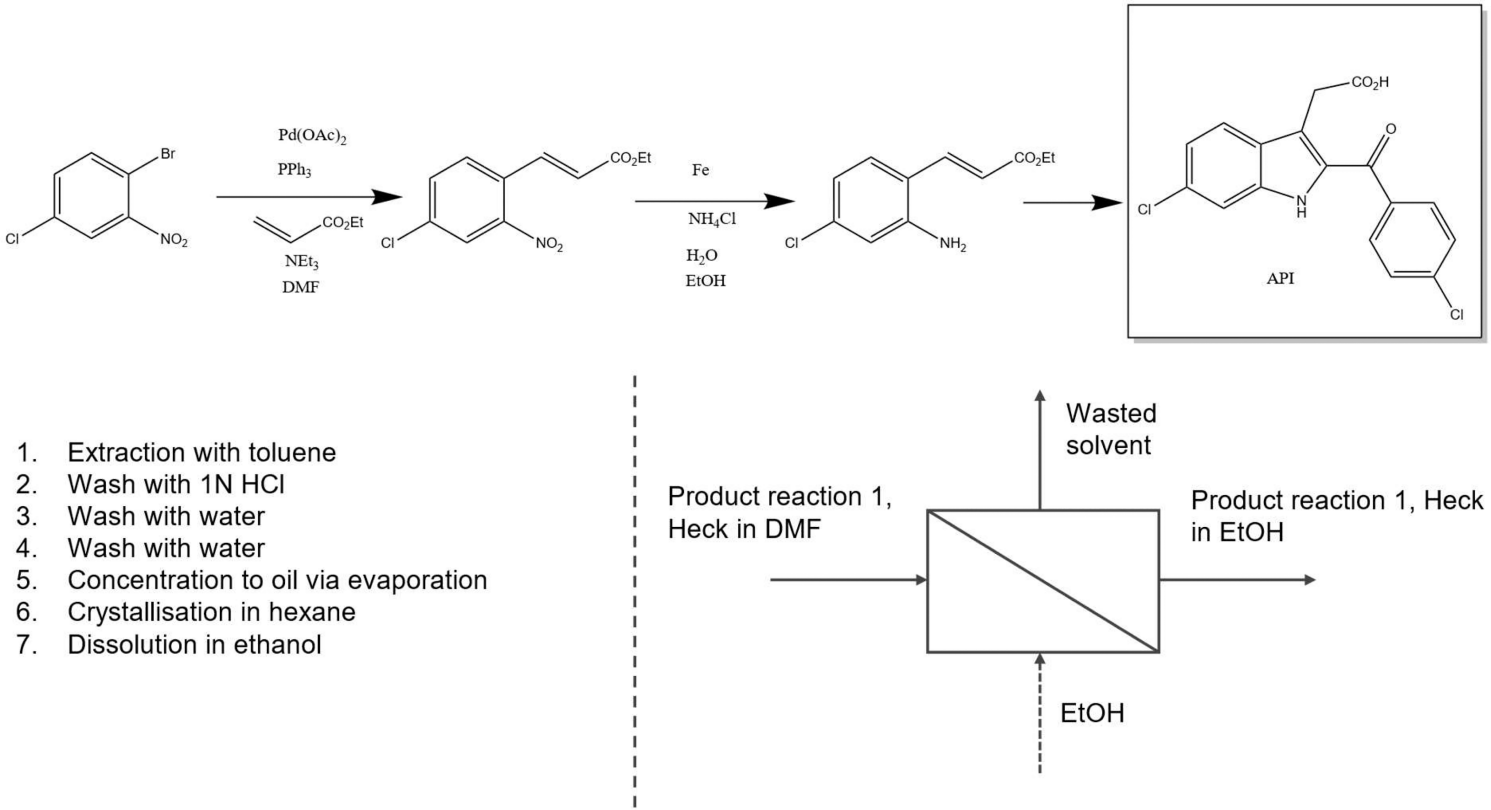
Reaction scheme and comparison of published process and OSN as an alternative route for solvent exchange. Reproduced with permission from ref (179). Copyright 2016 Wiley-VCH Verlag GmbH & Co.

Peeva and coauthors^179^ have provided a typical example of applying OSN as an alternative in downstream processing. They have investigated the solvent exchange by OSN in a continuous consecutive Heck coupling reaction, where DMF is continuously replaced by ethanol (Figure 20). The original published traditional synthesis process contained seven steps involving a great number of solvents and time-consuming solvent exchanges. By replacing the traditional solvent exchange steps with a membrane cascade unit, the whole process was simplified and showed good results (Figure 20). DuraMem 150 was used in this study after screening four different membranes (both in-house fabricated and commercial membranes).

**Figure 21:**
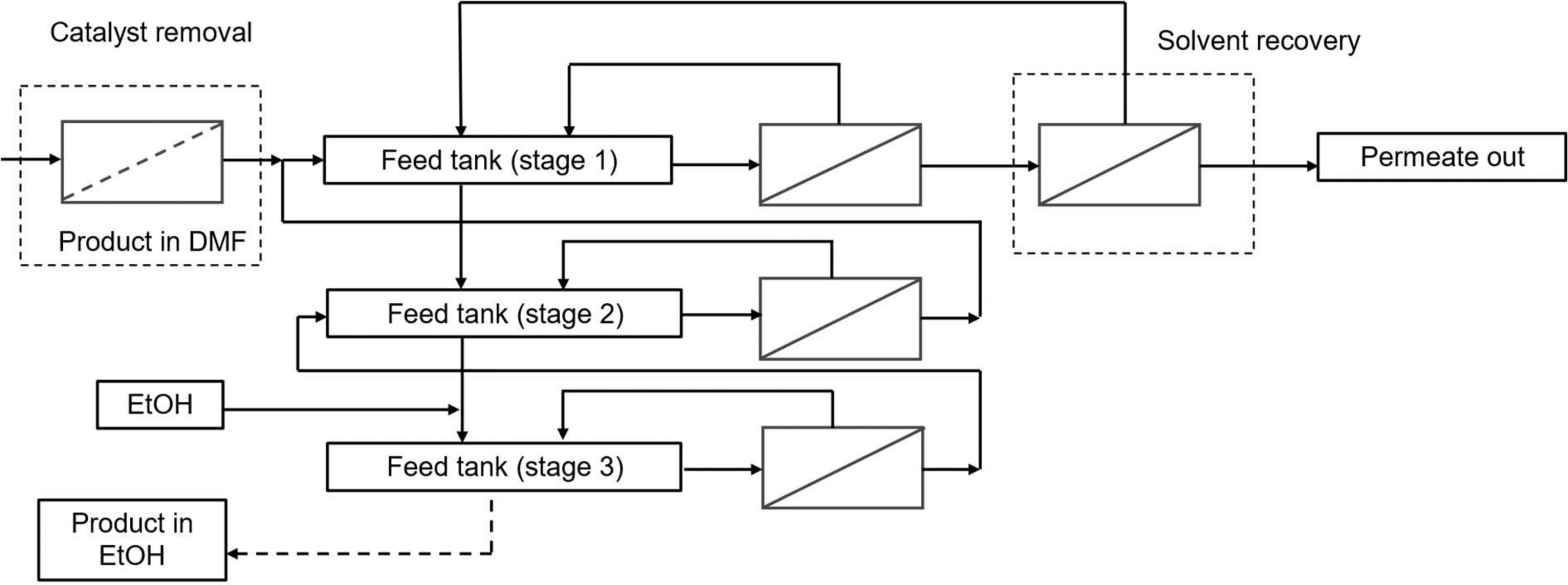
Process diagram of the continuous process where the Heck reaction and solvent exchange were performed. Reproduced with permission from ref (179). Copyright 2016 Wiley-VCH Verlag GmbH & Co.

The whole concept of the continuous process is shown in Figure 21. The cascade consisted of 3 stages and was operated in counter-current mode.^178^ Before the product solution was transferred to the solvent exchange operation, the catalyst used in the reaction was first removed by an OSN unit. Permeate from each stage was fed into the feed tank of the previous stage, and the permeate from the first stage was fed directly into a recovery stage to increase the product yield. Each stage (except for the recovery stage) had three circular crossflow cells connected in series, and each cell held a membrane disk with a 51 cm^2^ surface area. The overflow of each feed tank was transferred to the feed tank of the next stage. The final product stream in ethanol was collected as the overflow from the feed tank in stage 3. The results indicated that the stream was transferred from 100% DMF to 82% ethanol, with a product dilution factor of 3 and product yield of greater than 99%.^179^

### 4.6 Solvent Recovery

In the pharmaceutical industry, batch processes that contain multiple reaction steps are utilized in most API production and require a large number of different organic solvents. Solvents are not only used for reactions but also often used in purification steps and for analytical processes.^3^ In a life cycle analysis of API production, the use of organic solvents takes up more than 95% by mass of the total raw materials, and around 60% of the overall energy consumption.^8^ Hence, the recovery of the organic solvent is of great interest to reduce waste production and energy consumption, which further lowers the capital and environmental costs of the manufacturing process.^189^

The majority of the wasted solvent within the pharmaceutical industry is still disposed through on-site combustion or outsourced services.^5^ This is not only due to economic considerations, but is also due to the resistance of implementing new processes and techniques in the late phase of the development of new drugs which needs to be recorded and approved by authorities. However, as the environmental legislation is getting stricter and the price of virgin solvents is becoming more expensive, a need for studying and developing a more competitive solvent recovery method is of great importance.^74,190^ Among various technologies available for the purification and recovery of organic solvents, OSN is regarded as one of the most important because of its low operation cost and high energy efficiency.^76^ Another advantage of using the OSN process is its modular nature.^191^ Since the membrane units have small footprints, they are easy to handle and integrate with existing methods, and can be used as the final stage of downstream processing using membrane cascade.^3,192^

One important process where OSN has big potential is in the recovery of solvent used in the crystallizations of APIs and building blocks. Crystallization can generate huge amounts of solute rich mother liquors containing both impurities removed from operation and API in low concentration. Instead of disposing of the mother liquor, recovering the solvent as well as the valuable API from mother liquors could be a remunerative way for boosting the mass-efficiency of the production.

**Figure 22:**
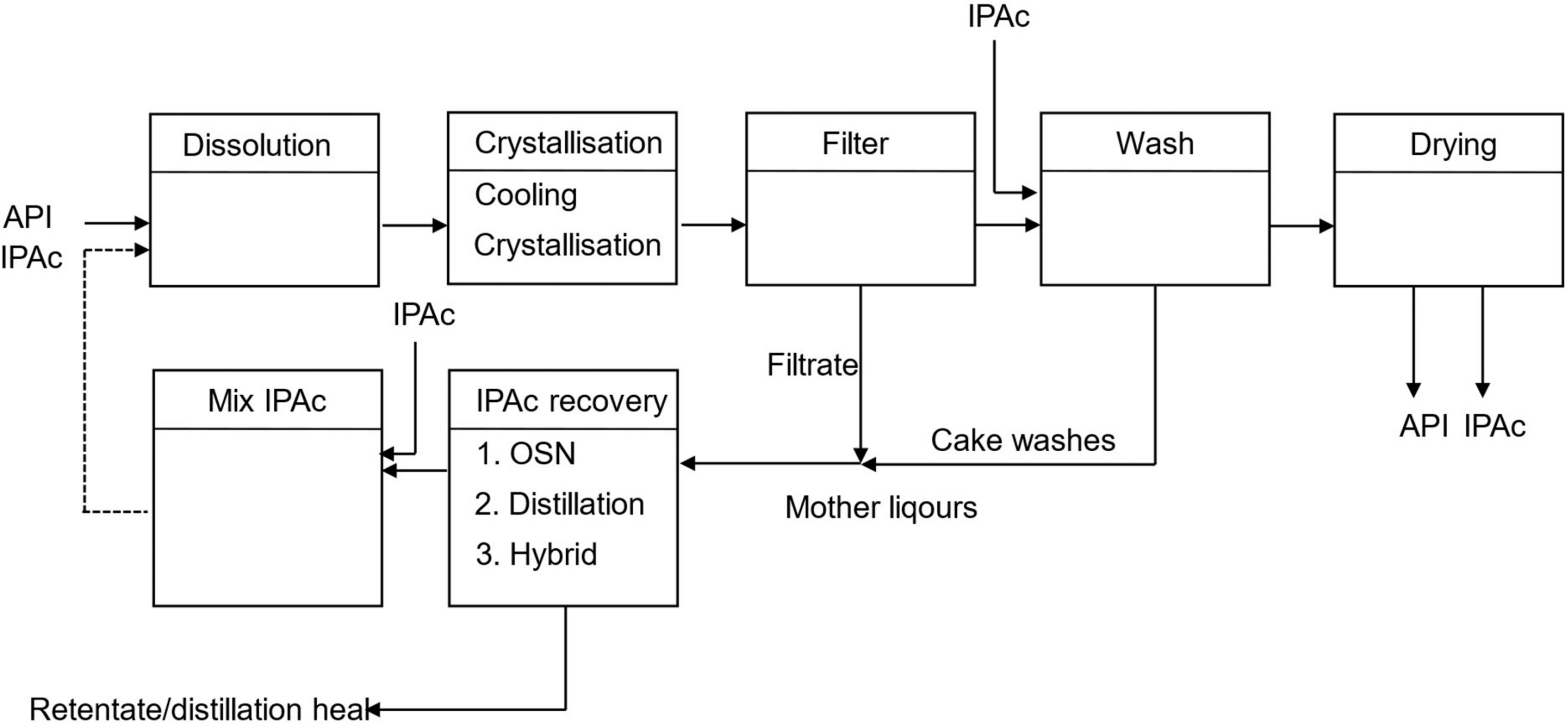
Process flow diagram of isopropyl acetate recovery from mother liquors. Reproduced with permission from ref (74). Copyright 2012 The Royal Society of Chemistry.

Rundquist et al. investigated the feasibility of substituting distillation with OSN to recover isopropyl acetate from crystallization mother liquors containing dissolved API (MW around 600 g mol^–1^), more than 40 types of organic impurities and a trace amount of methanol, water, and isopropyl alcohol. The recovered solvent was intended to be recirculated into the crystallization process (Figure 22).^74^

Three types of membranes, Starmem^TM^ 122, Starmem^TM^240 and Puramem^TM^280, were selected for screening. The screening experiments were performed under 30 and 60 bar to investigate the effect of operational pressure on the membrane performance. The Starmem^TM^ 122 (>99.9% API rejection, 36–40 L·m^–2^·h^–1^ flux at 30 bar) and Puramem^TM^280 (>98% API rejection, 54 L·m^–2^·h^–1^ flux at 60 bar) were selected for lab-scale solvent recovery investigation. Then Puramem^TM^280 was used to perform a pilot-scale experiment. The purity of the recovered solvent was tested by recycling the solvent back to 4 subsequent API crystallization batches. In this study, the maximum amount of solvent that could be recovered by OSN was limited to 80%, while the distillation could achieve 90% recovery. An equivalent 90% recovery volume could be reached by recovering 80% using OSN and using distillation to continue until it reaches 90%. By using this hybrid process, the energy consumption was 9 times lower compared with distillation.^74^

**Figure 23:**
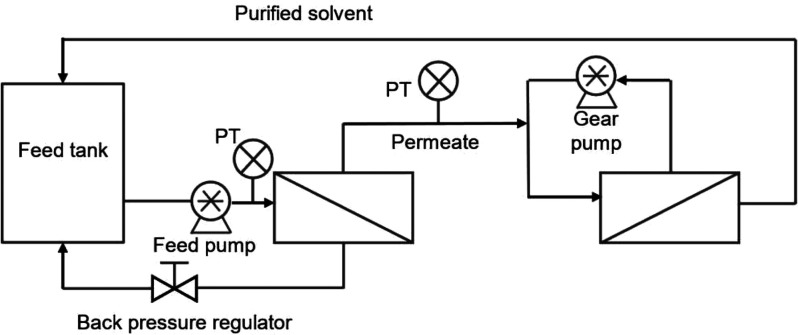
Schematic description of in situ solvent recovery. Reproduced from ref (76). Copyright 2014 American Chemical Society.

OSN solvent recovery has also been coupled to the diafiltration process to enhance sustainability. Kim et al. proposed a solvent recovery platform that purified API solution while recovering solvents in a single in situ unit.^76^ The API solution was purified by constant volume diafiltration using two 22DBX membranes, and two DuraMem 150 were utilized in the solvent recovery unit. The recovered solvent was pumped back to the initial feed tank. The schematic description is shown in Figure 23.

The feed solution contained 10 g L^–1^ roxithromycin and 1 g L^–1^ triphenylmethanol dissolved in methanol. The diafiltration was operated at a flow of 40 L min^–1^ and 21 °C. The initial pressure in the diafiltration unit was set to 5 bar, which incrementally increased when the solvent recovery pressure increased. The operational pressure for the diafiltration stage and solvent recovery stage were 13 and 8 bar, respectively, leading to the transmembrane pressure of 5 and 8 bar, respectively. Without liquid entering or leaving, this proposed system could run without extra intervention until the purity was stable. Results showed that around 98% impurity removal could be obtained without adding extra fresh solvent.

The study also provided an example for sustainability assessment by comparing the CO_2_ footprint of recovery through OSN solvent recovery with conventional distillation and adsorption. Calculating the carbon footprint is considered as an efficient way to assess the downstream sustainability since it involves both energy and solvent consumption, as well as generated waste.^76^ In this study, the calculation of carbon footprint involves the CO_2_ generated from electricity (pump power consumption), waste adsorbent, solvent, and membrane disposal. The main CO_2_ footprint contributor for the adsorptive solvent recovery process is the considerable amount of solid waste generated, as well as the need for frequent replacement of adsorbent. As for distillation solvent recovery, the high CO_2_ footprint comes from very high energy consumption. Regarding OSN solvent recovery, the only two CO_2_ contributors are the membrane solid waste and the power consumption of the pumps, while the membrane solid waste is negligible because they usually possess a lifetime greater than 2 years if properly maintained.^76^ The calculated results suggested that the proposed OSN-based solvent recovery reduced the CO_2_ production from 3200 to 150 kg CO_2_ per kg product, corresponding to 95% CO_2_ reduction.^76^ Calculating the CO_2_ footprint is the best way to evaluate the sustainability of a downstream process, because it involves both energy and solvent consumption, as well as generated waste.^76^ The significant reduction of carbon footprint in the OSN process was mainly from the elimination of solvent incineration.

Apart from CO_2_ footprint, mass intensity (MI), solvent intensity (SI), cost and energy consumption were also used as green metrics for the OSN solvent recovery process.^78^ Mass intensity and solvent intensity are two metrics that are used to describe a specific process.^78^ They are defined as
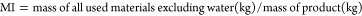
3
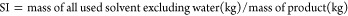
4When comparing two processes, mass intensity ratio (MIR) is used, which is the ratio between mass intensity of process 1 (denoted as MI_1_) and mass intensity of process 2 (denoted as MI_2_). The relation is described below:
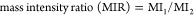
5When the mass intensity ratio is less than 1, MI_1_ is preferred, and vice versa.^78^ Generally, a process involving a solvent recovery unit has a significant improvement in sustainability regarding mass intensity and solvent intensity.

**Figure 24:**
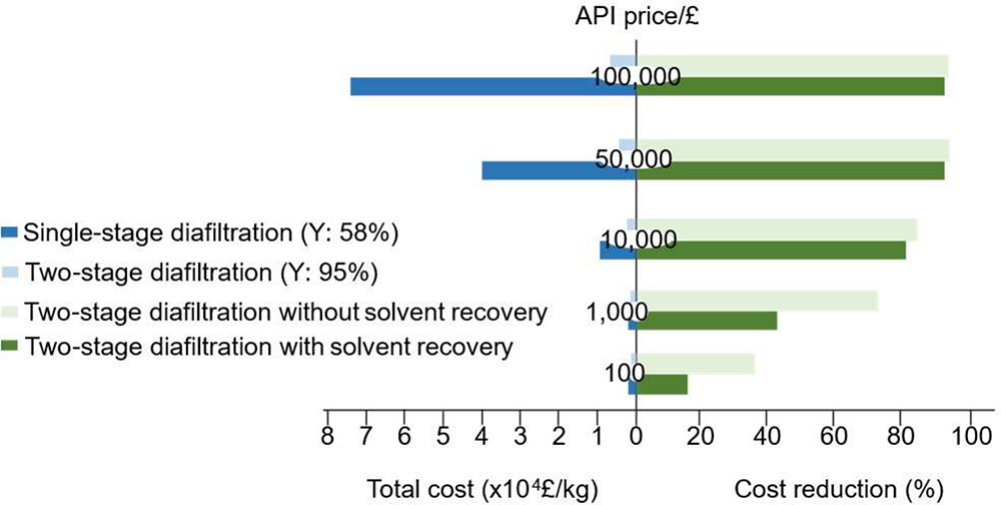
(left) Comparison of total cost of single-stage and two-stage diafiltrations. (right) Comparison of cost reduction of two-stage diafiltration with and without solvent recovery. Reproduced with permission from ref (78). Copyright 2014 American Chemical Society

A study by Kim et al.^78^ compared those green metrics of single-stage diafiltration process, two-stage diafiltration process and two-stage diafiltration process with solvent recovery. Since the economic gain from solvent recovery is mainly related to the scale of API production, and the reduction of environmental impact from the process is mainly dependent on the amount of solvent used and recycled, a reasonable comparison of the three processes was performed at different API prices. A summary of the cost comparison between single-stage and two-stage diafiltration with and without solvent recovery is shown in Figure 24.

According to Figure 24, when the API price was at the highest (100,000 £/kg), the cost was reduced by 92% when OSN solvent recovery was implemented. As the price of API decreased, the cost reduction also decreased. Hence, API price is the main factor affecting the cost reduction by implementing OSN. The results also showed that two-stage diafiltration with solvent recovery contributed to significant cost saving compared with single-stage diafiltration without solvent recovery. The calculated MI and SI for single-stage diafiltration, and two-stage diafiltration with and without solvent recovery were compared. Results showed that implementing solvent recovery in a two-stage diafiltration resulted in 70% and 73% MI and SI reduction, separately, compared to single-stage diafiltration.

There are also other studies on solvent recovery using either in-house fabricated or commercially available membranes. Schaepertoens et al. has screened 9 types of membranes, both commercial (GMT, Novamem, Solsep) and in-house fabricated (noncross-linked and cross-linked polybenzimidazole membranes), using acetone as model solvent in a semicontinuous diafiltration mode. The results showed that the PI-PEEK membrane was the best one which possessed high rejection of the impurity and would be suitable for solvent recovery.^193^ Tashvigh et al. synthesized a type of PBI hollow fiber membrane that was doped with a 2% H_2_SO_4_ solution. The formation of hydrogen bonds between acid molecules and the PBI backbone led to an integrated structure, which made the membrane more compatible with organic solvent. They used tetracycline/methanol and L-α-lecithin/hexane as a model compound mixture, and the membrane showed high rejection (>98%) and permeance 3.5 and 7.1 L·m^–2^·h^–1^·bar^–1^, respectively, toward methanol and hexane, which makes this membrane potentially suitable for solvent recovery.^194^ Fodi et al. have investigated the solvent and reagent recovery and recycling during Michael Addition of nitromethane to trans-chalcone using DuraMem 150. The membrane unit was connected to the packed-bed flow reactor, and continuous solvent and reagent recycling was developed. This hybrid process was operated for 6 weeks, leading to around 90% solvent recovery.^195^ Ormerod et al. have used OSN for in-line solvent recycling in the oxidative cyclization of (1–9)NH_2_DDAVP 1 to the cyclic peptide desmopressin 2. The membrane chosen for this study was a 50 cm, single tube, 0.9 nm ceramic membrane from Inopor, which showed 98.6% and 99.1% rejection to both molecules. The diafiltration result showed that the solvent consumption could be reduced by up to 83% without detrimental influence on product yield and purity.^196^

One of the biggest challenges when implementing OSN into solvent recovery is insufficient rejection to small MW solutes. To purify solvents that contain small-sized molecules, tight membranes are needed to achieve almost full rejection. However, the tighter the membrane, the lower the flux, leading to longer operating times.^3^ So far, most of the current commercial membranes do not retain small molecules in a single-stage membrane process.^23^ A DuraMem 150 from Evonik was available on the market but has now been withdrawn. The AMS NanoPro S-3011 with MWCO 100 Da is one of a few available membranes for small molecule applications, but it has only been applied to the mixture of solvent and water, or polar solvent such as methanol.

## 5 Challenges and Future Perspectives

Despite the promising potential, there are still some obstacles that hinder the broad applications of membrane technology in the pharmaceutical industry, the major one being the regulatory restriction of using most commercial membranes in GMP manufacturers. In addition, insufficient separation between different solutes, low rejection of small molecules, and large solvent consumption are the major drawbacks.^179^Table 4 summarizes the current challenges and possible solutions in pharmaceutical applications.

**Table 4:** Summary of Current Challenges and Possible Solutions in Different Pharmaceutical Applications

Applications	Challenges	Possible solutions
API concentration	Insufficient rejection toward API	Novel membrane; membrane cascade
API purification	Insufficient separation; low product yield	Novel membrane; membrane cascade; Hybrid process: coupling with other downstream units, e.g., adsorption, chromatography
	High amount of solvent usage	Add a solvent recovery unit
Homogeneous catalyst removal and recovery	Insufficient separation; Inadequate catalyst stability for reusing	Novel membrane; membrane cascade; size-enlarged catalyst; more research on the choice of ligand
OSN-assisted synthesis	Insufficient separation	Novel membrane; size-enlarged anchor; membrane cascade
	Lack of stability and fouling studies in a harsh reactive environment	Perform more studies in both short-term and long-term
Solvent exchange	Low rejection leads to low product yield	Novel membrane; membrane cascade
	High amount of solvent usage	Combine with solvent recovery unit; membrane cascade
Solvent recovery	Low rejection for small impurities	Novel membrane; membrane cascade
Coupling to continuous process	Challenging to design and set up	
Sustainable scaling up	Mass transfer and pressure drop	Develop more delicate models to understand how OSN works
	Limited number of commercially available OSN membranes	Combine available membranes effectively
	Economy concern	Capital cost is supposed to constantly decrease with increasing demand and development of membrane technology
	Lack of experimental data that supports the process design	Maybe machine learning or other computational tools could be used

Based on this summary, research that have been performed is discussed in the following section.

### 5.1 Enhancing Overall Separation Performance

In the pharmaceutical industry, efficient separation is critical for OSN applications. Both API concentration/purification, solvent exchange and solvent recovery require tight membranes, which can perform one of the following functions: separate two or several different solutes; sufficiently retain solutes while permeating solvents; offer a specific MWCO to separate solutes with similar MW. However, typical OSN membranes show a sigmoidal curve for rejection as a function of molecular size, indicating that sufficient separation among solutes could only be achieved when the size difference is large.^77^ In other words, the similar MW among different solutes limits the separation efficiency. Therefore, there is a need for the continuous development of more selective membranes. Table 5 lists some membranes that have enhanced selectivity and are suitable for several pharmaceutical applications. Both the introduction of emerging polymers and innovative designs of membrane fabrication/post-treatment have contributed to enhancing performance.

**Table 5:** Recent Research on Membranes with Enhanced Stability or Better Selectivity

Membrane type	Modification performance	Potential applications
Polymer brush membrane cross-linking with aromatic trimesic acid and aliphatic itaconic acid^197^	High selectivity for methanol-toluene separation	Solvent exchange; Solvent separation
Polymer brush membrane grafted with short and long hydroxyethyl methacrylate structures as cross-linkers^198^	High selectivity and reasonable permeability for commercially relevant methanol/toluene separation	Solvent exchange; Solvent separation
TFC polyamide membrane with adamantane diamine as a molecular building block^199^	High methanol permeance with 94.7% organic dye rejection; the MWCO is down to 327 Da; good resistance toward organic solvents (180 h continuous filtration in DMF)	Solvent recovery; Filtration in harsh conditions
PBI OSN membrane cross-linked by KMnO_4_^200^	Superior separation performance and enhanced stability in organic solvents including *N*-methyl-2-pyrrolidone; 90% rejection for dyes in the MWCO range of 327 to 1017 Da	Filtration in harsh conditions (polar and nonpolar solvents)
Free-standing sub-10 nm polyamide nanofilms^201^	Two orders of magnitude higher acetonitrile permeance than commercially available OSN membranes; good stability; can separate small molecules with high efficiency	Solvent recovery
GMF-NH_2_ enhanced membrane using trifluoromethyl groups in polyamide layer^202^	Rapid methanol recycling, with the methanol permeance 11.72 ± 0.98 L·m^–2^·h^–1^·bar^–1^ and Chlorazol black rejection of 99.5 ± 0.1%	Solvent recovery
Hydrophobic substrates promoted lysozyme nanofilm composite membranes^203^	High permeability as well as high selectivity; stable in a wide range of organic solvent	Solvent recovery; Catalyst recycling
Microwave-assisted nanoporous graphene membrane^204^	Ultrafast organic solvent permeability and high stability; MWCO was tuneable from 500 Da to subnanometre-size, which depends on the type of solvent	Multiple solutes separation in a single membrane
Epoxy-containing inorganic networks cross-linked polybenzimidazole membrane^32^	Ethanol permeance of 27.74 L m^–2^ h^–1^ bar^–1^ with > 90% eosin Y rejection, stable under extremely basic condition	Filtration under extreme basic conditions
Robust polyamide-PTEE TFC hollow fiber membrane^205^	High CAN and DMF permeabilities with > 90% acid fuchsin rejection. MWCO lowered to ∼ 300 Da, 72 h stability in DMF	Harsh organic solvent nanofiltration

The large-scale production of novel membranes is not an easy task. This is due to the extremely precise conditions required when creating a high number of regular features.^206^ To achieve pharmaceutical industrial scale applications, techniques that are more versatile are needed.

### 5.2 Coupling to Continuous Process

OSN presents an attractive approach for implementation into a continuous process due to its ease of operation regarding flow, scalability, and no phase transition. OSN has been demonstrated for the concentration and purification of process streams,^79^ catalysts removal and recycling,^179^ and solvent exchange.^179^ However, the integration of individual steps into a complete multistep continuous process is challenging.^207^ Each reactor unit must be designed to ensure compatibility with the subsequent unit regarding the flow rate, pressure, pH, solvent condition and temperature.^179^ For example, the operational temperature threshold for current commercially available polymeric membranes is mainly between 50 and 60 °C (PuraMem, Borsig, AMS); hence above that limit, precooling is needed prior to entering the membrane stage.

### 5.3 Exploring Nonsize-Based Membranes

The solute size is essential for membrane selectivity, but it is not the only factor that affects the selectivity.^208^ Solute separation based on their affinities to membrane and competition with solvent have been widely studied and show potential in the purification process.

#### 5.3.1 Enantiomers’ Separation Membranes

Chiral separation is attractive in the pharmaceutical industry because many drugs are chiral compounds, but there are not always selective synthetic methods available to synthesize the pure enantiomers. The separation of enantiomers from racemic mixtures has always been on the frontier of research as a complement to traditional asymmetric synthesis.^209^ Among the reported separation technologies, membrane separation without phase transition is regarded as the emerging technique due to its operational simplicity and low energy demand (less fresh solvent needed and lower pressure requirement). However, being each other’s mirror images with identical chemical and physical properties makes them challenging to separate, because most commercially available membranes are size exclusion based.

Chiral separation membranes can be categorized into liquid membranes and solid membranes. Although liquid chiral membranes have rapid mass transfer, they suffer from poor mechanical stability and durability. Hence, solid membranes are regarded as the most suitable ones in large-scale chiral enantiomer separation in the pharmaceutical industry.^209^ Currently, chiral separation has been investigated by membranes that are based on polymers, carbon nanomaterials, and metal organic frameworks. Ong et al. have fabricated a TFC chiral separation membrane using (2-hydroxypropyl)-beta-cyclodextrin (HP-β-CD) as the chiral selector for enantiomeric separation of racemic 1-phenylethanol chiral compounds. The obtained membrane achieved 60–80% enantioselectivity of R-phenylethanol over S-phenylethanol.^210^ Zhu et al. have prepared a mixed matrix membrane using cellulose acetate as membrane polymer matrix, graphene oxide as the modifier and mono(6-ethylenediamine-6-deoxy)-β-cyclodextrin (EDA-β-CD) as chiral selector. The enantiomeric separation performance was investigated by testing the separation of chiral drugs. The obtained membrane showed 3% better selectivity for racemic R/S-tryptophan than the nongraphene oxide membrane.^211^ Milovanovic and coauthors have developed an organic–inorganic double network within an organic polymeric membrane for chiral separation. The designed membrane showed good separation of R- and S-naproxen. It also possessed excellent mechanical stability and high solute permeabilities.^212^ In spite of the increasing amount of research in membranes for enantioseparation applications, their use is still limited to small scales for some exploratory projects about pharmaceuticals and amino acids.^209^ More research is needed in this field to obtain membranes that are practical to use on larger scales and add value for industrial applications.

#### 5.3.2 Molecularly Imprinted Membranes

**Figure 25:**
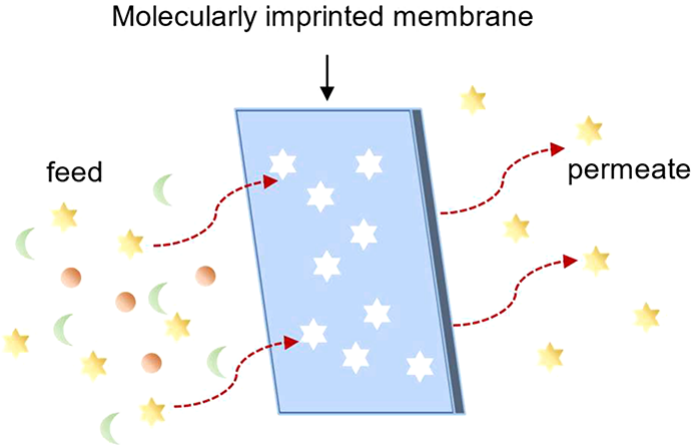
A schematic description of the molecularly imprinted membrane. Reproduced from ref (213). Copyright 2016 American Chemical Society.

Membrane technology could also be combined with molecular imprinting technology. By chemical and physiochemical interactions, a template could be incorporated into a polymer matrix. When the template is removed, a binding site (or recognition site) will be created, which has a specific shape and electronic environment and can selectively separate the target molecule.^25,213^Figure 25 is a schematic description of a molecularly imprinted membrane.

Compared with traditional size-based membranes, molecularly imprinted membranes provide the extra advantage of solute selectivity. Men et al. have prepared a polyvinyl alcohol membrane using (S)-amlodiphone (S-ADP) as the template, methacrylic acid as the functional monomer, and N, N’-methylenebisacrylamide as the cross-linker. The prepared membrane offered selectivity for the transport of S-ADP, and had the potential for separation of racemic mixtures.^214^ Székely et al. have fabricated a PBI-based molecularly imprinted membrane by phase inversion. The PBI acted as both the size-based membrane and shape-specific adsorbent. It showed both nanofiltration performance and excellent molecular recognition properties.^215^

### 5.4 Effect of Scale-up on the Sustainability of the OSN Process

Although initially the applications of OSN may be hindered by high capital costs, the steady improvements in fabrications of membranes and membrane modules, and the improvement of operational processes have lowered the capital cost drastically.^3^ When discussing the sustainability of OSN processes, the scale of operation plays an important role. In fact, both the productivity and operation time are favored by scaling up the process.^3^ Commercially available membrane modules provide higher productivity-to-size ratio^191^ and enhanced process intensification metrics because of their higher area-to-volume (A/V) ratio. For diafiltration processes, the required operation time also decreases significantly with increasing the scale, because of the higher A/V ratio. Additional considerations are the cost of pumps, pipework, and labor.^3^ As shown in equation 6 the cost-scale relationship has been reported to be inverse-exponential.
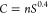
6Where *C* is the cost, *n* is the constant for different items, for example pumps, pipework, or labor. *S* is the scale. Also, the operational and cleaning cost should also be considered.

**Figure 26:**
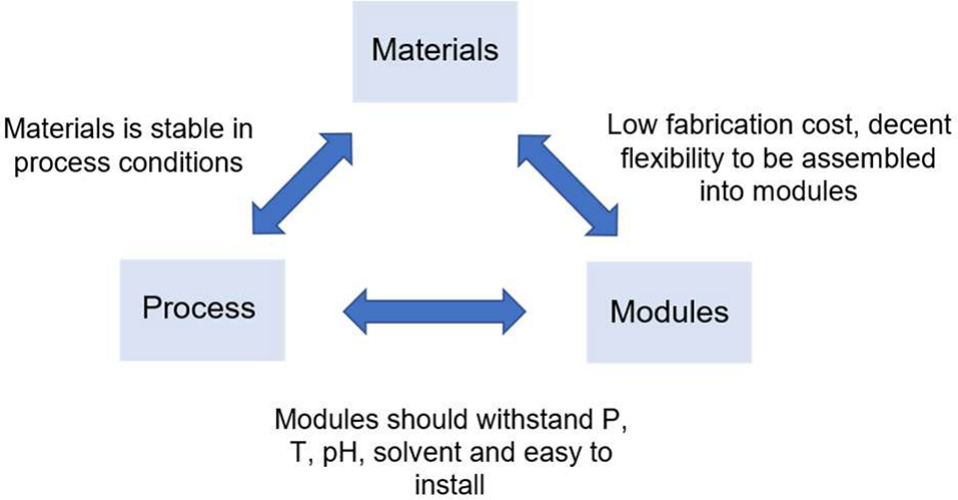
Interdependent factors for successful upscaling of the membrane process. Reproduced with permission from ref (3). Copyright 2014 The Royal Society of Chemistry.

There are three factors (Figure 26) that dictate the success and feasibility of large-scale membrane process: (a) membrane stability and performance; (b) fabrication technology for high packing density of membrane modules; (c) innovative process design tackling the drawbacks (e.g. pressure drop) and reducing the cost.^3^ They are interdependent and all of them must be addressed to achieve a successful large-scale membrane process.

Unfortunately, as the OSN field is still quite new in the pharmaceutical industry, and most OSN processes reported were performed on a lab scale, these do not provide sufficient information on the performance considering industrial related solutions and conditions such as long-term filtration performance.^216^ Almost one-third of OSN papers did not specify the operational time, and when specified, around 50% did not report process times longer than 24 h and only 8% of them had the process run for more than one week.^216^ As a result, there is a wide knowledge gap between academic results and industrial requirements. To facilitate the uptake of OSN in pharmaceutical industry applications, research attention should focus on sustainable scale-up and promoting the possibilities.^3^ One of the greatest hurdles to achieving the utilization of OSN in the pharmaceutical industry is a lack of knowledge about the separation mechanism caused by the sophisticated interactions among solutes, solvents and membranes.^217^ To tackle this, fundamental study of the transport phenomena and separation mechanism are of interest, since they provide opportunities for rational design of materials and optimization of process performance using currently available membranes.^216^ Another obstacle during the scaling-up process design is lacking experimental data to support the prediction model. To overcome this, machine learning could be one of the solutions.^218,219^

## 6 Conclusion

Organic solvent nanofiltration is a versatile, energy-saving, and cost-effective separation technique that possesses the potential to complement established separation processes. There are also cases where membrane technology is more practical than conventional processes, for example, processes which involve heat-sensitive molecules or exchange of nonvolatile solvents.^6^ Although the applications of OSN in the pharmaceutical industry have been extensively studied, the growth potential is still substantial and there are still many issues to resolve which impede the industry to switch from well-established processes to OSN.

The main challenge is insufficient separation performance, which limits the use of membrane in cases where two or more solutes with similar MWs are to be separated or where small solutes (<200 Da) need to be removed. To overcome this issue, novel membranes with lower molecular weight cutoff (MWCO) or enhanced selectivity toward different solutes need to be developed. The use of membrane cascade using commercially available membranes could also enhance the overall performance (enhanced selectivity and decreasing amount of solvent needed) of OSN. At the industrial level, the relationship between capital cost and plant footprint is one of the factors that affects the establishment of OSN.^6^ The difficulties of automation and control of continuous OSN processes are also obstacles to large-scale implementation. To develop more efficient OSN processes, it would be beneficial to have more dedicated models that can predict the performance of the membrane process.^6^ Analysis of sustainability metrics compared to conventional processes is recommended, including the CO_2_ footprint, mass intensity, solvent intensity, and energy consumption.

Despite the promising development of OSN in the past decade, there is still a huge space to be discovered. We can conclude that OSN is a good method for applications in the pharmaceutical industry to provide sustainable large-scale manufacturing due to its energy and cost efficiency and low CO_2_ footprint compared to conventional processes.

## Abbreviations

API: Active Pharmaceutical Ingredient
COF: Covalent organic framework
DMF: Dimethylformamide
GMP: Good manufacturing practice
GO: Graphene oxide
GTIs: Genotoxic impurities
ISA: Integrally skinned asymmetric
LPOS: Liquid phase oligonucleotide synthesis
LPPS: Liquid phase peptide synthesis
MI: Mass intensity
MPD: m-phenylenediamine
MW: Molecular Weight
MWCO: Molecular Weight Cut-Off
NHC: N-heterocyclic carbene
OSN: Organic Solvent Nanofiltration
PAN: Polyacrylonitrile
PBI: Polybenzimidazole
PDMS: Polydimethylsiloxane
PEEK: Polyether ether ketone
PEG: Polyethylene glycol
PEPSTAR: Liquid phase peptide synthesis via one-pot nanostar-sieving
PES: Polyethersulfone
PMI: Process Mass Intensity
SI: Solvent intensity
SPPS: Solid phase peptide synthesis
TFC: Thin film composite
THF: Tetrahydrofuran
TMS: Thermomorphic multicomponent solvent

## References

[ref1] Sholl D. S.; Lively R. P. Seven chemical separations to change the world. Nature 2016, 532 (7600), 435–437. 10.1038/532435a. 27121824

[ref2] Lively R. P.; Sholl D. S. From water to organics in membrane separations. Nat. Mater. 2017, 16 (3), 276–279. 10.1038/nmat4860. 28223707

[ref3] Székely G.; Jimenez-Solomon M. F.; Marchetti P.; Kim J. F.; Livingston A. G. Sustainability assessment of organic solvent nanofiltration: from fabrication to application. Green Chem. 2014, 16 (10), 4440–4473. 10.1039/C4GC00701H.

[ref4] Elimelech M.; Phillip W. A. The Future of Seawater Desalination: Energy, Technology, and the Environment. Science 2011, 333 (6043), 712–717. 10.1126/science.1200488. 21817042

[ref5] Constable D. J. C.; Jimenez-Gonzalez C.; Henderson R. K. Perspective on Solvent Use in the Pharmaceutical Industry. Org. Process Res. Dev. 2007, 11 (1), 133–137. 10.1021/op060170h.

[ref6] Marchetti P.; Jimenez Solomon M. F.; Székely G.; Livingston A. G. Molecular separation with organic solvent nanofiltration: a critical review. Chem. Rev. 2014, 114 (21), 10735–10806. 10.1021/cr500006j. 25333504

[ref7] Vandezande P.; Gevers L. E.; Vankelecom I. F. Solvent resistant nanofiltration: separating on a molecular level. Chem. Soc. Rev. 2008, 37 (2), 365–405. 10.1039/B610848M. 18197351

[ref8] Buonomenna M. G.; Bae J. Organic Solvent Nanofiltration in Pharmaceutical Industry. Separation & Purification Reviews 2015, 44 (2), 157–182. 10.1080/15422119.2014.918884.

[ref9] Loeb S.; Sourirajan S. Sea Water Demineralization by Means of an Osmotic Membrane. In Saline Water Conversion—II; Gould R. F., Ed.; Advances in Chemistry, Vol. 38; American Chemical Society, 1963; pp 117-132.

[ref10] Sourirajan S. Separation of Hydrocarbon Liquids by Flow Under Pressure Through Porous Membranes. Nature 1964, 203 (4952), 1348–1349. 10.1038/2031348a0.

[ref11] White L. S.; Nitsch A. R. Solvent recovery from lube oil filtrates with a polyimide membrane. J. Membr. Sci. 2000, 179 (1), 267–274. 10.1016/S0376-7388(00)00517-2.

[ref12] Jimenez Solomon M. F.; Bhole Y.; Livingston A. G. High flux membranes for organic solvent nanofiltration (OSN)—Interfacial polymerization with solvent activation. J. Membr. Sci. 2012, 423–424, 371–382. 10.1016/j.memsci.2012.08.030.

[ref13] Merlet R. B.; Pizzoccaro-Zilamy M.-A.; Nijmeijer A.; Winnubst L. Hybrid ceramic membranes for organic solvent nanofiltration: State-of-the-art and challenges. J. Membr. Sci. 2020, 599, 117839 10.1016/j.memsci.2020.117839.

[ref14] Ren D.; Ren S.; Lin Y.; Xu J.; Wang X. Recent developments of organic solvent resistant materials for membrane separations. Chemosphere 2021, 271, 129425 10.1016/j.chemosphere.2020.129425. 33445020

[ref15] Shi G. M.; Feng Y.; Li B.; Tham H. M.; Lai J.-Y.; Chung T.-S. Recent progress of organic solvent nanofiltration membranes. Prog. Polym. Sci. 2021, 123, 101470 10.1016/j.progpolymsci.2021.101470.

[ref16] Tandel A. M.; Guo W.; Bye K.; Huang L.; Galizia M.; Lin H. Designing organic solvent separation membranes: polymers, porous structures, 2D materials, and their combinations. Mater. Adv. 2021, 2 (14), 4574–4603. 10.1039/D1MA00373A.

[ref17] Wang Z.; Luo X.; Zhang J.; Zhang F.; Fang W.; Jin J. Polymer membranes for organic solvent nanofiltration: Recent progress, challenges and perspectives. Adv. Membr. 2023, 3, 100063 10.1016/j.advmem.2023.100063.

[ref18] Agrawal P.; Wilkstein K.; Guinn E.; Mason M.; Serrano Martinez C. I.; Saylae J. A Review of Tangential Flow Filtration: Process Development and Applications in the Pharmaceutical Industry. Org. Process Res. Dev. 2023, 27 (4), 571–591. 10.1021/acs.oprd.2c00291.

[ref19] Peeva L. G.; Gibbins E.; Luthra S. S.; White L. S.; Stateva R. P.; Livingston A. G. Effect of concentration polarisation and osmotic pressure on flux in organic solvent nanofiltration. J. Membr. Sci. 2004, 236 (1–2), 121–136. 10.1016/j.memsci.2004.03.004.

[ref20] Mulder M. Basic Principles of Membrane Technology; Springer Dordrecht, 1996.

[ref21] Schäfer A. I.; Fane A. G. Nanofiltration: Principles, applications, and new materials; John Wiley & Sons, 2021.

[ref22] Park H. B.; Kamcev J.; Robeson L. M.; Elimelech M.; Freeman B. D. Maximizing the right stuff: The trade-off between membrane permeability and selectivity. Science 2017, 356 (6343), eaab0530 10.1126/science.aab0530. 28619885

[ref23] Siew W. E.; Livingston A. G.; Ates C.; Merschaert A. Molecular separation with an organic solvent nanofiltration cascade - augmenting membrane selectivity with process engineering. Chem. Eng. Sci. 2013, 90, 299–310. 10.1016/j.ces.2012.10.028.

[ref24] Baker R. W. Membrane Technology and Applications; John Wiley & Sons, Ltd, 2012.

[ref25] Marchetti P.; Peeva L.; Livingston A. The Selectivity Challenge in Organic Solvent Nanofiltration: Membrane and Process Solutions. Annu. Rev. Chem. Biomol. Eng. 2017, 8, 473–497. 10.1146/annurev-chembioeng-060816-101325. 28511021

[ref26] Yang X. J.; Livingston A. G.; Freitas dos Santos L. Experimental observations of nanofiltration with organic solvents. J. Membr. Sci. 2001, 190 (1), 45–55. 10.1016/S0376-7388(01)00392-1.

[ref27] Verbeke R.; Nulens I.; Thijs M.; Lenaerts M.; Bastin M.; Van Goethem C.; Koeckelberghs G.; Vankelecom I. F. J. Solutes in solvent resistant and solvent tolerant nanofiltration: How molecular interactions impact membrane rejection. J. Membr. Sci. 2023, 677, 121595 10.1016/j.memsci.2023.121595.

[ref28] Wijmans J. G.; Baker R. W. The solution-diffusion model: a review. J. Membr. Sci. 1995, 107 (1), 1–21. 10.1016/0376-7388(95)00102-I.

[ref29] Abdulhamid M. A.; Székely G. Organic solvent nanofiltration membranes based on polymers of intrinsic microporosity. Curr. Opin. Chem. Eng. 2022, 36, 100804 10.1016/j.coche.2022.100804.

[ref30] Li W.; Chuah C. Y.; Nie L.; Bae T.-H. Enhanced CO2/CH4 selectivity and mechanical strength of mixed-matrix membrane incorporated with NiDOBDC/GO composite. J. Ind. Eng. Chem. 2019, 74, 118–125. 10.1016/j.jiec.2019.02.016.

[ref31] Valtcheva I. B.; Kumbharkar S. C.; Kim J. F.; Bhole Y.; Livingston A. G. Beyond polyimide: Crosslinked polybenzimidazole membranes for organic solvent nanofiltration (OSN) in harsh environments. J. Membr. Sci. 2014, 457, 62–72. 10.1016/j.memsci.2013.12.069.

[ref32] Lee J.; Yang H.; Bae T. H. Polybenzimidazole Membrane Crosslinked with Epoxy-Containing Inorganic Networks for Organic Solvent Nanofiltration and Aqueous Nanofiltration under Extreme Basic Conditions. Membranes 2022, 12 (2), 140 10.3390/membranes12020140. 35207063 PMC8877178

[ref33] Li S.; Meng Lin M.; Toprak M. S.; Kim D. K.; Muhammed M. Nanocomposites of polymer and inorganic nanoparticles for optical and magnetic applications. Nano Reviews 2010, 1 (1), 5214 10.3402/nano.v1i0.5214. PMC3215211 22110855

[ref34] Valtcheva I. B.; Marchetti P.; Livingston A. G. Crosslinked polybenzimidazole membranes for organic solvent nanofiltration (OSN): Analysis of crosslinking reaction mechanism and effects of reaction parameters. J. Membr. Sci. 2015, 493, 568–579. 10.1016/j.memsci.2015.06.056.

[ref35] Mertens M.; Van Goethem C.; Thijs M.; Koeckelberghs G.; Vankelecom I. F. J. Crosslinked PVDF-membranes for solvent resistant nanofiltration. J. Membr. Sci. 2018, 566, 223–230. 10.1016/j.memsci.2018.08.051.

[ref36] Da Silva Burgal J.; Peeva L.; Livingston A. Towards improved membrane production: Using low-toxicity solvents for the preparation of PEEK nanofiltration membranes. Green Chem. 2016, 18 (8), 2374–2384. 10.1039/C5GC02546J.

[ref37] Bastin M.; Raymenants J.; Thijs M.; Vananroye A.; Koeckelberghs G.; Vankelecom I. F. J. Epoxy-based solvent-tolerant nanofiltration membranes prepared via non-solvent induced phase inversion as novel class of stable membranes. J. Membr. Sci. 2021, 626, 119206 10.1016/j.memsci.2021.119206.

[ref38] Goh K. S.; Chen Y.; Chong J. Y.; Bae T. H.; Wang R. Thin film composite hollow fibre membrane for pharmaceutical concentration and solvent recovery. J. Membr. Sci. 2021, 621, 119008 10.1016/j.memsci.2020.119008.

[ref39] Wang C.; Park M. J.; Seo D. H.; Drioli E.; Matsuyama H.; Shon H. Recent advances in nanomaterial-incorporated nanocomposite membranes for organic solvent nanofiltration. Sep. Purif. Technol. 2021, 268, 118657 10.1016/j.seppur.2021.118657.

[ref40] Hermans S.; Dom E.; Mariën H.; Koeckelberghs G.; Vankelecom I. F. J. Efficient synthesis of interfacially polymerized membranes for solvent resistant nanofiltration. J. Membr. Sci. 2015, 476, 356–363. 10.1016/j.memsci.2014.11.046.

[ref41] Cadotte J. E.; Petersen R. J. Thin-Film Composite Reverse-Osmosis Membranes: Origin, Development, and Recent Advances. In Synthetic Membranes; Turbak A. F., Ed.; ACS Symposium Series, Vol. 153; American Chemical Society, 1981; pp 305–326.

[ref42] Fritsch D.; Merten P.; Heinrich K.; Lazar M.; Priske M. High performance organic solvent nanofiltration membranes: Development and thorough testing of thin film composite membranes made of polymers of intrinsic microporosity (PIMs). J. Membr. Sci. 2012, 401–402, 222–231. 10.1016/j.memsci.2012.02.008.

[ref43] Kujawa J.; Kujawski W.; Koter S.; Rozicka A.; Cerneaux S.; Persin M.; Larbot A. Efficiency of grafting of Al2O3, TiO2 and ZrO2 powders by perfluoroalkylsilanes. Colloids Surf., A 2013, 420, 64–73. 10.1016/j.colsurfa.2012.12.021.

[ref44] Rezaei Hosseinabadi S.; Wyns K.; Meynen V.; Carleer R.; Adriaensens P.; Buekenhoudt A.; Van der Bruggen B. Organic solvent nanofiltration with Grignard functionalised ceramic nanofiltration membranes. J. Membr. Sci. 2014, 454, 496–504. 10.1016/j.memsci.2013.12.032.

[ref45] Hosseinabadi S. R.; Wyns K.; Buekenhoudt A.; Van Der Bruggen B.; Ormerod D. Performance of Grignard functionalized ceramic nanofiltration membranes. Sep. Purif. Technol. 2015, 147, 320–328. 10.1016/j.seppur.2015.03.047.

[ref46] Cseri L.; Fodi T.; Kupai J.; T. Balogh G.; Garforth A.; Székely G. Membrane-assisted catalysis in organic media. Adv. Mater. Lett. 2017, 8 (12), 1094–1124. 10.5185/amlett.2017.1541.

[ref47] Ruthusree S.; Sundarrajan S.; Ramakrishna S. Progress and Perspectives on Ceramic Membranes for Solvent Recovery. Membranes 2019, 9 (10), 128 10.3390/membranes9100128. 31590261 PMC6835421

[ref48] Zeidler S.; Puhlfürß P.; Kätzel U.; Voigt I. Preparation and characterization of new low MWCO ceramic nanofiltration membranes for organic solvents. J. Membr. Sci. 2014, 470, 421–430. 10.1016/j.memsci.2014.07.051.

[ref49] Li Y.; Yu J. Emerging applications of zeolites in catalysis, separation and host-guest assembly. Nat. Rev. Mater. 2021, 6 (12), 1156–1174. 10.1038/s41578-021-00347-3.

[ref50] Smeets P. J.; Woertink J. S.; Sels B. F.; Solomon E. I.; Schoonheydt R. A. Transition-Metal Ions in Zeolites: Coordination and Activation of Oxygen. Inorg. Chem. 2010, 49 (8), 3573–3583. 10.1021/ic901814f. 20380459 PMC2881549

[ref51] McKean T.; Wickramasinghe R. Organic Solvent Recovery by Nanofiltration Membrane. In Nanofiltration for Sustainability; CRC Press, pp 209–233.

[ref52] U.S. Environmental Protection Agency. Profile of the Pharmaceutical Industry: Sector Notebook; National Service Center for Environmental Publications (NSCEP), 1997.

[ref53] Geens J.; De Witte B.; Van der Bruggen B. Removal of API’s (Active Pharmaceutical Ingredients) from Organic Solvents by Nanofiltration. Sep. Sci. Technol. 2007, 42 (11), 2435–2449. 10.1080/01496390701477063.

[ref54] Martínez M. B.; Van der Bruggen B.; Negrin Z. R.; Luis Alconero P. Separation of a high-value pharmaceutical compound from waste ethanol by nanofiltration. J. Ind. Eng. Chem. 2012, 18 (5), 1635–1641. 10.1016/j.jiec.2012.02.024.

[ref55] Saykova I.; Trayanov I.; Bojkova M.; Stoilova N.; Funeva-Peycheva M. Organic solvent nanofiltration of extracts from Hypericum Perforatum L.: Effect of variable feed composition on rejection and flux decline. Bulg. Chem. Commun. 2020, 52 (4), 525–531.

[ref56] Shi D.; Kong Y.; Yu J.; Wang Y.; Yang J. Separation performance of polyimide nanofiltration membranes for concentrating spiramycin extract. Desalination 2006, 191 (1–3), 309–317. 10.1016/j.desal.2005.09.015.

[ref57] Xu S. J.; Shen Q.; Xu Z. L.; Dong Z. Q. Novel designed TFC membrane based on host-guest interaction for organic solvent nanofiltration (OSN). J. Membr. Sci. 2019, 588, 117227 10.1016/j.memsci.2019.117227.

[ref58] Huang J. H.; Cheng X. Q.; Bai Q.; Zhang Y. J.; Wang K.; Ma J.; Shao L. Ultrafast Poly(sodium methacrylate)-Grafted UiO-66-Incorporated Nanocomposite Membranes Enable Excellent Active Pharmaceutical Ingredient Concentration. Ind. Eng. Chem. Res. 2021, 60 (17), 6287–6297. 10.1021/acs.iecr.1c00705.

[ref59] Li B.; Cui Y.; Japip S.; Thong Z.; Chung T. S. Graphene oxide (GO) laminar membranes for concentrating pharmaceuticals and food additives in organic solvents. Carbon 2018, 130, 503–514. 10.1016/j.carbon.2018.01.040.

[ref60] Kandambeth S.; Biswal B. P.; Chaudhari H. D.; Rout K. C.; Kunjattu H. S.; Mitra S.; Karak S.; Das A.; Mukherjee R.; Kharul U. K.; Banerjee R. Selective Molecular Sieving in Self-Standing Porous Covalent-Organic-Framework Membranes. Adv. Mater. 2017, 29 (2), 1603945 10.1002/adma.201603945. 28066986

[ref61] Shi X.; Zhang Z.; Yin C.; Zhang X.; Long J.; Zhang Z.; Wang Y. Design of Three-Dimensional Covalent Organic Framework Membranes for Fast and Robust Organic Solvent Nanofiltration. Angew. Chem., Int. Ed. 2022, 61 (36), e202207559 10.1002/anie.202207559. 35841536

[ref62] Siew W. E.; Livingston A. G.; Ates C.; Merschaert A. Continuous solute fractionation with membrane cascades - A high productivity alternative to diafiltration. Sep. Purif. Technol. 2013, 102, 1–14. 10.1016/j.seppur.2012.09.017.

[ref63] Buekenhoudt A.; Bulut M.; Beckers H.; Vleeschouwers R.; van Zanten D. OSN: Successful API recovery from a distillation residue at Sitech-DSM. In Proceedings of the Aachener Membrane Colloquium; AMK2014, Aachen, Germany2014.

[ref64] Priske M.; Lazar M.; Schnitzer C.; Baumgarten G. Recent Applications of Organic Solvent Nanofiltration. Chem. Ing. Tech. 2016, 88 (1–2), 39–49. 10.1002/cite.201500084.

[ref65] Buekenhoudt A.; Beckers H.; Ormerod D.; Bulut M.; Vandezande P.; Vleeschouwers R. Solvent Based Membrane Nanofiltration for Process Intensification. Chem. Ing. Tech. 2013, 85 (8), 1243–1247. 10.1002/cite.201200247.

[ref66] Guideline I. H. T. Impurities in new drug substances Q3A (R2). In Proceedings of the International Conference on Harmonization of Technical Requirements for Registration of Pharmaceuticals for Human Use, Geneva, Switzerland, 2006; Vol. 25.

[ref67] Kung-Tien L.; Chien-Hsin C. Determination of Impurities in Pharmaceuticals: Why and How? In Quality Management and Quality Control; Paulo P., Sandra X. Eds.; IntechOpen, 2019; pp 1–17.

[ref68] Orehek J.; Teslić D.; Likozar B. Continuous Crystallization Processes in Pharmaceutical Manufacturing: A Review. Org. Process Res. Dev. 2021, 25 (1), 16–42. 10.1021/acs.oprd.0c00398.

[ref69] Sereewatthanawut I.; Lim F. W.; Bhole Y. S.; Ormerod D.; Horvath A.; Boam A. T.; Livingston A. G. Demonstration of Molecular Purification in Polar Aprotic Solvents by Organic Solvent Nanofiltration. Org. Process Res. Dev. 2010, 14 (3), 600–611. 10.1021/op100028p.

[ref70] Robinson D. I. Control of Genotoxic Impurities in Active Pharmaceutical Ingredients: A Review and Perspective. Org. Process Res. Dev. 2010, 14 (4), 946–959. 10.1021/op900341a.

[ref71] Székely G.; Amores de Sousa M. C.; Gil M.; Castelo Ferreira F.; Heggie W. Genotoxic Impurities in Pharmaceutical Manufacturing: Sources, Regulations, and Mitigation. Chem. Rev. 2015, 115 (16), 8182–8229. 10.1021/cr300095f. 26252800

[ref72] Székely G.; Bandarra J.; Heggie W.; Sellergren B.; Ferreira F. C. Organic solvent nanofiltration: A platform for removal of genotoxins from active pharmaceutical ingredients. J. Membr. Sci. 2011, 381 (1), 21–33. 10.1016/j.memsci.2011.07.007.

[ref73] Székely G.; Gil M.; Sellergren B.; Heggie W.; Ferreira F. C. Environmental and economic analysis for selection and engineering sustainable API degenotoxification processes. Green Chem. 2013, 15 (1), 210–225. 10.1039/C2GC36239B.

[ref74] Rundquist E. M.; Pink C. J.; Livingston A. G. Organic solvent nanofiltration: a potential alternative to distillation for solvent recovery from crystallisation mother liquors. Green Chem. 2012, 14 (8), 2197–2205. 10.1039/c2gc35216h.

[ref75] Liu C.; Dong G.; Tsuru T.; Matsuyama H. Organic solvent reverse osmosis membranes for organic liquid mixture separation: A review. J. Membr. Sci. 2021, 620, 118882 10.1016/j.memsci.2020.118882.

[ref76] Kim J. F.; Szekely G.; Schaepertoens M.; Valtcheva I. B.; Jimenez-Solomon M. F.; Livingston A. G. In Situ Solvent Recovery by Organic Solvent Nanofiltration. ACS Sustainable Chem. Eng. 2014, 2 (10), 2371–2379. 10.1021/sc5004083.

[ref77] Kim J. F.; Freitas da Silva A. M.; Valtcheva I. B.; Livingston A. G. When the membrane is not enough: A simplified membrane cascade using Organic Solvent Nanofiltration (OSN). Sep. Purif. Technol. 2013, 116, 277–286. 10.1016/j.seppur.2013.05.050.

[ref78] Kim J. F.; Székely G.; Valtcheva I. B.; Livingston A. G. Increasing the sustainability of membrane processes through cascade approach and solvent recovery—pharmaceutical purification case study. Green Chem. 2014, 16 (1), 133–145. 10.1039/C3GC41402G.

[ref79] Peeva L.; Burgal J. d. S.; Valtcheva I.; Livingston A. G. Continuous purification of active pharmaceutical ingredients using multistage organic solvent nanofiltration membrane cascade. Chem. Eng. Sci. 2014, 116, 183–194. 10.1016/j.ces.2014.04.022.

[ref80] Vanneste J.; Ormerod D.; Theys G.; Van Gool D.; Van Camp B.; Darvishmanesh S.; Van der Bruggen B. Towards high resolution membrane-based pharmaceutical separations. J. Chem. Technol. Biotechnol. 2013, 88 (1), 98–108. 10.1002/jctb.3848.

[ref81] Székely G.; Bandarra J.; Heggie W.; Sellergren B.; Ferreira F. C. A hybrid approach to reach stringent low genotoxic impurity contents in active pharmaceutical ingredients: Combining molecularly imprinted polymers and organic solvent nanofiltration for removal of 1,3-diisopropylurea. Sep. Purif. Technol. 2012, 86, 79–87. 10.1016/j.seppur.2011.10.023.

[ref82] Ferreira F.; Resina L.; Esteves T.; Castelo Ferreira F. Comparison and combination of organic solvent nanofiltration and adsorption processes: A mathematical approach for mitigation of active pharmaceutical ingredient losses during genotoxin removal. Membranes 2020, 10 (4), 73 10.3390/membranes10040073. 32316155 PMC7231377

[ref83] Ormerod D.; Sledsens B.; Vercammen G.; Van Gool D.; Linsen T.; Buekenhoudt A.; Bongers B. Demonstration of purification of a pharmaceutical intermediate via organic solvent nanofiltration in the presence of acid. Sep. Purif. Technol. 2013, 115, 158–162. 10.1016/j.seppur.2013.05.007.

[ref84] Cole-Hamilton D. J. Homogeneous Catalysis-New Approaches to Catalyst Separation, Recovery, and Recycling. Science 2003, 299 (5613), 1702–1706. 10.1126/science.1081881. 12637737

[ref85] Cooper T. W. J.; Campbell I. B.; Macdonald S. J. F. Factors Determining the Selection of Organic Reactions by Medicinal Chemists and the Use of These Reactions in Arrays (Small Focused Libraries). Angew. Chem., Int. Ed. 2010, 49 (44), 8082–8091. 10.1002/anie.201002238. 20859975

[ref86] Garrett C. E.; Prasad K. The Art of Meeting Palladium Specifications in Active Pharmaceutical Ingredients Produced by Pd-Catalyzed Reactions. Adv. Synth. Catal. 2004, 346 (8), 889–900. 10.1002/adsc.200404071.

[ref87] Kisszékelyi P.; Nagy S.; Fehér Z.; Huszthy P.; Kupai J. Membrane-Supported Recovery of Homogeneous Organocatalysts: A Review. Chemistry (Switzerland) 2020, 2 (3), 742–758. 10.3390/chemistry2030048.

[ref88] Economidou M.; Mistry N.; Wheelhouse K. M. P.; Lindsay D. M. Palladium Extraction Following Metal-Catalyzed Reactions: Recent Advances and Applications in the Pharmaceutical Industry. Org. Process Res. Dev. 2023, 27 (9), 1585–1615. 10.1021/acs.oprd.3c00210.

[ref89] Vural Gürsel I.; Noël T.; Wang Q.; Hessel V. Separation/recycling methods for homogeneous transition metal catalysts in continuous flow. Green Chem. 2015, 17 (4), 2012–2026. 10.1039/C4GC02160F.

[ref90] Peddie W. L.; van Rensburg J. N.; Vosloo H. C. M.; van der Gryp P. Technological evaluation of organic solvent nanofiltration for the recovery of homogeneous hydroformylation catalysts. Chem. Eng. Res. Des. 2017, 121, 219–232. 10.1016/j.cherd.2017.03.015.

[ref91] Dreimann J. M.; Skiborowski M.; Behr A.; Vorholt A. J. Recycling Homogeneous Catalysts Simply by Organic Solvent Nanofiltration: New Ways to Efficient Catalysis. ChemCatChem 2016, 8 (21), 3330–3333. 10.1002/cctc.201601018.

[ref92] ICH. International Council for Harmonisation of Technical Requirements for Pharmaceuticals for Human Use (ICH) guideline Q3D (R2) on elemental impurities - Step 5; 2022.

[ref93] Giffels G.; Beliczey J.; Felder M.; Kragl U. Polymer enlarged oxazaborolidines in a membrane reactor: enhancing effectivity by retention of the homogeneous catalyst. Tetrahedron: Asymmetry 1998, 9 (4), 691–696. 10.1016/S0957-4166(98)00011-1.

[ref94] Felder M.; Giffels G.; Wandrey C. A polymer-enlarged homogeneously soluble oxazaborolidine catalyst for the asymmetric reduction of ketones by borane. Tetrahedron: Asymmetry 1997, 8 (12), 1975–1977. 10.1016/S0957-4166(97)00192-4.

[ref95] Lejeune A.; Le Goanvic L.; Renouard T.; Couturier J. L.; Dubois J. L.; Carpentier J. F.; Rabiller-Baudry M. Coupling Rhodium-Catalyzed Hydroformylation of 10-Undecenitrile with Organic Solvent Nanofiltration: Toluene Solution versus Solvent-Free Processes. ChemPlusChem 2019, 84 (11), 1744–1760. 10.1002/cplu.201900553. 31943870

[ref96] Ormerod D.; Lefevre N.; Dorbec M.; Eyskens I.; Vloemans P.; Duyssens K.; Diez de la Torre V.; Kaval N.; Merkul E.; Sergeyev S.; et al. Potential of Homogeneous Pd Catalyst Separation by Ceramic Membranes. Application to Downstream and Continuous Flow Processes. Org. Process Res. Dev. 2016, 20 (5), 911–920. 10.1021/acs.oprd.5b00418.

[ref97] Matthey J. PGM prices and trading. https://matthey.com/products-and-markets/pgms-and-circularity/pgm-management (accessed, 2023-06-10).

[ref98] Nair D.; Scarpello J. T.; White L. S.; Freitas dos Santos L. M.; Vankelecom I. F. J.; Livingston A. G. Semi-continuous nanofiltration-coupled Heck reactions as a new approach to improve productivity of homogeneous catalysts. Tetrahedron Lett. 2001, 42 (46), 8219–8222. 10.1016/S0040-4039(01)01734-8.

[ref99] Nair D.; Scarpello J. T.; Vankelecom I. F. J.; Freitas Dos Santos L. M.; White L. S.; Kloetzing R. J.; Welton T.; Livingston A. G. Increased catalytic productivity for nanofiltration-coupled Heck reactions using highly stable catalyst systems. Green Chem. 2002, 4 (4), 319–324. 10.1039/B203232P.

[ref100] Nair D.; Luthra S. S.; Scarpello J. T.; White L. S.; Freitas dos Santos L. M.; Livingston A. G. Homogeneous catalyst separation and re-use through nanofiltration of organic solvents. Desalination 2002, 147 (1), 301–306. 10.1016/S0011-9164(02)00556-8.

[ref101] Datta A.; Ebert K.; Plenio H. Nanofiltration for Homogeneous Catalysis Separation: Soluble Polymer-Supported Palladium Catalysts for Heck, Sonogashira, and Suzuki Coupling of Aryl Halides. Organometallics 2003, 22 (23), 4685–4691. 10.1021/om0303754.

[ref102] Dijkstra H. P.; Kruithof C. A.; Ronde N.; van de Coevering R.; Ramón D. J.; Vogt D.; van Klink G. P. M.; van Koten G. Shape-Persistent Nanosize Organometallic Complexes: Synthesis and Application in a Nanofiltration Membrane Reactor. J. Org. Chem. 2003, 68 (3), 675–685. 10.1021/jo0257602. 12558385

[ref103] Wong H.-t.; Pink C. J.; Ferreira F. C.; Livingston A. G. Recovery and reuse of ionic liquids and palladium catalyst for Suzuki reactions using organic solvent nanofiltration. Green Chem. 2006, 8 (4), 373 10.1039/b516778g.

[ref104] Pink C. J.; Wong H.-t.; Ferreira F. C.; Livingston A. G. Organic Solvent Nanofiltration and Adsorbents; A Hybrid Approach to Achieve Ultra Low Palladium Contamination of Post Coupling Reaction Products. Org. Process Res. Dev. 2008, 12 (4), 589–595. 10.1021/op800039g.

[ref105] Janssen M.; Müller C.; Vogt D. ‘Click’ Dendritic Phosphines: Design, Synthesis, Application in Suzuki Coupling, and Recycling by Nanofiltration. Adv. Synth. Catal. 2009, 351 (3), 313–318. 10.1002/adsc.200900058.

[ref106] Schoeps D.; Sashuk V.; Ebert K.; Plenio H. Solvent-Resistant Nanofiltration of Enlarged (NHC)Pd(allyl)Cl Complexes for Cross-Coupling Reactions. Organometallics 2009, 28 (13), 3922–3927. 10.1021/om900214j.

[ref107] Ronde N. J.; Totev D.; Müller C.; Lutz M.; Spek A. L.; Vogt D. Molecular-Weight-Enlarged Multiple-Pincer Ligands: Synthesis and Application in Palladium-Catalyzed Allylic Substitution Reactions. ChemSusChem 2009, 2 (6), 558–574. 10.1002/cssc.200800256. 19350608

[ref108] Tsoukala A.; Peeva L.; Livingston A. G.; Bjørsvik H.-R. Separation of Reaction Product and Palladium Catalyst after a Heck Coupling Reaction by means of Organic Solvent Nanofiltration. ChemSusChem 2012, 5 (1), 188–193. 10.1002/cssc.201100355. 22162431

[ref109] Peeva L.; Arbour J.; Livingston A. On the Potential of Organic Solvent Nanofiltration in Continuous Heck Coupling Reactions. Org. Process Res. Dev. 2013, 17 (7), 967–975. 10.1021/op400073p.

[ref110] Ormerod D.; Dorbec M.; Merkul E.; Kaval N.; Lefèvre N.; Hostyn S.; Eykens L.; Lievens J.; Sergeyev S.; Maes B. U. W. Synthesis of Pd Complexes Containing Tailed NHC Ligands and Their Use in a Semicontinuous Membrane-Assisted Suzuki Cross-Coupling Process. Org. Process Res. Dev. 2018, 22 (11), 1509–1517. 10.1021/acs.oprd.8b00273.

[ref111] Shen J.; Beale K.; Amura I.; Emanuelsson E. A. C. Ligand and Solvent Selection for Enhanced Separation of Palladium Catalysts by Organic Solvent Nanofiltration. Front. Chem. 2020, 8, 375 10.3389/fchem.2020.00375. 32432086 PMC7216237

[ref112] De Smet K.; Aerts S.; Ceulemans E.; Vankelecom I. F. J.; Jacobs P. A. Nanofiltration-coupled catalysis to combine the advantages of homogeneous and heterogeneous catalysis. Chem. Commun. (Cambridge, U. K.) 2001, 2001 (7), 597–598. 10.1039/b009898l.

[ref113] Janssen M.; Wilting J.; Müller C.; Vogt D. Continuous Rhodium-Catalyzed Hydroformylation of 1-Octene with Polyhedral Oligomeric Silsesquioxanes (POSS) Enlarged Triphenylphosphine. Angew. Chem., Int. Ed. 2010, 49 (42), 7738–7741. 10.1002/anie.201001926. 20827743

[ref114] Priske M.; Wiese K.-D.; Drews A.; Kraume M.; Baumgarten G. Reaction integrated separation of homogenous catalysts in the hydroformylation of higher olefins by means of organophilic nanofiltration. J. Membr. Sci. 2010, 360 (1), 77–83. 10.1016/j.memsci.2010.05.002.

[ref115] Shaharun M. S.; Mustafa A. K.; Taha M. F. Nanofiltration of rhodium tris(triphenylphosphine) catalyst in ethyl acetate solution. AIP Conf. Proc. 2012, 1482 (1), 279–283.

[ref116] Razak N. H. A.; Shaharun M. S.; Mukhtar H.; Taha M. F. Separation of hydridocarbonyltris(triphenylphosphine) rhodium (I)catalyst using solvent resistant nanofiltration membrane. Sains Malays. 2013, 42 (4), 515–520.

[ref117] Schmidt P.; Bednarz E. L.; Lutze P.; Górak A. Characterisation of Organic Solvent Nanofiltration membranes in multi-component mixtures: Process design workflow for utilising targeted solvent modifications. Chem. Eng. Sci. 2014, 115, 115–126. 10.1016/j.ces.2014.03.029.

[ref118] Dreimann J.; Lutze P.; Zagajewski M.; Behr A.; Górak A.; Vorholt A. J. Highly integrated reactor-separator systems for the recycling of homogeneous catalysts. Chem. Eng. Process. 2016, 99, 124–131. 10.1016/j.cep.2015.07.019.

[ref119] Dreimann J. M.; Hoffmann F.; Skiborowski M.; Behr A.; Vorholt A. J. Merging Thermomorphic Solvent Systems and Organic Solvent Nanofiltration for Hybrid Catalyst Recovery in a Hydroformylation Process. Ind. Eng. Chem. Res. 2017, 56 (5), 1354–1359. 10.1021/acs.iecr.6b04249.

[ref120] Scharzec B.; Holtkötter J.; Bianga J.; Dreimann J. M.; Vogt D.; Skiborowski M. Conceptual study of co-product separation from catalyst-rich recycle streams in thermomorphic multiphase systems by OSN. Chem. Eng. Res. Des. 2020, 157, 65–76. 10.1016/j.cherd.2020.02.028.

[ref121] Schlüter S.; Künnemann K. U.; Freis M.; Roth T.; Vogt D.; Dreimann J. M.; Skiborowski M. Continuous co-product separation by organic solvent nanofiltration for the hydroaminomethylation in a thermomorphic multiphase system. Chem. Eng. J. 2021, 409, 128219 10.1016/j.cej.2020.128219.

[ref122] Roengpithya C.; Patterson D. A.; Taylor P. C.; Livingston A. G. Development of stable organic solvent nanofiltration membranes for membrane enhanced dynamic kinetic resolution. Desalination 2006, 199 (1), 195–197. 10.1016/j.desal.2006.03.045.

[ref123] Wong H. T.; See-Toh Y. H.; Ferreira F. C.; Crook R.; Livingston A. G. Organic solvent nanofiltration in asymmetric hydrogenation: enhancement of enantioselectivity and catalyst stability by ionic liquids. Chem. Commun. (Camb.) 2006, 2006 (19), 2063–2065. 10.1039/b602184k. 16767276

[ref124] Keraani A.; Renouard T.; Fischmeister C.; Bruneau C.; Rabiller-Baudry M. Recovery of Enlarged Olefin Metathesis Catalysts by Nanofiltration in an Eco-Friendly Solvent. ChemSusChem 2008, 1 (11), 927–933. 10.1002/cssc.200800152. 18942694

[ref125] Nair D.; Wong H.-T.; Han S.; Vankelecom I. F. J.; White L. S.; Livingston A. G.; Boam A. T. Extending Ru-BINAP Catalyst Life and Separating Products from Catalyst Using Membrane Recycling. Org. Process Res. Dev. 2009, 13 (5), 863–869. 10.1021/op900056s.

[ref126] Schoeps D.; Buhr K.; Dijkstra M.; Ebert K.; Plenio H. Batchwise and Continuous Organophilic Nanofiltration of Grubbs-Type Olefin Metathesis Catalysts. Chem. - Eur. J. 2009, 15 (12), 2960–2965. 10.1002/chem.200802153. 19197933

[ref127] van der Gryp P.; Barnard A.; Cronje J.-P.; de Vlieger D.; Marx S.; Vosloo H. C. M. Separation of different metathesis Grubbs-type catalysts using organic solvent nanofiltration. J. Membr. Sci. 2010, 353 (1), 70–77. 10.1016/j.memsci.2010.02.032.

[ref128] Peeva L.; Livingston A. Potential of Organic Solvent Nanofiltration in Continuous Catalytic Reactions. Procedia Eng. 2012, 44, 307–309. 10.1016/j.proeng.2012.08.397.

[ref129] Kajetanowicz A.; Czaban J.; Krishnan G. R.; Malińska M.; Woźniak K.; Siddique H.; Peeva L. G.; Livingston A. G.; Grela K. Batchwise and Continuous Nanofiltration of POSS-Tagged Grubbs-Hoveyda-Type Olefin Metathesis Catalysts. ChemSusChem 2013, 6 (1), 182–192. 10.1002/cssc.201200466. 23086741

[ref130] Ormerod D.; Bongers B.; Porto-Carrero W.; Giegas S.; Vijt G.; Lefevre N.; Lauwers D.; Brusten W.; Buekenhoudt A. Separation of metathesis catalysts and reaction products in flow reactors using organic solvent nanofiltration. RSC Adv. 2013, 3 (44), 21501 10.1039/c3ra44860f.

[ref131] Rabiller-Baudry M.; Nasser G.; Renouard T.; Delaunay D.; Camus M. Comparison of two nanofiltration membrane reactors for a model reaction of olefin metathesis achieved in toluene. Sep. Purif. Technol. 2013, 116, 46–60. 10.1016/j.seppur.2013.04.052.

[ref132] Nasser G.; Renouard T.; Shahane S.; Fischmeister C.; Bruneau C.; Rabiller-Baudry M. Interest of the Precatalyst Design for Olefin Metathesis Operating in a Discontinuous Nanofiltration Membrane Reactor. ChemPlusChem 2013, 78 (7), 728–736. 10.1002/cplu.201300112. 31986634

[ref133] Guerra J.; Cantillo D.; Kappe C. O. Visible-light photoredox catalysis using a macromolecular ruthenium complex: Reactivity and recovery by size-exclusion nanofiltration in continuous flow. Catal. Sci. Technol. 2016, 6 (13), 4695–4699. 10.1039/C6CY00070C.

[ref134] Keraani A.; Nasser G.; Shahane S.; Renouard T.; Bruneau C.; Rabiller-Baudry M.; Fischmeister C. Syntheses and characterization of molecular weight enlarged olefin metathesis pre-catalysts. C. R. Chim. 2017, 20 (7), 717–723. 10.1016/j.crci.2017.01.001.

[ref135] Davood Abadi Farahani M. H.; Chung T. S. Solvent resistant hollow fiber membranes comprising P84 polyimide and amine-functionalized carbon nanotubes with potential applications in pharmaceutical, food, and petrochemical industries. Chem. Eng. J. 2018, 345, 174–185. 10.1016/j.cej.2018.03.153.

[ref136] Lejeune A.; Rabiller-Baudry M.; Vankelecom I.; Renouard T. On the relative influence of the hydrodynamics of lab-scale set-ups and the membrane materials on the rejection of homogeneous metal catalysts in solvent resistant nanofiltration. Sep. Sci. Technol. 2021, 56 (4), 766–778. 10.1080/01496395.2019.1706573.

[ref137] Mertens P. G. N.; Bulut M.; Gevers L. E. M.; Vankelecom I. F. J.; Jacobs P. A.; Vos D. E. D. Catalytic oxidation of 1,2-diols to α-hydroxy-carboxylates with stabilized gold nanocolloids combined with a membrane-based catalyst separation. Catal. Lett. 2005, 102 (1), 57–61. 10.1007/s10562-005-5203-9.

[ref138] Bayrakdar T. A. C. A.; Nahra F.; Zugazua O.; Eykens L.; Ormerod D.; Nolan S. P. Improving process efficiency of gold-catalyzed hydration of alkynes: merging catalysis with membrane separation. Green Chem. 2020, 22 (8), 2598–2604. 10.1039/D0GC00498G.

[ref139] Bayrakdar T. A. C. A.; Nahra F.; Ormerod D.; Nolan S. P. Integrating membrane separation with gold-catalyzed carboxylative cyclization of propargylamine and catalyst recovery via organic solvent nanofiltration. J. Chem. Technol. Biotechnol. 2021, 96 (12), 3371–3377. 10.1002/jctb.6892.

[ref140] Bayrakdar T. A. C. A.; Maliszewski B. P.; Nahra F.; Ormerod D.; Nolan S. P. Platinum-Catalyzed Alkene Hydrosilylation: Solvent-Free Process Development from Batch to a Membrane-Integrated Continuous Process. ChemSusChem 2021, 14 (18), 3810–3814. 10.1002/cssc.202101153. 34291872

[ref141] Witte P. T.; Chowdhury S. R.; ten Elshof J. E.; Sloboda-Rozner D.; Neumann R.; Alsters P. L. Highly efficient recycling of a “sandwich” type polyoxometalate oxidation catalyst using solvent resistant nanofiltration. Chem. Commun. (Cambridge, U. K.) 2005, (9), 1206–1208. 10.1039/B416096G. 15726193

[ref142] Roy Chowdhury S.; Witte P. T.; Blank D. H. A.; Alsters P. L.; ten Elshof J. E. Recovery of Homogeneous Polyoxometallate Catalysts from Aqueous and Organic Media by a Mesoporous Ceramic Membrane without Loss of Catalytic Activity. Chem. - Eur. J. 2006, 12 (11), 3061–3066. 10.1002/chem.200501021. 16440385

[ref143] Vondran J.; Peters M.; Schnettger A.; Sichelschmidt C.; Seidensticker T. From tandem to catalysis - organic solvent nanofiltration for catalyst separation in the homogeneously W-catalyzed oxidative cleavage of renewable methyl 9,10-dihydroxystearate. Catal. Sci. Technol. 2022, 12 (11), 3622–3633. 10.1039/D1CY02317A.

[ref144] Aerts S.; Buekenhoudt A.; Weyten H.; Gevers L. E. M.; Vankelecom I. F. J.; Jacobs P. A. The use of solvent resistant nanofiltration in the recycling of the Co-Jacobsen catalyst in the hydrolytic kinetic resolution (HKR) of epoxides. J. Membr. Sci. 2006, 280 (1), 245–252. 10.1016/j.memsci.2006.01.025.

[ref145] Cano-Odena A.; Vandezande P.; Fournier D.; Van Camp W.; Du Prez F. E.; Vankelecom I. F. J. Solvent-Resistant Nanofiltration for Product Purification and Catalyst Recovery in Click Chemistry Reactions. Chem. - Eur. J. 2010, 16 (3), 1061–1067. 10.1002/chem.200901659. 20013769

[ref146] Schnoor J. K.; Fuchs M.; Böcking A.; Wessling M.; Liauw M. A. Homogeneous Catalyst Recycling and Separation of a Multicomponent Mixture Using Organic Solvent Nanofiltration. Chem. Eng. Technol. 2019, 42 (10), 2187–2194. 10.1002/ceat.201900110.

[ref147] Schnoor J. K.; Bettmer J.; Kamp J.; Wessling M.; Liauw M. A. Recycling and separation of homogeneous catalyst from aqueous multicomponent mixture by organic solvent nanofiltration. Membranes 2021, 11 (6), 423 10.3390/membranes11060423. 34073034 PMC8230105

[ref148] Chavan S. A.; Maes W.; Gevers L. E. M.; Wahlen J.; Vankelecom I. F. J.; Jacobs P. A.; Dehaen W.; De Vos D. E. Porphyrin-Functionalized Dendrimers: Synthesis and Application as Recyclable Photocatalysts in a Nanofiltration Membrane Reactor. Chem. - Eur. J. 2005, 11 (22), 6754–6762. 10.1002/chem.200500251. 16134201

[ref149] Krupková A.; Kubátová K.; Št’astná L.; Cuřínová P.; Müllerová M.; Karban J.; Čermák J.; Strašák T. Poly(Imidazolium) carbosilane dendrimers: Synthesis, catalytic activity in redox esterification of α,β-unsaturated aldehydes and recycling via organic solvent nanofiltration. Catalysts 2021, 11 (11), 1317 10.3390/catal11111317.

[ref150] Kisszékelyi P.; Nagy S.; Tóth B.; Zeller B.; Hegedűs L.; Mátravölgyi B.; Grün A.; Németh T.; Huszthy P.; Kupai J. Synthesis and recovery of pyridine-and piperidine-based camphorsulfonamide organocatalysts used for michael addition reaction. Periodica Polytech., Chem. Eng. 2018, 62 (4), 489–496. 10.3311/PPch.12719.

[ref151] Großeheilmann J.; Büttner H.; Kohrt C.; Kragl U.; Werner T. Recycling of phosphorus-based organocatalysts by organic solvent nanofiltration. ACS Sustainable Chem. Eng. 2015, 3 (11), 2817–2822. 10.1021/acssuschemeng.5b00734.

[ref152] Luthra S. S.; Yang X.; Freitas dos Santos L. M.; White L. S.; Livingston A. G. Homogeneous phase transfer catalyst recovery and re-use using solvent resistant membranes. J. Membr. Sci. 2002, 201 (1), 65–75. 10.1016/S0376-7388(01)00704-9.

[ref153] Fahrenwaldt T.; Großeheilmann J.; Erben F.; Kragl U. Organic Solvent Nanofiltration as a Tool for Separation of Quinine-Based Organocatalysts. Org. Process Res. Dev. 2013, 17 (9), 1131–1136. 10.1021/op400037h.

[ref154] Siew W. E.; Ates C.; Merschaert A.; Livingston A. G. Efficient and productive asymmetric Michael addition: development of a highly enantioselective quinidine-based organocatalyst for homogeneous recycling via nanofiltration. Green Chem. 2013, 15 (3), 663–674. 10.1039/c2gc36407g.

[ref155] Großeheilmann J.; Fahrenwaldt T.; Kragl U. Organic solvent nanofiltration-supported purification of organocatalysts. ChemCatChem 2016, 8 (2), 322–325. 10.1002/cctc.201500902.

[ref156] Devendar P.; Qu R. Y.; Kang W. M.; He B.; Yang G. F. Palladium-Catalyzed Cross-Coupling Reactions: A Powerful Tool for the Synthesis of Agrochemicals. J. Agric. Food Chem. 2018, 66 (34), 8914–8934. 10.1021/acs.jafc.8b03792. 30060657

[ref157] Baumann M.; Moody T. S.; Smyth M.; Wharry S. A Perspective on Continuous Flow Chemistry in the Pharmaceutical Industry. Org. Process Res. Dev. 2020, 24 (10), 1802–1813. 10.1021/acs.oprd.9b00524.

[ref158] Peeva L.; da Silva Burgal J.; Vartak S.; Livingston A. G. Experimental strategies for increasing the catalyst turnover number in a continuous Heck coupling reaction. J. Catal. 2013, 306, 190–201. 10.1016/j.jcat.2013.06.020.

[ref159] Chauvin Y. Olefin Metathesis: The Early Days (Nobel Lecture). Angew. Chem., Int. Ed. 2006, 45 (23), 3740–3747. 10.1002/anie.200601234. 16724296

[ref160] Fang J.; Jana R.; Tunge J. A.; Subramaniam B. Continuous homogeneous hydroformylation with bulky rhodium catalyst complexes retained by nano-filtration membranes. Applied Catalysis A: General 2011, 393 (1), 294–301. 10.1016/j.apcata.2010.12.011.

[ref161] Dreimann J. M.; Vorholt A. J.; Skiborowski M.; Behr A. Removal of homogeneous precious metal catalysts via Organic solvent nanofiltration. Chem. Eng. Trans. 2016, 47, 343–348.

[ref162] Behr A.; Henze G.; Schomäcker R. Thermoregulated Liquid/Liquid Catalyst Separation and Recycling. Adv. Synth. Catal. 2006, 348 (12–13), 1485–1495. 10.1002/adsc.200606094.

[ref163] Kim J. F.; Gaffney P. R. J.; Valtcheva I. B.; Williams G.; Buswell A. M.; Anson M. S.; Livingston A. G. Organic Solvent Nanofiltration (OSN): A New Technology Platform for Liquid-Phase Oligonucleotide Synthesis (LPOS). Org. Process Res. Dev. 2016, 20 (8), 1439–1452. 10.1021/acs.oprd.6b00139.

[ref164] Gaffney P. R. J.; Kim J. F.; Valtcheva I. B.; Williams G. D.; Anson M. S.; Buswell A. M.; Livingston A. G. Liquid-Phase Synthesis of 2′-Methyl-RNA on a Homostar Support through Organic-Solvent Nanofiltration. Chem. - Eur. J. 2015, 21 (26), 9535–9543. 10.1002/chem.201501001. 26012874 PMC4517100

[ref165] Castro V.; Noti C.; Chen W.; Cristau M.; Livignston A.; Rodríguez H.; Albericio F. Novel Globular Polymeric Supports for Membrane-Enhanced Peptide Synthesis. Macromolecules 2017, 50 (4), 1626–1634. 10.1021/acs.macromol.6b02258.

[ref166] So S.; Peeva L. G.; Tate E. W.; Leatherbarrow R. J.; Livingston A. G. Membrane enhanced peptide synthesis. Chem. Commun. (Camb.) 2010, 46 (16), 2808–2810. 10.1039/b926747f. 20369190

[ref167] So S.; Peeva L. G.; Tate E. W.; Leatherbarrow R. J.; Livingston A. G. Organic Solvent Nanofiltration: A New Paradigm in Peptide Synthesis. Org. Process Res. Dev. 2010, 14 (6), 1313–1325. 10.1021/op1001403.

[ref168] Yeo J.; Peeva L.; Chung S.; Gaffney P.; Kim D.; Luciani C.; Tsukanov S.; Seibert K.; Kopach M.; Albericio F.; et al. Liquid Phase Peptide Synthesis via One-Pot Nanostar Sieving (PEPSTAR). Angew. Chem., Int. Ed. 2021, 60 (14), 7786–7795. 10.1002/anie.202014445. PMC8049079 33444460

[ref169] Dong R.; Liu R.; Gaffney P. R. J.; Schaepertoens M.; Marchetti P.; Williams C. M.; Chen R.; Livingston A. G. Sequence-defined multifunctional polyethers via liquid-phase synthesis with molecular sieving. Nat. Chem. 2019, 11 (2), 136–145. 10.1038/s41557-018-0169-6. 30510218

[ref170] Henninot A.; Collins J. C.; Nuss J. M. The Current State of Peptide Drug Discovery: Back to the Future?. J. Med. Chem. 2018, 61 (4), 1382–1414. 10.1021/acs.jmedchem.7b00318. 28737935

[ref171] Wang L.; Wang N.; Zhang W.; Cheng X.; Yan Z.; Shao G.; Wang X.; Wang R.; Fu C. Therapeutic peptides: current applications and future directions. Signal Transduction Targeted Ther. 2022, 7 (1), 48 10.1038/s41392-022-00904-4. PMC8844085 35165272

[ref172] Hyde C.; Johnson T.; Sheppard R. C. Internal aggregation during solid phase peptide synthesis. Dimethyl sulfoxide as a powerful dissociating solvent. J. Chem. Soc., Chem. Commun. 1992, 1992 (21), 1573–1575. 10.1039/c39920001573.

[ref173] Bayer E.; Mutter M. Liquid phase synthesis of peptides. Nature 1972, 237 (5357), 512–513. 10.1038/237512a0. 12635201

[ref174] Székely G.; Schaepertoens M.; Gaffney P. R. J.; Livingston A. G. Iterative synthesis of monodisperse PEG homostars and linear heterobifunctional PEG. Polym. Chem. 2014, 5 (3), 694–697. 10.1039/C3PY01367G.

[ref175] Roberts T. C.; Langer R.; Wood M. J. A. Advances in oligonucleotide drug delivery. Nat. Rev. Drug Discovery 2020, 19 (10), 673–694. 10.1038/s41573-020-0075-7. 32782413 PMC7419031

[ref176] Andrews B. I.; Antia F. D.; Brueggemeier S. B.; Diorazio L. J.; Koenig S. G.; Kopach M. E.; Lee H.; Olbrich M.; Watson A. L. Sustainability Challenges and Opportunities in Oligonucleotide Manufacturing. J. Org. Chem. 2021, 86 (1), 49–61. 10.1021/acs.joc.0c02291. 33253568 PMC8154579

[ref177] Gravert D. J.; Janda K. D. Organic Synthesis on Soluble Polymer Supports: Liquid-Phase Methodologies. Chem. Rev. 1997, 97 (2), 489–510. 10.1021/cr960064l. 11848880

[ref178] Lin J. C.-T.; Livingston A. G. Nanofiltration membrane cascade for continuous solvent exchange. Chem. Eng. Sci. 2007, 62 (10), 2728–2736. 10.1016/j.ces.2006.08.004.

[ref179] Peeva L.; Da Silva Burgal J.; Heckenast Z.; Brazy F.; Cazenave F.; Livingston A. Continuous Consecutive Reactions with Inter-Reaction Solvent Exchange by Membrane Separation. Angew. Chem., Int. Ed. Engl. 2016, 55 (43), 13576–13579. 10.1002/anie.201607795. 27669675 PMC5113664

[ref180] Anjum F.; Wessner M.; Sadowski G. Membrane-Based Solvent Exchange Process for Purification of API Crystal Suspensions. Membranes (Basel) 2023, 13 (3), 263 10.3390/membranes13030263. 36984651 PMC10058991

[ref181] Sheth J. P.; Qin Y.; Sirkar K. K.; Baltzis B. C. Nanofiltration-based diafiltration process for solvent exchange in pharmaceutical manufacturing. J. Membr. Sci. 2003, 211 (2), 251–261. 10.1016/S0376-7388(02)00423-4.

[ref182] Rundquist E.; Pink C.; Vilminot E.; Livingston A. Facilitating the use of counter-current chromatography in pharmaceutical purification through use of organic solvent nanofiltration. J. Chromatogr. A 2012, 1229, 156–163. 10.1016/j.chroma.2012.01.021. 22326182

[ref183] Wen Z.; Pintossi D.; Nuño M.; Noël T. Membrane-based TBADT recovery as a strategy to increase the sustainability of continuous-flow photocatalytic HAT transformations. Nat. Commun. 2022, 13 (1), 6147 10.1038/s41467-022-33821-9. 36257941 PMC9579200

[ref184] Lejeune A.; Rabiller-Baudry M.; Renouard T. Design of membrane cascades according to the method of McCabe-Thiele: An organic solvent nanofiltration case study for olefin hydroformylation in toluene. Sep. Purif. Technol. 2018, 195, 339–357. 10.1016/j.seppur.2017.12.031.

[ref185] Lightfoot E. N.; Root T. W.; L. O'Dell J. Emergence of Ideal Membrane Cascades for Downstream Processing. Biotechnol. Prog. 2008, 24 (3), 599–605. 10.1021/bp070335l. 18410154

[ref186] Abejón R.; Garea A.; Irabien A. Analysis and optimization of continuous organic solvent nanofiltration by membrane cascade for pharmaceutical separation. AIChE J. 2014, 60 (3), 931–948. 10.1002/aic.14345.

[ref187] Ghosh R. Novel cascade ultrafiltration configuration for continuous, high-resolution protein-protein fractionation: a simulation study. J. Membr. Sci. 2003, 226 (1–2), 85–99. 10.1016/j.memsci.2003.08.012.

[ref188] Roberge D. M.; Ducry L.; Bieler N.; Cretton P.; Zimmermann B. Microreactor Technology: A Revolution for the Fine Chemical and Pharmaceutical Industries?. Chem. Eng. Technol. 2005, 28 (3), 318–323. 10.1002/ceat.200407128.

[ref189] Ott D.; Kralisch D.; Denčić I.; Hessel V.; Laribi Y.; Perrichon P. D.; Berguerand C.; Kiwi-Minsker L.; Loeb P. Life Cycle Analysis within Pharmaceutical Process Optimization and Intensification: Case Study of Active Pharmaceutical Ingredient Production. ChemSusChem 2014, 7 (12), 3521–3533. 10.1002/cssc.201402313. 25251078

[ref190] Dunn P. J.; Wells A. S.; Williams M. T. Future Trends for Green Chemistry in the Pharmaceutical Industry. In Green Chemistry in the Pharmaceutical Industry; Dunn P. J., Wells A. S., Williams M. T., Eds.; Wiley-VCH, 2010; pp 333–355.

[ref191] Criscuoli A.; Drioli E. New Metrics for Evaluating the Performance of Membrane Operations in the Logic of Process Intensification. Ind. Eng. Chem. Res. 2007, 46 (8), 2268–2271. 10.1021/ie0610952.

[ref192] Waheed A.; Baig U. Exploiting phase inversion for penta-amine impregnation of ultrafiltration support matrix for rapid fabrication of a hyper-cross-linked polyamide membrane for organic solvent nanofiltration. Process Saf. Environ. Prot. 2023, 169, 24–33. 10.1016/j.psep.2022.10.072.

[ref193] Schaepertoens M.; Didaskalou C.; Kim J. F.; Livingston A. G.; Székely G. Solvent recycle with imperfect membranes: A semi-continuous workaround for diafiltration. J. Membr. Sci. 2016, 514, 646–658. 10.1016/j.memsci.2016.04.056.

[ref194] Asadi Tashvigh A.; Chung T. S. Robust polybenzimidazole (PBI) hollow fiber membranes for organic solvent nanofiltration. J. Membr. Sci. 2019, 572, 580–587. 10.1016/j.memsci.2018.11.048.

[ref195] Fodi T.; Didaskalou C.; Kupai J.; Balogh G. T.; Huszthy P.; Székely G. Nanofiltration-Enabled In Situ Solvent and Reagent Recycle for Sustainable Continuous-Flow Synthesis. ChemSusChem 2017, 10 (17), 3435–3444. 10.1002/cssc.201701120. 28737002 PMC6032941

[ref196] Ormerod D.; Noten B.; Dorbec M.; Andersson L.; Buekenhoudt A.; Goetelen L. Cyclic Peptide Formation in Reduced Solvent Volumes via In-Line Solvent Recycling by Organic Solvent Nanofiltration. Org. Process Res. Dev. 2015, 19 (7), 841–848. 10.1021/acs.oprd.5b00103.

[ref197] Ramesh P.; Karla S.; Alshehri A.; Yu M.; Kilduff J.; Belfort G. Stiffening Polymer Brush Membranes for Enhanced Organic Solvent Nanofiltration Selectivity. ACS Appl. Mater. Interfaces 2023, 15 (26), 31966–31978. 10.1021/acsami.3c04265. 37341440

[ref198] Ramesh P.; Sorci M.; Sengupta B.; Karla S.; Hao Z.; Yu M.; Kilduff J.; Belfort G. Highly tunable structure-by-design polymer brush membranes for organic solvent nanofiltration. J. Membr. Sci. 2023, 678, 121656 10.1016/j.memsci.2023.121656.

[ref199] Wang S.; Wang Z.; Zhu S.; Liu S.; Zhang F.; Jin J. Highly porous ultrathin polyamide membranes for fast separation of small molecules from organic solvents. J. Membr. Sci. 2023, 675, 121540 10.1016/j.memsci.2023.121540.

[ref200] Shin S. J.; Park Y. I.; Park H.; Cho Y. H.; Won G. Y.; Yoo Y. A facile crosslinking method for polybenzimidazole membranes toward enhanced organic solvent nanofiltration performance. Sep. Purif. Technol. 2022, 299, 121783 10.1016/j.seppur.2022.121783.

[ref201] Karan S.; Jiang Z.; Livingston A. G. Sub-10 nm polyamide nanofilms with ultrafast solvent transport for molecular separation. Science 2015, 348 (6241), 1347–1351. 10.1126/science.aaa5058. 26089512

[ref202] Xu S. J.; Shen Q.; Tong Y. H.; Dong Z. Q.; Xu Z. L. GWF-NH2 enhanced OSN membrane with trifluoromethyl groups in polyamide layer for rapid methanol recycling. Sep. Purif. Technol. 2020, 240, 116619 10.1016/j.seppur.2020.116619.

[ref203] Wu M. B.; Yang F.; Yang J.; Zhong Q.; Körstgen V.; Yang P.; Müller-Buschbaum P.; Xu Z. K. Lysozyme Membranes Promoted by Hydrophobic Substrates for Ultrafast and Precise Organic Solvent Nanofiltration. Nano Lett. 2020, 20 (12), 8760–8767. 10.1021/acs.nanolett.0c03632. 33211495

[ref204] Kang J.; Ko Y.; Kim J. P.; Kim J. Y.; Kim J.; Kwon O.; Kim K. C.; Kim D. W. Microwave-assisted design of nanoporous graphene membrane for ultrafast and switchable organic solvent nanofiltration. Nat. Commun. 2023, 14 (1), 901 10.1038/s41467-023-36524-x. 36797272 PMC9935848

[ref205] Francis V. N.; Chong J. Y.; Yang G.; Che L.; Wang R. Robust polyamide-PTFE hollow fibre membranes for harsh organic solvent nanofiltration. Chem. Eng. J. 2023, 452, 139333 10.1016/j.cej.2022.139333.

[ref206] Sadeghi I.; Kaner P.; Asatekin A. Controlling and Expanding the Selectivity of Filtration Membranes. Chem. Mater. 2018, 30 (21), 7328–7354. 10.1021/acs.chemmater.8b03334.

[ref207] Khan Z.; Long X.; Casey E.; Dowling D.; Ferguson S. Development of continuous spatially distributed diafiltration unit operations. React. Chem. Eng. 2023, 8 (7), 1785–1798. 10.1039/D3RE00013C.

[ref208] Hosseinabadi S. R.; Wyns K.; Meynen V.; Buekenhoudt A.; Van der Bruggen B. Solvent-membrane-solute interactions in organic solvent nanofiltration (OSN) for Grignard functionalised ceramic membranes: Explanation via Spiegler-Kedem theory. J. Membr. Sci. 2016, 513, 177–185. 10.1016/j.memsci.2016.04.044.

[ref209] Liu T.; Li Z.; Wang J.; Chen J.; Guan M.; Qiu H. Solid membranes for chiral separation: A review. Chem. Eng. J. 2021, 410, 128247 10.1016/j.cej.2020.128247.

[ref210] Ong C. S.; Oor J. Z.; Tan S. J.; Chew J. W. Enantiomeric Separation of Racemic Mixtures Using Chiral-Selective and Organic-Solvent-Resistant Thin-Film Composite Membranes. ACS Appl. Mater. Interfaces 2022, 14 (8), 10875–10885. 10.1021/acsami.1c25175. 35175724

[ref211] Zhu Y.; Li X.; Bai Z.; Zeng Y.; Jiang H.; Bai X.; Li R. Fabrication and application of graphene oxide modified cyclodextrin chiral separation membranes. New J. Chem. 2023, 47 (25), 11852–11858. 10.1039/D3NJ01309J.

[ref212] Milovanovic M.; Tabakoglu F.; Saki F.; Pohlkoetter E.; Buga D.; Brandt V.; Tiller J. C. Organic-inorganic double networks as highly permeable separation membranes with a chiral selector for organic solvents. J. Membr. Sci. 2023, 668, 121190 10.1016/j.memsci.2022.121190.

[ref213] Yoshikawa M.; Tharpa K.; Dima Ş.-O. Molecularly Imprinted Membranes: Past, Present, and Future. Chem. Rev. 2016, 116 (19), 11500–11528. 10.1021/acs.chemrev.6b00098. 27610706

[ref214] Men J.; Dong C.; Shi H.; Han Y.; Yang Y.; Wang R.; Wang X.; Chen J. Surface molecular imprinted membranes as a “gate” for selective transdermal release of chiral drug amlodipine. J. Membr. Sci. 2022, 664, 121059 10.1016/j.memsci.2022.121059.

[ref215] Székely G.; Valtcheva I. B.; Kim J. F.; Livingston A. G. Molecularly imprinted organic solvent nanofiltration membranes - Revealing molecular recognition and solute rejection behaviour. React. Funct. Polym. 2015, 86, 215–224. 10.1016/j.reactfunctpolym.2014.03.008.

[ref216] Le Phuong H. A.; Blanford C. F.; Székely G. Reporting the unreported: the reliability and comparability of the literature on organic solvent nanofiltration. Green Chem. 2020, 22 (11), 3397–3409. 10.1039/D0GC00775G.

[ref217] Henderson R. K.; Jiménez-González C.; Constable D. J. C.; Alston S. R.; Inglis G. G. A.; Fisher G.; Sherwood J.; Binks S. P.; Curzons A. D. Expanding GSK’s solvent selection guide - embedding sustainability into solvent selection starting at medicinal chemistry. Green Chem. 2011, 13 (4), 854–862. 10.1039/c0gc00918k.

[ref218] Ignacz G.; Székely G. Deep learning meets quantitative structure-activity relationship (QSAR) for leveraging structure-based prediction of solute rejection in organic solvent nanofiltration. J. Membr. Sci. 2022, 646, 120268 10.1016/j.memsci.2022.120268.

[ref219] Ignacz G.; Yang C.; Székely G. Diversity matters: Widening the chemical space in organic solvent nanofiltration. J. Membr. Sci. 2022, 641, 119929 10.1016/j.memsci.2021.119929.

